# Supplementary material for: Molecular insights into the unusually promiscuous and catalytically versatile Fe(II)/α-ketoglutarate-dependent oxygenase SptF
Source: Nat Commun. 2022 Jan 10;13:95. doi: 10.1038/s41467-021-27636-3 (PMC8748661; doi:10.1038/s41467-021-27636-3)
Supplement: Supplementary file 1 — Supplementary Information [file 41467_2021_27636_MOESM1_ESM.pdf]

Supplementary Information

Molecular insights into the unusually promiscuous and catalytically versatile Fe(II)/α-ketoglutarate-dependent oxygenase SptF

Hui Tao[1,5], Takahiro Mori[1,2,3,5,*], Heping Chen[1], Shuang Lyu[1], Akihito Nonoyama[4], Shoukou Lee[4], Ikuro Abe[1,2,*]

[1] Graduate School of Pharmaceutical Sciences, The University of Tokyo, Tokyo, Japan

[2] Collaborative Research Institute for Innovative Microbiology, The University of Tokyo, Tokyo, Japan

[3] PRESTO, Japan Science and Technology Agency, Saitama, Japan

[4] Sumitomo Dainippon Pharma Co., Ltd., Osaka, Japan

[5] These authors contributed equally

*Address correspondence to tmori@mol.f.u-tokyo.ac.jp or abei@mol.f.u-tokyo.ac.jp

Table of Contents

Supplementary Methods **S1-S2**

Supplementary Tables 1-20 **S3-S23**

Supplementary Figures 1-94 **S24-S79**

Supplementary References **S80**

Supplementary Methods

Chemical properties of key products:

Compound **4**: white powder, $[\alpha]^{26}_{D}$ -30.7 (*c* 0.03, MeOH). HRMS *m/z* 441.1915 $[M + H]^+$ (*calc.* 441.1908 for $C_{25}H_{29}O_7$) (Supplementary Fig. 2). ^1H and ^{13}C NMR data, see Supplementary Table 7; NMR spectrum, see Supplementary Figs. 19-24.

Compound **5**: white powder, $[\alpha]^{26}_{D}$ -22.6 (*c* 0.04, MeOH). HRMS *m/z* 441.1907 $[M + H]^+$ (*calc.* 441.1908 for $C_{25}H_{29}O_7$) (Supplementary Fig. 2). ^1H and ^{13}C NMR data, see Supplementary Table 8; NMR spectrum, see Supplementary Figs. 25-30.

Compound **8**: white powder, $[\alpha]^{26}_{D}$ +15.8 (*c* 0.02, MeOH). HRMS *m/z* 427.2113 $[M + H]^+$ (*calc.* 427.2115 for $C_{25}H_{31}O_6$) (Supplementary Fig. 2). ^1H and ^{13}C NMR data, see Supplementary Table 9; NMR spectrum, see Supplementary Figs. 31-36.

Compound **9**: white powder, $[\alpha]^{26}_{D}$ -20.3 (*c* 0.04, MeOH). HRMS *m/z* 427.2115 $[M + H]^+$ (*calc.* 427.2115 for $C_{25}H_{31}O_6$) (Supplementary Fig. 2). ^1H and ^{13}C NMR data, see Supplementary Table 10; NMR spectrum, see Supplementary Figs. 37-42.

Compound **11**: white powder, $[\alpha]^{26}_{D}$ -22.2 (*c* 0.08, MeOH). HRMS *m/z* 429.2259 $[M + H]^+$ (*calc.* 429.2272 for $C_{25}H_{33}O_6$) (Supplementary Fig. 2). ^1H and ^{13}C NMR data, see Supplementary Table 11; NMR spectrum, see Supplementary Figs. 43-48.

Compound **14**: white powder, $[\alpha]^{26}_{D}$ -17.8 (*c* 0.05, MeOH). HRMS *m/z* 430.2243 $[M + NH_4]^+$ (*calc.* 430.2224 for $C_{24}H_{32}NO_6$) (Supplementary Fig. 2). ^1H and ^{13}C NMR data, see Supplementary Table 12; NMR spectrum, see Supplementary Figs. 49-54.

Compound **16**: white powder, $[\alpha]^{26}_{D}$ -88.4 (*c* 0.08, MeOH). HRMS *m/z* 429.1899 $[M + H]^+$ (*calc.* 429.1908 for $C_{24}H_{29}O_7$) (Supplementary Fig. 2). ^1H and ^{13}C NMR data, see Supplementary Table 13; NMR spectrum, see Supplementary Figs. 55-60.

Compound **22**: white powder, HRMS *m/z* 307.2254 $[M + H]^+$ (*calc.* 307.2268 for $C_{19}H_{31}O_3$) (Supplementary Fig. 2). ^1H and ^{13}C NMR data, see Supplementary Table 14; NMR spectrum, see Supplementary Figs. 61-66.

Compound **23**: white powder, HRMS *m/z* 323.2215 $[M + H]^+$ (*calc.* 323.2217 for $C_{19}H_{31}O_4$) (Supplementary Fig. 2). ^1H and ^{13}C NMR data, see Supplementary Table 15; NMR spectrum, see Supplementary Figs. 67-72.

Compound **25**: white powder, HRMS *m/z* 305.2115 $[M + H]^+$ (*calc.* 305.2111 for $C_{19}H_{29}O_3$) (Supplementary Fig. 2). ^1H and ^{13}C NMR data, see Supplementary Table 16; NMR spectrum, see Supplementary Figs. 73-76. Crystalline sponge data, see Supplementary Table 4 and Supplementary Fig. 4.

Compound **26/27**: white powder, HRMS *m/z* 303.1963 $[M + H]^+$ (*calc.* 303.1955 for $C_{19}H_{27}O_3$) (Supplementary Fig. 2). ^1H and ^{13}C NMR data, see Supplementary Table 17; NMR spectrum, see Supplementary Figs. 77-82.

Compound **32**: white powder, $[\alpha]^{26}_D$ -80.5 (*c* 0.03, MeOH). HRMS *m/z* 427.2122 $[M + H]^+$ (*calc.* 427.2115 for $C_{25}H_{31}O_6$) (Supplementary Fig. 2) ^1H and ^{13}C NMR data, see Supplementary Table 18; NMR spectrum, see Supplementary Figs. 83-88.

Compound **33**: white powder, $[\alpha]^{26}_D$ -25.3 (*c* 0.11, MeOH). HRMS *m/z* 411.2171 $[M + H]^+$ (*calc.* 411.2166 for $C_{25}H_{31}O_5$) (Supplementary Fig. 2). ^1H and ^{13}C NMR data, see Supplementary Table 19; NMR spectrum, see Supplementary Figs. 89-94.

Supplementary Tables

Supplementary Table 1. Results of ^{18}O-labeling experiment.

	emervaridone C (**3**) (%)				
	m/z = 426 [M+NH$_4$]$^+$	*m/z* = 428 [M+NH$_4$]$^+$	*m/z* = 430 [M+NH$_4$]$^+$		
$H_2O/^{18}O_2$	2.2 ± 0.1	97.8 ± 0.1	–		
$H_2^{18}O/O_2$	95.6 ± 0.3	4.4 ± 0.3	–		
$H_2^{18}O\ /^{18}O_2$	1.3 ± 0.2	92.0 ± 0.8	6.8 ± 0.6		
$H_2^{18}O$ without enzyme	94.6 ± 0.2	5.4 ± 0.2	–		
	**4** (%)				
	m/z = 441 [M+H]$^+$	*m/z* = 443 [M+H]$^+$	*m/z* = 445 [M+H]$^+$	*m/z* = 447 [M+H]$^+$	*m/z* = 449 [M+H]$^+$
$H_2O/^{18}O_2$	–	1.1 ± 0.3	8.6 ± 0.8	90.3 ± 1.1	–
$H_2^{18}O/O_2$	95.0 ± 0.9	5.0 ± 0.9	–	–	–
$H_2^{18}O\ /^{18}O_2$	–	0.9 ± 0.1	4.8 ± 0.0	92.1 ± 0.3	2.2 ± 0.2
$H_2^{18}O$ without enzyme	100% ± 0	–	–	–	–
	**5** (%)				
	m/z = 441 [M+H]$^+$	*m/z* = 443 [M+H]$^+$	*m/z* = 445 [M+H]$^+$	*m/z* = 447 [M+H]$^+$	*m/z* = 449 [M+H]$^+$
$H_2O/^{18}O_2$	–	0.9 ± 0.2	3.4 ± 0.3	95.7 ± 0.4	–
$H_2^{18}O/O_2$	98.6 ± 0.3	1.4 ± 0.3	–	–	–
$H_2^{18}O\ /^{18}O_2$	–	0.8 ± 0.0	3.1 ± 0.2	95.1 ± 0.1	1.0 ± 0.0
$H_2^{18}O$ without enzyme	99.8 ± 0.1	0.2 ± 0.1	–	–	–

$H_2^{18}O$ without enzyme is incubating compound **3**, **4**, or **5** with 78% $H_2^{18}O$ without SptF, and other conditions are the same as labeling experiments except for the absence of enzyme. All reactions were performed in triplicate.

Supplementary Table 2. Conversion rate of unnatural substrates by SptF

Substrate	conversion rate (%)
**10**	51. 0 ± 1.8
**13**	30.8 ± 2.9
**15**	26.6 ± 0.4
**17**	5.6 ± 0.7
**19**	9.8 ± 1.8
**21**	35.8 ± 0.7
**24**	34.3 ± 3.0
**28**	52.6 ± 1.3

Reactions were performed on a 50-µL scale with 50 mM PIPES (pH 7.5), 0.2 mM $FeSO_4$, 5 mM α-ketoglutarate, 4 mM ascorbate, 100 µM of substrate, and 15 µM of wild-type or mutant SptF at 30 ºC over 24 hrs. All reactions were performed in triplicate.

Supplementary Table 3. Data collection and refinement statistics.

	SptF-apo	SptF-αKG/**1**	SptF Δ9	SptF N65T-NOG/**1**	SptF-NOG/**6**	SptF S114A-NOG/**15**
**Data collection**						
Space group	P1	P1	C2	P1	P1	P1
Cell dimensions						
a, *b*, *c* (Å)	49.6, 72.7, 79.0	49.4, 72.3, 77.6	90.2,52.3, 57.8	42.8, 50.6, 72.0	50.0, 72.6, 78.9	49.7, 72.3, 78.5
α, β, γ (°)	82.1, 84.9, 70.1	81.6, 85.6, 70.2	90, 99.4, 90	109.1, 91.3, 112.9	81.7, 84.2, 70.2	81.9, 84.9, 70.4
Resolution (Å)	48.6-2.1 (2.18-2.12)	47.7-1.95 (1.99-1.95)	45.1-1.40 (1.42-1.40)	44.6-2.05 (2.11-2.05)	48.4-2.1 (2.16-2.10)	48.2-2.1 (2.16-2.10)
R_{merge} (%)	9.8 (44.1)	8.5 (40.3)	3.6 (37.4)	17.6 (45.5)	13.8 (45.6)	6.5 (63.2)
$I / \sigma I$	10.4 (3.1)	7.8 (2.4)	12.4 (2.2)	7.1 (3.5)	5.7 (2.5)	7.3 (1.8)
Completeness (%)	97.6 (93.5)	97.0 (95.7)	99.7 (99.5)	96.2 (95.7)	94.3 (95.1)	97.6 (96.2)
Redundancy	5.4 (5.3)	3.6 (3.8)	3.8 (3.9)	4.2 (4.2)	3.3 (3.6)	2.1 (2.1)
**Refinement**						
Resolution (Å)	46.6-2.1	47.7-1.95	45.1-1.4	27.9-2.05	48.4-2.1	48.2-2.1
No. reflections	56648	70467	52217	31219	56650	57781
R_{work} / R_{free}	19.4/24.8	21.6/26.5	16.6/18.8	24.0/27.8	23.6/28.6	20.6/25.6
No. atoms						
Protein	8783	8602	2193	4303	8531	8465
Ligand/ion	4	40	11	41	44	75
Water	487	483	317	294	443	376
B-factors						
Protein	26.9	29.4	15.9	16.8	28.1	34.9
Ligand/ion	50.4	27.2	13.1	17.1	28.2	40.2
Water	29.8	30.8	27.8	20.1	28.9	36.0
R.m.s. deviations						
Bond lengths (Å)	0.002	0.004	0.006	0.003	0.002	0.002
Bond angles (°)	0.532	0.682	.951	0.634	0.595	0.567

*Data was collected from one crystal. *Values in parentheses are for highest-resolution shell.

Supplementary Table 4. Kinetic parameters measured in this study.

	Andiconin D (**1**)			Terretonin C (**15**)		
	k_{cat} (min^{-1})	K_M (μM)	k_{cat}/K_M (min^{-1}μM^{-1})	k_{cat} (min^{-1})	K_M (μM)	k_{cat}/K_M (min^{-1}μM^{-1})
SptF	9.85 ± 0.53	1.51 ± 0.31	6.52	0.18 ± 0.004	67.95 ± 4.13	0.003
N65A	4.10 ± 0.23	8.65 ± 1.73	0.47	0.14 ± 0.006	16.30 ± 2.65	0.008
I63A		N.D.		0.10 ± 0.003	17.04 ± 2.26	0.006
Del9		N.D.		0.12 ± 0.007	32.70 ± 6.34	0.004
F133A	0.25 ± 0.01	46.88 ± 6.31	0.005		N.D.	
	**3**			Andilesin C (**6**)		
	k_{cat} (min^{-1})	K_M (μM)	k_{cat}/K_M (min^{-1}μM^{-1})	k_{cat} (min^{-1})	K_M (μM)	k_{cat}/K_M (min^{-1}μM^{-1})
SptF	0.68 ± 0.10	77.07 ± 27.40	0.009	14.71 ± 0.22	1.15 ± 0.08	12.79
F133A		N.D.		0.21 ± 0.01	29.33 ± 4.79	0.007

Note: N.D. means not determined due to low activity. All reactions were performed in triplicate and plot for each measurement was shown as follows:

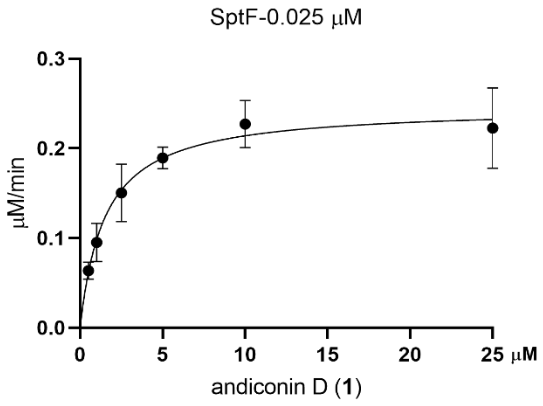

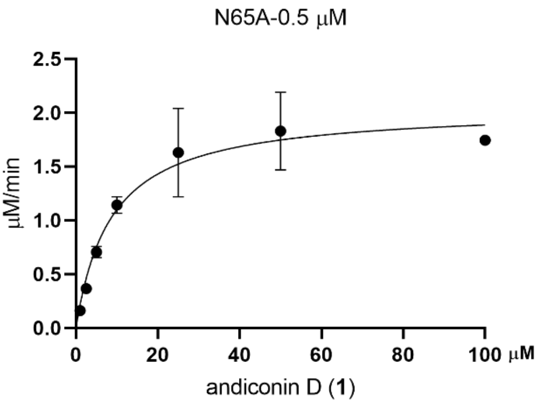

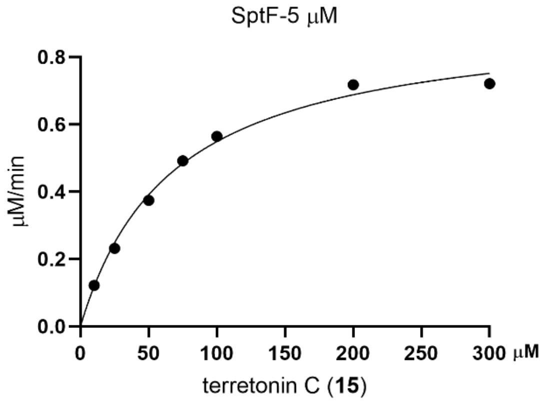

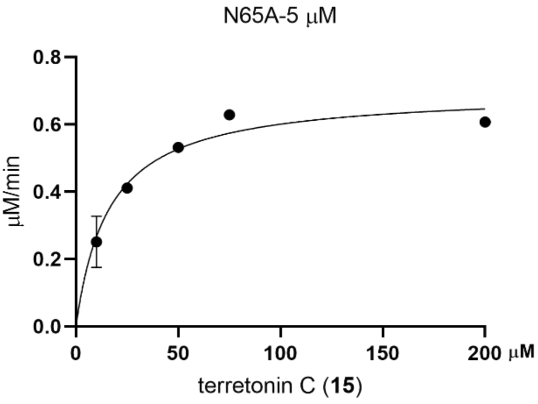

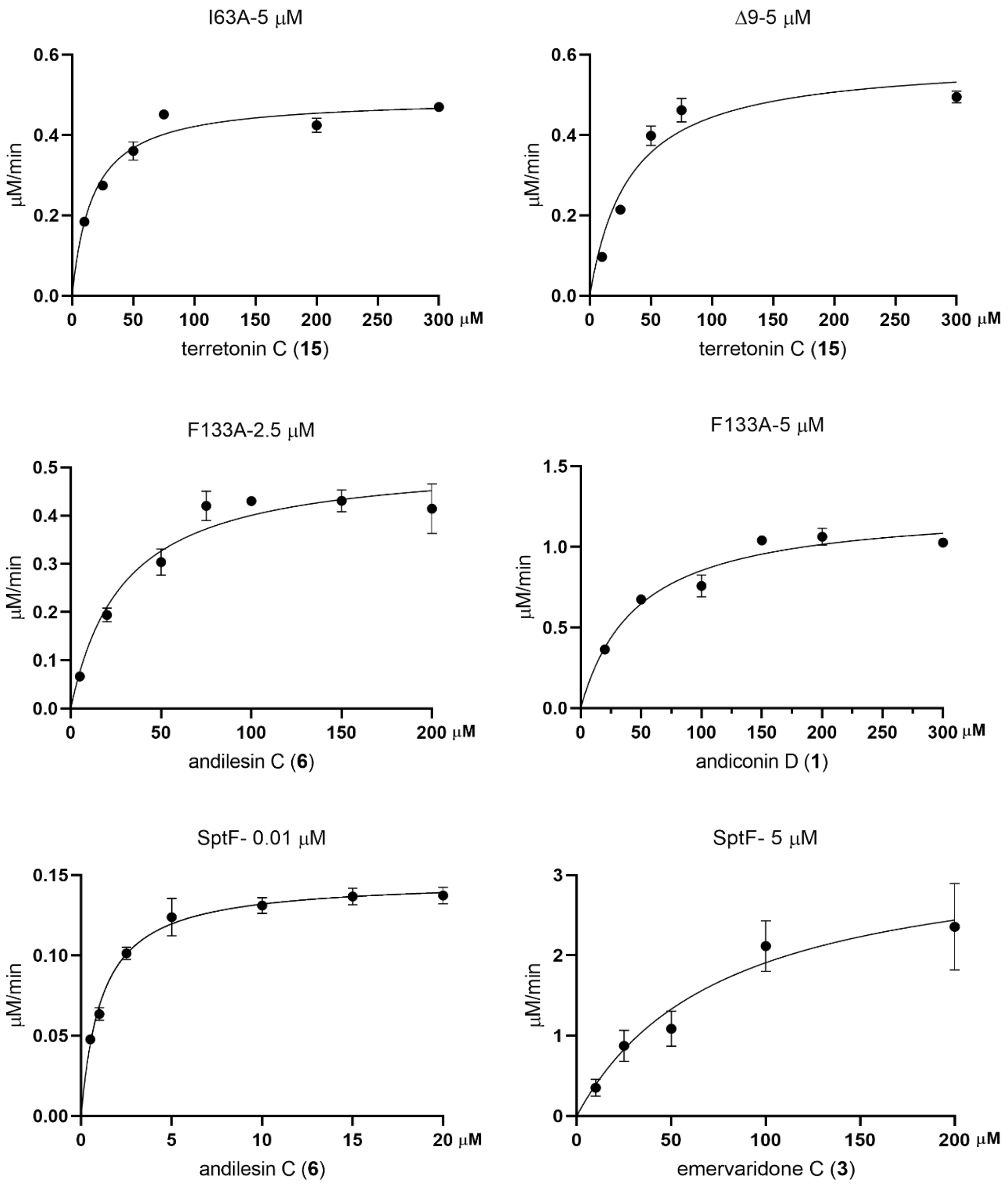
I63A-5 μM
μM/min
terretonin C (**15**)
Δ9-5 μM
μM/min
terretonin C (**15**)
F133A-2.5 μM
μM/min
andilesin C (**6**)
F133A-5 μM
μM/min
andiconin D (**1**)
SptF- 0.01 μM
μM/min
andilesin C (**6**)
SptF- 5 μM
μM/min
emervaridone C (**3**)

Supplementary Table 5. Crystal data and structure refinement for compounds **25**

Empirical formula	$C_{118.33}H_{125.04}Cl_{12}N_{24}O_{4.68}Zn_6$
Molecular weight	2775.94
Crystal system	monoclinic
Space group	*C*2
Lattice parameters: *a*, *b*, *c*, *β*	32.8346(8) Å, 14.4445(2) Å, 30.7569(8) Å, 100.966(2)°
Unit cell volume	14321.0(6) $Å^3$
Formula units per unit cell	4
R_{int}	5.85%
Number of parameters	2094
Number of restraints	1843
R_1	9.77%
wR_2	31.03%
GooF *S*	1.197
Flack parameters	0.122(10)
CCDC number	2090716

Supplementary Table 6. Substrates consumption by SptF truncation mutants.

Substrate	conversion rate (%)			
	SptF wild-type	Δ 6 (H61-V66)	Δ 9 (K58-V66)	Δ 16 (N54-K69)
**1**	100 ± 0.0	79.9 ± 0.5	93.6 ± 1.0	61.4 ± 3.2
**2**	100 ± 0.0	37.9 ± 1.3	57.6 ± 1.0	16.5 ± 0.9
**3**	100 ± 0.0	39.9 ± 0.9	55.7 ± 1.2	25.8 ± 4.8
**6**	100 ± 0.0	48.2 ± 1.5	74.6 ± 0.6	20.7 ± 0.3
**10**	90.7 ± 2.5	30.5 ± 5.5	25.8 ± 3.0	22.1 ± 2.4
**13**	39.3 ± 9.8	32.6 ± 7.1	22.3 ± 5.0	22.1 ± 4.1
**15**	47.0 ± 0.9	22.1 ± 1.0	25.9 ± 0.8	18.8 ± 1.5
**21**	94.4 ± 0.7	3.7 ± 0.6	3.9 ± 2.1	4.0 ± 1.8
**24**	83.5 ± 1.2	7.7 ± 2.4	16.4 ± 2.1	14.0 ± 3.5
**28**	90.4 ± 1.3	4.6 ± 0.1	5.4 ± 0.4	3.8 ± 0.2

Reactions were performed on a 50-µL scale with 50 mM PIPES (pH 7.5), 0.2 mM $FeSO_4$, 5 mM α-ketoglutarate, 4 mM ascorbate, 100 µM of substrate, and 40 µM of wild-type or mutant SptF at 30 ºC over 24 hrs. All reactions were performed in triplicate.

Supplementary Table 7. Primers used in this study.

Primers	Sequence (5′ to 3′)
SptF-N3-F	GGAGATATACATATGCCCCAACTTCATTATGTGCCTTA
SptF-N3-R	TGCTCGAGCATGCAAAAAAGCC
I63A-F	TACAACCACGACGCCAAAAATGTGG
I63A-R	CCACATTTTTGGCGTCGTGGTTGTA
N65T-F	CCACGACATCAAAACTGTGGGCTCGAAAACC
N65T-R	CGAGCCCACAGTTTTGATGTCGTGGTTG
N65A-F	CCACGACATCAAAGCTGTGGGCTCG
N65A-R	CGAGCCCACAGCTTTGATGTCGTGG
S114A-F	AACGGTGGCGCTATCCTCCATCTTG
S114A-R	CAAGATGGAGGATAGCGCCACCGTT
F133A-F	GGACCATGTGGCCTATCAAATCAGCAAGTGGCG
F133A-R	GCTGATTTGATAGGCCACATGGTCCTGGTGGATTG
F133Y-F	GGACCATGTGTACTATCAAATCAGCAAGTGGCG
F133Y-R	GCTGATTTGATAGTACACATGGTCCTGGTGGATTG
T148A-F	ATCCCGACCTTGCCATCAACTTCACGATGGCAC
T148A-R	GTGCCATCGTGAAGTTGATGGCAAGGTCGGGAT
T148S-F	ATCCCGACCTTAGCATCAACTTCACGATGGCAC
T148S-R	GTGCCATCGTGAAGTTGATGCTAAGGTCGGGAT
N150A-F	ACCTTACCATCGCCTTCACGATGGC
N150A-R	GCCATCGTGAAGGCGATGGTAAGGT
I231A-F	TTACCCCAGCCGAGAGTCACCACCACTTAC
I231A-R	TGGTGGTGACTCTCGGCTGGGGTAAAGTGAC
loop truncation (61-66)-F	AAAATACAACGGCTCGAAAACCAAGCAGCC
loop truncation (61-66)-R	TTCGAGCCGTTGTATTTTGGGCCGGGATTC
loop truncation (58-66)-F	CCGGCCCAGGCTCGAAAACCAAGCAGCC
loop truncation (58-66)-R	TTTTCGAGCCTGGGCCGGGATTCCAGTTGC
loop truncation (54-69)-F	CGCAACTGGACCAAGCAGCCATCCAACCTC
loop truncation (54-69)-R	CTGCTTGGTCCAGTTGCGAACGTAGTCGTC
loop truncation (53-71)-F	GTTCGCAACCAGCCATCCAACCTCAGCTT
loop truncation (53-71)-R	GGATGGCTGGTTGCGAACGTAGTCGTCAAC

Supplementary Table 8. NMR data of compound **4**.

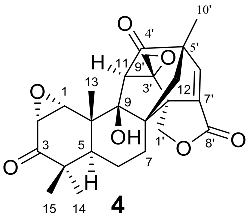

4

position	δ_H(mult., *J* in Hz)	δ_C, type	COSY	HMBC	NOESY
1	3.59 (d, 4.5)	64.3, CH	H-2	5	H-11, 13
2	3.33 (d, 4.5)	56.2, CH	H-1	3, 10, 12	
3		213.9, C			
4		45.2, C			
5	2.98 (dd, 13.5, 4.0)	40.1, CH	H-6α, 6β	6, 13	H-6α, 7α, 14
6α	1.72 (m)	21.2, CH_2	H-5, 7α, 7β		H-5, 7α, 14
6β	1.64 (qd, 13.5, 3.4)		H-5, 7α, 7β		H-7β, 15, 12'β
7α	2.13 (dt,12.3, 4.0)	29.9, CH_2	H-6α, 6β	8, 12	H-5, 6α, 1'β
7β	1.57 (dt, 12.3, 3.4)		H-6α, 6β	5, 9	H-6β, 1'β, 9'β
8		53.5, C			
9		87.5, C			
10		46.1, C			
11	3.10 (s)	71.8, CH		8, 9, 2', 3', 4', 5'	H-1, 13, 9'α
12α	2.87 (d, 14.5)	45.8, CH_2		9, 2', 5', 6', 10'	H-6β, 13, 10'
12β	1.32 (d, 14.5)			7, 8, 9, 4', 5', 6'	H-7β
13	1.03 (s)	19.6, CH_3		1, 4, 5, 9	H-1, 11, 12β, 10'
14	1.13 (s)	28.1, CH_3		3, 4, 5	H-5, 6α
15	1.04 (s)	21.1, CH_3		4, 14	H-6β
1'α	4.49 (d, 9.9)	69.2, CH_2		2', 3', 7', 8'	H-9'β
1'β	4.37 (d, 9.9)			8, 2', 3'	H-7α, 7β
2'		55.9, C			
3'		77.1, C			
4'		205.6, C			
5'		51.7, C			
6'	6.87 (s)	145.6, CH		2', 3', 5', 8' ,10'	H-1α, 10'
7'		141.5, C			
8'		169.8, C			
9'α	2.64 (d, 3.6)	47.8, CH_2			H-11
9'β	2.31 (d, 3.6)				H-1'α
10'	1.38 (s)	20.8, CH_3		12, 4', 5', 6'	

^{1}H 800 MHz, ^{13}C 200 MHz (CD_3OD)

Supplementary Table 9. NMR data of compound **5**.

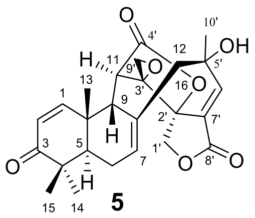

5

position	δ_H(mult., *J* in Hz)	δ_C, type	COSY	HMBC	NOESY
1	6.91 (d, 10.3)	159.5, CH	H-2	3, 5, 9	H-2, 9, 11, 13
2	6.01 (d, 10.3)	128.9, CH	H-1	10	H-1
3		206.5, C			
4		45.4, C			
5	1.35 (dd, 6.3, 11.0)	42.4 CH	H-6α, 6β	4, 6, 9, 13, 15	H-6α, 11, 14, 9'α
6α	2.20 (m)	26.1, CH_2	H-5, 7	5, 7, 8	H-5, 7, 14
6β	2.04 (m)		H-5, 7	7, 8	H-7, 13, 15
7	5.73 (m)	133.5, CH	H-6α, 6β	5, 9, 12	H-6α, 6β, 12α
8		131.5, C			
9	2.86 (s)	54.8, CH		5, 8, 11, 12, 13, 3', 4'	H-1, 12β, 13
10		42.9, C			
11	2.90 (s)	46.4, CH		8, 9, 10, 2', 3', 4', 9'	H-1, 5, 9'α
12α	2.65 (d, 14.9)	54.0, CH_2		7, 8, 9	H-7, 10'
12β	2.50 (m)			8, 5', 6'	H-9, 10'
13	1.16 (s)	20.4, CH_3		1, 9, 10	H-1, 6β, 9, 12β, 15
14	1.07 (s)	24.7, CH_3		3, 4, 5, 15	H-5, 6α, 9'α
15	1.05 (s)	21.7, CH_3		3, 4, 5	H-6β, 13
1'α	4.47 (d, 9.8)	71.4, CH_2		2', 3', 7', 8'	H-9'β
1'β	4.25 (d, 9.8)			2', 3'	
2'		84.1, C			
3'		68.3, C			
4'		178.1, C			
5'		74.6, C			
6'	7.04 (s)	153.1, CH		2', 5', 7', 8', 10'	H-12α, 10'
7'		127.3, C			
8'		170.4, C			
9'α	2.71 (d, 3.7)	53.4, CH_2			H-5, 11, 14
9'β	3.19 (d, 3.7)				H-1'α
10'	1.45 (s)	31.6, CH_3		8, 12, 5', 6', 7'	H-12α, 12β, 6'

^{1}H 800 MHz, ^{13}C 200 MHz (CD_3OD)

Supplementary Table 10. NMR data of compound **8**.

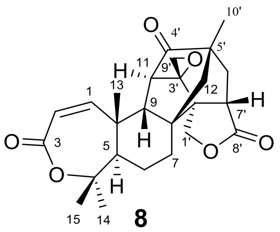

8

position	δ_H(mult., *J* in Hz)	δ_C, type	COSY	HMBC	NOESY
1	5.81 (d, 12.7)	147.5, CH	H-2	5, 9, 13	H-2, 9, 11, 13
2	5.96 (d, 12.7)	120.8, CH	H-1	3, 5	H-1
3		166.3, C			
4		83.4, C			
5	2.60 (m)	42.5, CH	H-6α, 6β	4, 6, 7, 9, 10, 13, 15	H-6α, 14, 1'α
6α	1.75 (m)	21.0, CH_2	H-5, 7α, 7β	10	H-5, 7α, 14, 1'β
6β	1.57 (m)		H-5, 7β		H-12β, 13
7α	2.05 (m)	25.7, CH_2	H-6α	8, 9	H-1'β
7β	1.87 (m)		H-6α, 6β		H-13
8		47.7, C			
9	1.79 (s)	63.0, CH		8, 10, 11, 12, 13, 2', 3'	H-1, 11, 12β, 13
10		42.5, C			
11	2.58 (s)	62.0, CH		8, 2', 3', 5'	H-1, 9, 9'α
12α	2.02 (d, 13.3)	50.4, CH_2		7, 8, 9, 4', 6'	H-7'
12β	1.41 (d, 13.3)			9, 6'	
13	1.34 (s)	26.4, CH_3		1, 9, 10	H-1, 6β, 7β, 9, 15
14	1.41 (s)	30.8, CH_3		4, 14	H-5, 6α, 1'α
15	1.50 (s)	23.3, CH_3		4, 5, 15	H-12β, 13
1'α	4.69 (d, 8.5)	70.8, CH_2		2', 3', 7', 8'	H-5, 6α, 14
1'β	4.24 (d, 8.5)			2'	H-6α, 7α
2'		64.5, C			
3'		52.8, C			
4'		208.8, C			
5'		44.9, C			
6'α	1.83 (m)	29.5, CH_2	H-7'	4', 5', 7', 10'	H-10'
6'β	1.25 (m)				
7'	3.04 (dd, 10.7, 8.5)	41.6, CH	H-6'α	2', 3', 6', 8'	H-12α, 6'α, 1'β
8'		174.4, C			
9'α	2.83 (d, 3.0)	48.1, CH_2			H-1'α, 6'α
9'β	2.80 (d, 3.0)				H-11, 6'α
10'	1.16 (s)	23.7, CH_3		12, 4', 5', 6'	H-12α, 12β

^{1}H 800 MHz, ^{13}C 200 MHz ($CDCl_3$)

Supplementary Table 11. NMR data of compound **9**.

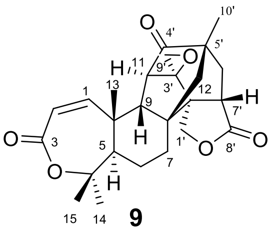

9

position	δ_H(mult., *J* in Hz)	δ_C, type	COSY	HMBC	NOESY
1	5.94 (d, 12.7)	148.3, CH	H-2	2, 5	H-2, 11, 13
2	6.02(d, 12.7)	120.4, CH	H-1	1, 10	H-1
3		166.0, C			
4		83.8, C			
5	2.29 (m)	45.1, CH	H-6α, 6β	4, 6, 7, 9, 10, 15	H-6α, 14, 1'α
6α	1.77 (m)	21.2, CH_2	H-5, 7β	7, 10	H-7α, 14
6β	1.59 (m)		H-5		H-6'β
7α	1.99 (m)	26.2, CH_2		9, 12	H-1'β
7β	1.81 (m)		H-6α		H-13
8		44.9, C			
9	1.71 (s)	61.2, CH		5, 7, 10, 12, 4'	H-12β, 13
10		42.8, C			
11	2.56 (s)	60.1, CH		10, 2', 3', 4', 9'	H-1, 9, 9'β
12α	2.04 (d, 13.3)	50.3, CH_2		9, 13, 4', 5', 6'	H-7'
12β	1.41 (d, 13.3)			9, 13, 5', 6'	
13	1.35 (s)	26.1, CH_3		1, 5, 9, 10	H-6β, 7β, 9, 15
14	1.41 (s)	30.9, CH_3		4, 5, 15	H-5, 6α
15	1.51 (s)	23.2, CH_3		4, 5, 14	H-13
1'α	4.37 (d, 9.3)	73.6, CH_2		2', 3', 7', 8'	H-5, 9'α
1'β	4.29 (d, 9.3)			2'	H-7α, 7'
2'		67.0, C			
3'		50.6, C			
4'		208.2, C			
5'		45.1, C			
6'α	2.21 (m)	29.9, CH_2	H-7'	12, 4', 7', 8'	
6'β	1.80 (m)		H-7'	4', 5', 7', 10'	H-7', 10'
7'	2.90 (dd, 10.7, 8.1)	41.9, CH	H-6'α, 6'β	2', 3', 6', 8'	H-12α, 6'β, 1'β
8'		173.5, C			
9'α	3.13 (d, 4.2)	53.2, CH_2		2'	H-1'α
9'β	2.80 (d, 4.2)			11, 2'	H-11
10'	1.19 (s)	24.1, CH_3		12, 4', 5', 6'	H-12α, 12β, 6'β

^{1}H 900 MHz, ^{13}C 225 MHz ($CDCl_3$)

Supplementary Table 12. NMR data of compound **11**.

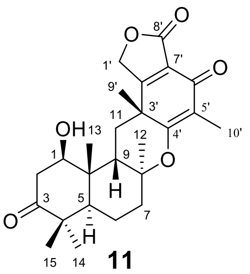

11

position	δ_H(mult., *J* in Hz)	δ_C, type	COSY	HMBC	NOESY
1	4.26 (d, 9.4)	70.0, CH	H-2β	3	H-2β, 5, 11α, 12
2α	3.19 (dd, 16.3, 9.4)	46.4, CH_2		1, 3	H-1, 5, 14
2β	2.17 (d, 16.3)		H-1	1, 3	
3		215.6, C			
4		46.4, C			
5	1.72 (m)	44.3, CH	H-6β	1, 13, 14	H-1, 2α, 12, 14
6α	1.66 (m)	17.7, CH_2	H-7α	8	
6β	1.76 (m)		H-5, 7α, 7β		
7α	1.99 (m)	37.9, CH_2	H-6α, 6β		H-6α, 12
7β	2.30 (dd, 14.0, 8.3)		H-6β	8	H-6β
8		85.2, C			
9	2.23 (dd, 13.3, 2.6)	44.1, CH	H-11α, 11β		H-6β, 13, 9'
10		41.3, C			
11α	1.92 (t, 13.3)	31.8, CH_2	H-9	9'	H-1, 12
11β	2.08 (dd, 13.3, 2.6)		H-9		H-13, 9'
12	1.35 (s)	75.7, CH_3		7, 8, 9	H-1, 5, 7α, 11α, 10'
13	0.94 (s)	16.3, CH_3		1, 5, 9	
14	1.08 (s)	29.5, CH_3		3, 5, 15	H-2α, H-5, 6α
15	1.12 (s)	19.7, CH_3		3, 4, 14, 9'	H-6β, 13
1'α	4.97 (d, 18.3)	67.1, CH_2		6'	
1'β	4.93 (d, 18.3)			6'	H-9'
2'		121.1, C			
3'		39.9, C			
4'		169.6, C			
5'		122.8, C			
6'		180.4, C			
7'		167.9, C			
8'		161.2, C			
9'	1.52 (s)	26.9, CH_3		3', 4', 11	H-9, 11β, 1'β
10'	1.83 (s)	8.2, CH_3		4', 5', 6'	H-12

^{1}H 900 MHz, ^{13}C 225 MHz ($CDCl_3$)

Supplementary Table 13. NMR data of compound **14**.

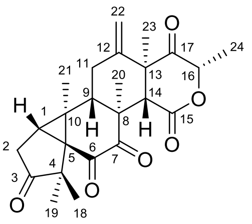

14

position	δ_H(mult., *J* in Hz)	δ_C, type	COSY	HMBC	NOESY
1	2.22 (d, 6.2)	38.7, CH	H-2β	2, 3, 5, 6, 9	H-2β, 9
2α	2.44 (d, 18.8)	36.5, CH_2		3, 4, 5, 10	H-21, 22b
2β	2.94 (dd, 18.8, 6.2)		H-1	1, 3, 10	H-1, 18
3		215.9, C			
4		51.9, C			
5		50.7, C			
6		199.5, C			
7		201.1, C			
8		52.0, C			
9	1.67 (dd, 13.1, 3.8)	54.3, CH	H-11α, 11β		H-1, 11β, 14
10		37.3, C			
11α	2.79 (t, 13.1)	32.6, CH_2	H-9	8, 9, 12, 22	H-20, 21, 23
11β	2.54 (dd, 13.7, 3.8)		H-9	8, 9, 13	H-9, 22b
12		142.3, C			
13		49.3, C			
14	3.34 (s)	47.9, CH		7, 8, 12, 13, 15, 17, 20, 23	H-9, 16
15		167.9, C			
16	5.01 (q, 6.5)	77.8, CH	H-24	24	H-14, 24
17		206.4, C			
18	1.56 (s)	26.6, CH_3		3, 4, 5, 19	H-2β
19	1.35 (s)	19.2, CH_3		3, 4, 5, 18	H-20, 21
20	1.79 (s)	11.9, CH_3		7, 8, 9, 14	H-11α, 21, 23
21	1.16 (s)	12.7, CH_3		1, 5, 9, 10	H-2α, 11α, 20
22a	5.15 (brd)	113.2, CH_2		11, 13	H-22b
22b	5.04 (brd)			11, 13	H-11β, 22a
23	1.41 (s)	22.7, CH_3		12, 13, 14, 17	H-11α, 20
24	1.51 (d, 6.5)	14.8, CH_3	H-16	16, 17	H-16

^{1}H 800 MHz, ^{13}C 200 MHz ($CDCl_3$)

Supplementary Table 14. NMR data of compound **16**.

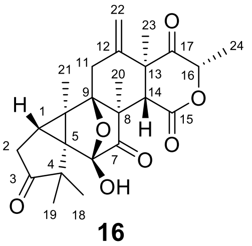

16

position	δ_H(mult., *J* in Hz)	δ_C, type	COSY	HMBC	NOESY
1	2.33 (d, 6.7)	16.7, CH	H-2β	2, 3, 5, 6, 9	H-2β, 11β
2α	2.19 (d, 19.2)	35.2, CH_2		1, 3, 4, 5, 10	H-21
2β	2.83 (dd, 19.2, 6.7)		H-1	1, 3, 10	
3		218.0, C			
4		46.1, C			
5		38.7, C			
6		104.2, C			
7		208.8, C			
8		52.4, C			
9		83.1, C			
10		33.4, C			
11α	2.93 (dt, 15.4, 1.3)	32.0, CH_2		9, 10, 12, 13, 22	H-21, 23
11β	2.45 (d, 15.4)			8, 9, 10, 12, 13, 22	H-22a
12		139.5, C			
13		50.8, C			
14	3.61 (s)	47.5, CH		7, 8, 12, 13, 15, 17, 20, 23	H-16
15		167.8, C			
16	4.94 (q, 6.3)	77.0, CH	H-24	24	H-14
17		205.0, C			
18	1.27 (s)	24.6, CH_3		3, 4, 5, 19	H-2β
19	1.02 (s)	20.0, CH_3		3, 4, 5, 18	H-20, 21
20	1.78 (s)	15.6, CH_3		7, 8, 9, 14	H-19, 21, 24
21	1.14 (s)	8.9, CH_3		1, 5, 9, 10	H-2α, 11α, 20
22a	5.06 (d, 1.3)	114.7, CH_2		11, 13	H-11β
22b	4.90 (d, 1.3)			11, 12, 13	H-24
23	1.45 (s)	21.2, CH_3		12, 13, 14, 17	H-11α, 20
24	1.47 (d, 6.3)	14.4, CH_3	H-16		H-16, 22b
OH	4.16 (s)			5	

^{1}H 900 MHz, ^{13}C 225 MHz ($CDCl_3$)

Supplementary Table 15. NMR data of compound **22**.

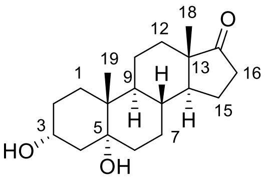

22

position	δ_H(mult., *J* in Hz)	δ_C, type	COSY	HMBC	NOESY
1α	1.71 (m)	27.8, CH_2	H-2β	2, 3, 10, 19	H-9
1β	1.29 (m)		H-2α, 2β	2, 3, 5, 10, 19	H-2β, 19
2α	1.73 (m)	29.6, CH_2	H-1β, 3	3, 4, 10	H-3
2β	1.83 (m)		H-1α, 1β, 3	1, 10	H-1β
3	4.06 (m)	68.6, CH	H-2α, 2β, 4β	1, 5	H-2β, 4β
4α	1.84 (dd, 9.6, 3.1)	39.9, CH_2	H-3	5, 10	H-2α, 6α
4β	1.56 (m)		H-3	2, 3, 5, 6, 10	H-3, 11β, 12β
5		76.1, C			
6α	1.64 (m)	34.6, CH_2	H-7α, 7β	4, 7, 8	H-4α, 7α
6β	1.34 (m)		H-7β	5, 8, 10	H-4β, 7β
7α	1.53 (m)	25.5, CH_2	H-6α, 8	9	H-6α
7β	1.44 (m)		H-6α, 6β, 8	6, 8, 9, 14	H-6β, 15β
8	1.67 (m)	35.7, CH	H-7α, 7β, 9, 14	7, 9, 14	H-18, 19
9	1.60 (m)	47.0, CH	H-8, 11α, 11β	8, 10, 11, 19	H-1α, 14
10		41.0, C			
11α	1.58 (m)	21.3, CH_2	H-9, 12α, 12β	10, 12	
11β	1.33 (m)		H-9, 12α, 12β	10, 12	H-12β, 18, 19
12α	1.26 (ddd, 14.1, 12.8, 3.9)	33.0, CH_2	H-11α, 11β	11, 13, 17, 18	H-11α, 14
12β	1.77 (ddd, 12.8, 3.9, 2.6)		H-11α, 11β	9, 11, 13, 14	H-12β, 11β, 18
13		49.2, C			
14	1.39 (m)	52.6, CH	H-8, 15α	7, 8, 9, 11,12,13,15,18	H-9, 12α, 15α
15α	1.95 (m)	22.6, CH_2	H-14, 16α	13, 14, 17	H-14
15β	1.55 (m)		H-16β	14	H-16β, 18
16α	2.07 (ddd, 19.4, 9.6, 8.9)	36.7, CH_2	H-15α, 15β	15, 17	
16β	2.43 (dd, 19.4, 8.9)		H-15β	14, 15, 17	H-15β
17		224.1, C			
18	0.88 (s)	14.3, CH_3		12, 13,14, 17	H-8, 11β, 12β, 15β, 19
19	1.01 (s)	16.2, CH_3		1, 5, 9, 10	H-1β, 8, 11β, 18

^{1}H 800 MHz, ^{13}C 200 MHz (CD_3OD)

Supplementary Table 16. NMR data of compound **23**.

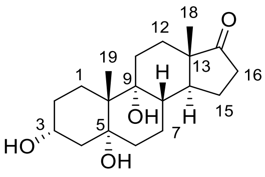

23

position	δ_H(mult., *J* in Hz)	δ_C, type	COSY	HMBC	NOESY
1α	1.27 (m)	22.8, CH_2	H-2α, 2β		H-11α
1β	2.31 (ddd,14.5, 13.6, 4.6)		H-2α, 2β	2	H-2β, 15β
2α	1.87 (m)	29.7, CH_2	H-1α, 1β, 3	1	
2β	1.82 (m)		H-1α, 1β, 3	10	H-3
3	4.04 (m)	68.4, CH	H-2α, 2β, 4α, 4β	1, 5	H-2β, 15β
4α	1.51 (m)	40.4, CH_2	H-3	2, 3, 5, 6, 10	H-6α
4β	1.91 (dd, 14.5, 3.4)		H-3	5	H-3, 19
5		79.4, C			
6α	1.41 (m)	35.3, CH_2	H-7	4, 5, 8, 10	
6β	1.68 (m)		H-7	5	H-8, 18, 19
7	1.65 (m)	27.5, CH_2	H-6α, 6β, 8	5, 8	
8	2.04 (m)	39.0, CH	H-7, 14	14	H-11β, 15β, 18, 19,
9		79.6, C			
10		43.6, C			
11α	1.65 (m)	20.9, CH_2	H-12		H-1α
11β	1.43 (m)		H-12		H-8
12	1.50 (m)	28.2, CH_2	H-11α, 11β	13	
13		49.0, C			
14	2.03 (m)	45.3, CH	H-8, 15β	13	H-15α
15α	1.57 (m)	22.5, CH_2	H-16α, 16β	16	H-14
15β	1.89 (m)		H-14, 16β	14, 17	H-3, 8, 16β
16α	2.43 (ddd, 19.4, 9.0, 0.5)	36.9, CH_2	H-15α	14, 15, 17	H-15α
16β	2.09 (ddd,19.4, 9.6, 9.0)		H-15α, 15β	14, 15, 17	H-15β
17		223.8, C			
18	0.89 (s)	13.4, CH_3		12, 13, 14, 17	H-6β, 8
19	1.11 (s)	19.2, CH_3		1, 5, 9, 10	H-4β, 6β, 8

^{1}H 800 MHz, ^{13}C 200 MHz (CD_3OD)

Supplementary Table 17. NMR data of compound **25**.

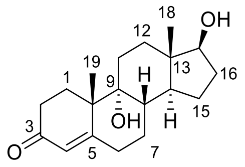

25

position	δ_H(mult., *J* in Hz)	δ_C, type	COSY	HMBC
1	2.34 (m)	29.7, CH_2		2, 3, 10, 19
	1.66 (m)		H-2	2, 3, 5, 19
2	2.41 (m)	35.0, CH_2	H-1	1, 3, 4, 10
	2.24 (m)			4, 5
3		202.5, C		
4	5.7 (s)	126.5, CH		2, 6, 10
5		174.6, C		
6	2.48 (m)	33.3, CH_2		4, 5
	2.22 (m)		H-7	5, 8
7	1.48 (m)	24.3, CH_2	H-6, 8	5
	1.25 (m)			8, 14
8	1.92 (m)	39.1, CH	H-7, 14	7, 10, 14, 15
9		78.0, C		
10		46.1, C		
11	1.73 (m)	27.6, CH_2	H-12	10, 12, 13
	1.46 (m)		H-12	8, 9, 12
12	1.58 (m)	33.3, CH_2	H-11	9, 11, 13, 14
	1.33 (m)		H-11	10, 13, 18
13		44.0, C		
14	1.47 (m)	45.2, CH	H-8, 15	7, 18
15	1.49 (m)	26.0, CH_2	H-14, 16	13
	1.44 (m)		H-14, 16	8
16	1.93 (m)	30.8, CH_2	H-15, 17	13, 14, 15, 17
	1.41 (m)		H-15, 17	17, 19
17	3.55 (m)	82.3, CH	H-16	13, 16, 18
18	0.72 (s)	10.9, CH_3		14, 17
19	1.28 (s)	20.5, CH_3		1, 5, 9, 10

^{1}H 500 MHz, ^{13}C 125 MHz (CD_3OD)

Supplementary Table 18. NMR data of compound **26/27**.

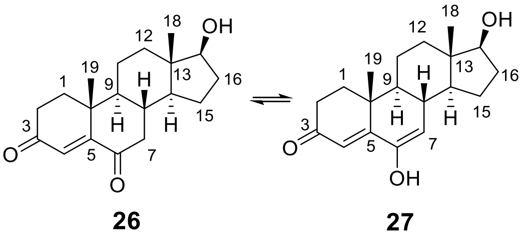

position	δH(mult., *J* in Hz)		δC, type		COSY	HMBC	NOESY
1α	1.94[a] (m)	1.73[b] (m)	36.7[a], CH_2	35.5[b], CH_2	H-2α, 2β	2, 9, 10, 19	H-2α, 7α
1β	2.20[a] (m)	2.06[b] (m)			H-2α, 2β	2, 3, 5, 10, 19	H-11β, 19
2α	2.62[a] (m)	2.62[b] (m)	34.9[a], CH_2	34.7[b], CH_2	H-1α, 1β	1, 3	H-1α, 14, 12α
2β	2.40[a] (m)	2.40[b] (m)			H-1α, 1β	3, 10	H-1β
3			202.0[a], C	202.9[b], C			
4	6.06[a] (s)	6.16[b] (s)	126.1[a], CH	119.7[b], CH		2, 5, 6, 10	
5			163.6[a], C	163.4[b], C			
6			203.9[a], C	148.4[b], C			
7α	2.61[a] (m)	5.32[b] (s)	47.2[a], CH_2	113.6[b], CH	H-8	8, 9	H-1α, 14
7β	2.13[a] (dd, 12.8, 16.1)				H-8	6, 8, 14	
8	1.99[a] (m)	2.29[b] (m)	35.6[a], CH	37.1[b], CH	H-7α, 7β		H-18, 19
9	1.47[a] (m)	1.16[b] (m)	52.3[a], CH	53.2[b], CH	H-8, 11α, 11β	11	
10			41.2[a], C	37.9[b], C			
11α	1.55[a] (m)	1.46[b] (m)	21.8[a], CH_2	21.5[b], CH_2	H-9, 12α, 12β	9, 12	H-12α
11β	1.74[a] (m)	1.61[b] (m)			H-9, 12α, 12β		H-1β, 19
12α	1.20[a] (m)	1.89[b] (m)	37.5[a], CH_2	37.9[b], CH_2	H-11α, 11β		H-2α, 11α
12β	1.93[a] (m)	1.13[b] (m)			H-11α, 11β		H-18
13			44.3[a], C	45.0[b], C			
14	1.21[a] (m)	1.15[b] (m)	52.5[a], CH	50.6[b], CH	H-15α	9	H-2α, 7α
15α	1.64[a] (m)	1.75[b] (m)	24.1[a], CH_2	24.1[b], CH_2	H-14	13	H-16α
15β	1.34[a] (m)	1.43[b] (m)			H-16α	8, 14, 16	H-18
16α	2.03[a] (m)	2.03[b] (m)	30.7[a], CH_2	30.7[b], CH_2	H-15β, 17	13, 15, 17	H-15α, 17
16β	1.52[a] (m)	1.52[b] (m)			H-17		H-15β
17	3.63[a] (m)	3.63[b] (m)	82.2[a], CH	82.2[b], CH	H-16α, 16β	12, 16, 18	H-11α, 14, 16α
18	0.80[a] (s)	0.83[b] (s)	11.6[a], CH_3	11.7[b], CH_3		12, 13, 14, 17	H-8, 12β, 15β, 19
19	1.20[a] (s)	1.16[b] (s)	17.8[a], CH_3	17.0[b], CH_3		1, 5, 9, 10	H-1β, 8, 11β, 18

note: **26** is interchangeable with **27**. a. assignments for **26**; b. assignments for **27**

^{1}H 800 MHz, ^{13}C 200 MHz (CD_3OD)

Supplementary Table 19. NMR data of compound **32**.

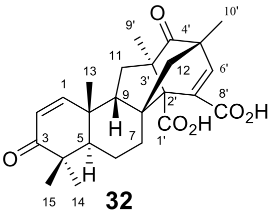

32

position	δ_H(mult., *J* in Hz)	δ_C, type	COSY	HMBC	NOESY
1	6.58 (d, 10.1)	158.4, CH	H-2	3, 5, 9, 10	H-2, 9, 11β, 13
2	5.80 (d, 10.1)	125.6, CH	H-1	4, 10	H-1
3		207.5, C			
4		45.5, C			
5	2.14 (m)	38.8, CH	H-6α, 6β	4, 6, 9, 10, 13, 15	H-11α, 14
6α	1.87 (m)	19.2, CH_2	H-5, 7α, 7β		
6β	1.60 (m)		H-5, 7β		H-7β, 13, 15, 10'
7α	2.26 (dd, 10.1, 14.8)	28.9, CH_2	H-6α	5, 8, 12	
7β	1.79 (m)		H-6β	6, 8, 12, 2'	H-6β, 12α, 13
8		52.4, C			
9	1.71 (dd, 8.5, 10.9)	59.8, CH	H-11α, 11β	5, 8, 10, 11, 12, 13	H-1, 11β, 12α, 13
10		40.3, C			
11α	1.99 (t, 11.9)	37.0, CH_2	H-9	9, 10, 3', 4'	H-5
11β	1.52 (dd, 8.5, 11.9)		H-9	8, 2'	H-1, 9
12α	1.56 (d, 11.5)	59.5, CH_2		9, 5', 6'	H-7β, 9, 13, 10'
12β	1.54 (d, 11.5)			7, 9, 4', 5'	
13	1.29 (s)	23.1 , CH_3		1, 5, 9, 10	H-6β, 7β, 9, 15
14	1.10 (s)	23.9, CH_3		3, 4, 5, 15	H-5, 6α
15	1.02 (s)	21.8, CH_3		3, 4, 5, 14	H-6β, 13
1'		ND*			
2'		64.2, C			
3'		56.6, C			
4'		218.8, C			
5'		50.1, C			
6'	6.60 (brs)	140.8, CH		2', 5'	H-10'
7'		136.3, C			
8'		ND*			
9'	1.22 (s)	16.8, CH_3		11, 2', 3', 4'	H-11α
10'	1.20 (s)	17.9, CH_3		12, 6'	H-12α, 6'

* ND: undetected quaternary carbon.

^{1}H 800 MHz, ^{13}C 200 MHz (CD_3OD)

Supplementary Table 20. NMR data of compound **33**.

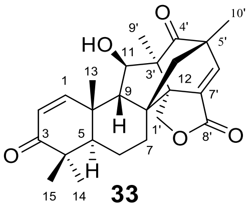

33

position	δ_H(mult., *J* in Hz)	δ_C, type	COSY	HMBC	NOESY
1	6.89 (d, 10.3)	154.5, CH	H-2	3, 5, 9	H-2, 9, 11, 13, OH
2	5.96 (d, 10.3)	125.4, CH	H-1	4, 10	H-1
3		203.5, C			
4		44.4, C			
5	1.35 (m)	40.6, CH	H-6		H-6, 11, 14, 1'α, 1'β
6	1.81 (m)	18.2, CH_2	H-5, 7α, 7β	5, 7, 10	H-7α, 13, 14, 15, 1'β
7α	1.65 (m)	27.8, CH_2	H-6	8, 2'	H-6, 12α, 1'β
7β	1.86 (m)		H-6	6, 8, 2'	H-12α, 13
8		43.9, C			
9	1.45 (d, 8.5)	66.3, CH	H-11	5, 8, 10, 11, 12, 13	H-1, 12α, 12β, 13
10		38.7, C			
11	3.37 (t, 8.5)	75.8, CH	H-9, OH	9, 10, 3', 4', 9'	H-5, 9, 1'α, 9'
12α	1.50 (d, 12.3)	58.4, CH_2		7, 8, 9, 4', 5', 6'	H-7β, 9, 10'
12β	1.72 (d, 12.3)			9, 2', 5', 6', 10'	H-7β, 9, 10'
13	1.31 (s)	23.0, CH_3		1, 5, 9, 10	H-1, 6, 7β, 9, 12β, 15
14	1.15 (s)	23.7, CH_3		3, 4, 5, 15	H-5, 6, 1'β
15	1.08 (s)	21.4, CH_3		3, 4, 5, 14	H-6, 13
1'α	4.37 (d, 10.5)	68.5, CH_2		8, 2', 3', 8'	H-5, 11, 9'
1'β	4.47 (d, 10.5)			8, 2', 3', 8'	H-5, 6, 7α
2'		58.2, C			
3'		54.8, C			
4'		215.4, C			
5'		52.4, C			
6'	7.18 (s)	143.3, CH		2', 4', 5', 8', 10'	H-12α, 10'
7'		133.7, C			
8'		166.6, C			
9'	1.07 (s)	15.0, CH_3		11, 2', 3', 4'	H-11, 1'α, OH,
10'	1.38 (s)	16.8, CH_3		12, 4', 5', 6'	H-5, 9, 12α, 6'
OH	2.82 (d, 9.0)		H-11	11, 3'	H-1, 9'

^{1}H 900 MHz, ^{13}C 225 MHz ($CDCl_3$)

Supplementary Figures

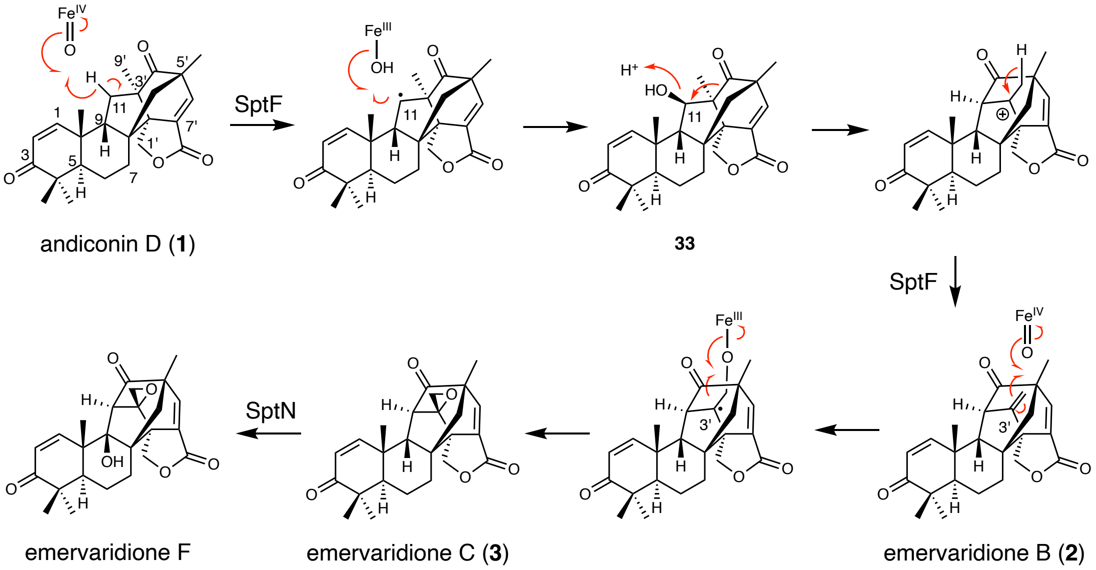

Supplementary Fig. 1 | Proposed pathway for generation of emervaridone B (3) and emeridone F from andiconin D (1) in previous studies[1]. In the proposed pathway, the reaction starts with abstraction of hydrogen at C11 of **1**, and subsequent hydroxyl group rebound at C11 yields **33** as a putative on-pathway intermediate. Then the elimination of hydroxyl group at C11 induces the 1,2-acyl shift. The resultant cationic species undergoes the deprotonation from C-9′ to form emervaridone B (**2**), which undergoes epoxidation to form **3**. Another αKG-dependent dioxygenase SptN catalyzes C-9 hydroxylation on **3** to generate emeridone F

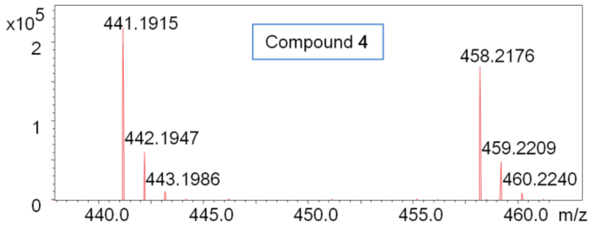
x10^5
Compound 4
441.1915
442.1947
443.1986
458.2176
459.2209
460.2240
440.0 445.0 450.0 455.0 460.0 m/z

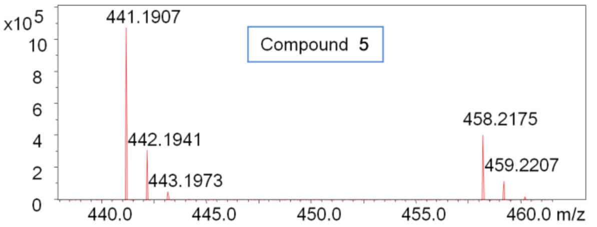
x10^5
Compound 5
441.1907
442.1941
443.1973
458.2175
459.2207
440.0 445.0 450.0 455.0 460.0 m/z

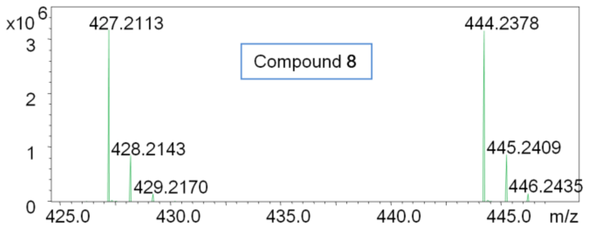
x10^6
Compound 8
427.2113
428.2143
429.2170
444.2378
445.2409
446.2435
425.0 430.0 435.0 440.0 445.0 m/z

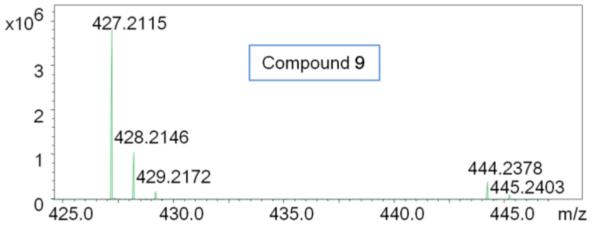
x10^6
Compound 9
427.2115
428.2146
429.2172
444.2378
445.2403
425.0 430.0 435.0 440.0 445.0 m/z

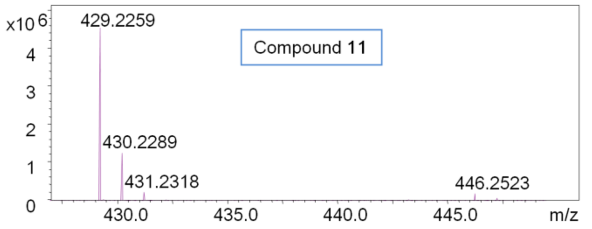
x10^6
Compound 11
429.2259
430.2289
431.2318
446.2523
430.0 435.0 440.0 445.0 m/z

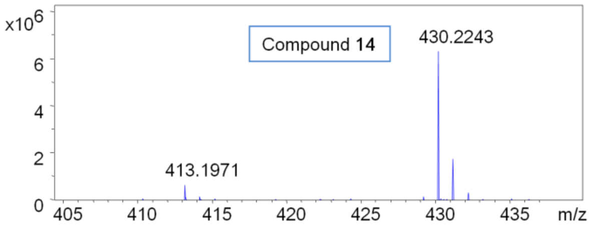
x10^6
Compound 14
413.1971
430.2243
405 410 415 420 425 430 435 m/z

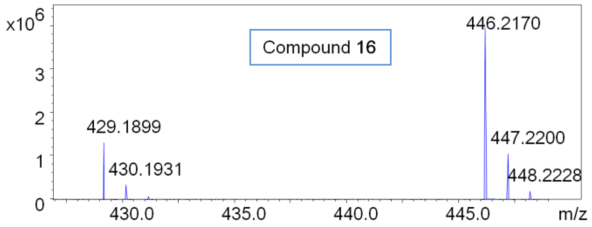
x10^6
Compound 16
429.1899
430.1931
446.2170
447.2200
448.2228
430.0 435.0 440.0 445.0 m/z

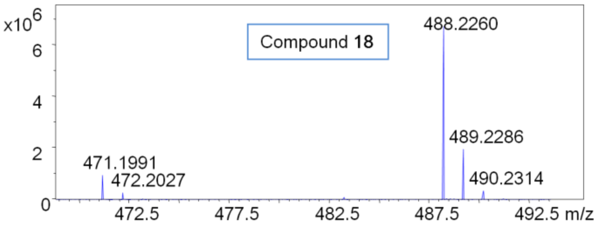
x10^6
Compound 18
471.1991
472.2027
488.2260
489.2286
490.2314
472.5 477.5 482.5 487.5 492.5 m/z

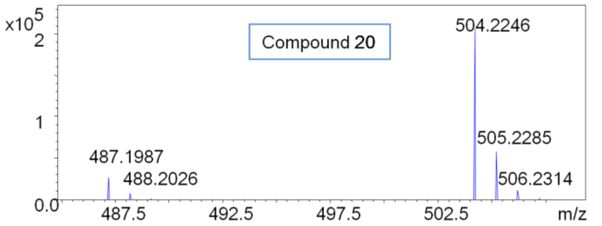
x10^5
Compound 20
487.1987
488.2026
504.2246
505.2285
506.2314
487.5 492.5 497.5 502.5 m/z

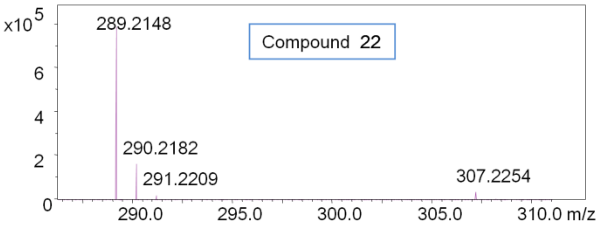
x10^5
Compound 22
289.2148
290.2182
291.2209
307.2254
290.0 295.0 300.0 305.0 310.0 m/z

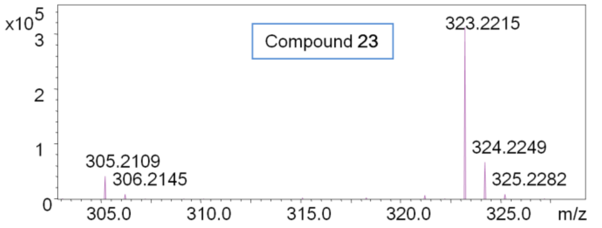
x10^5
Compound 23
305.2109
306.2145
323.2215
324.2249
325.2282
305.0 310.0 315.0 320.0 325.0 m/z

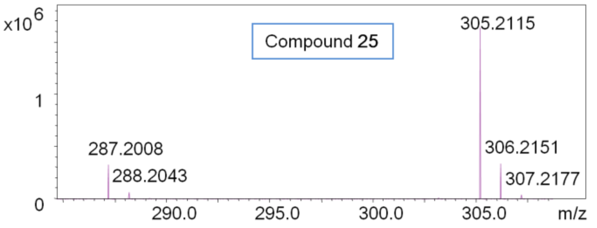
x10^6
Compound 25
287.2008
288.2043
305.2115
306.2151
307.2177
290.0 295.0 300.0 305.0 m/z

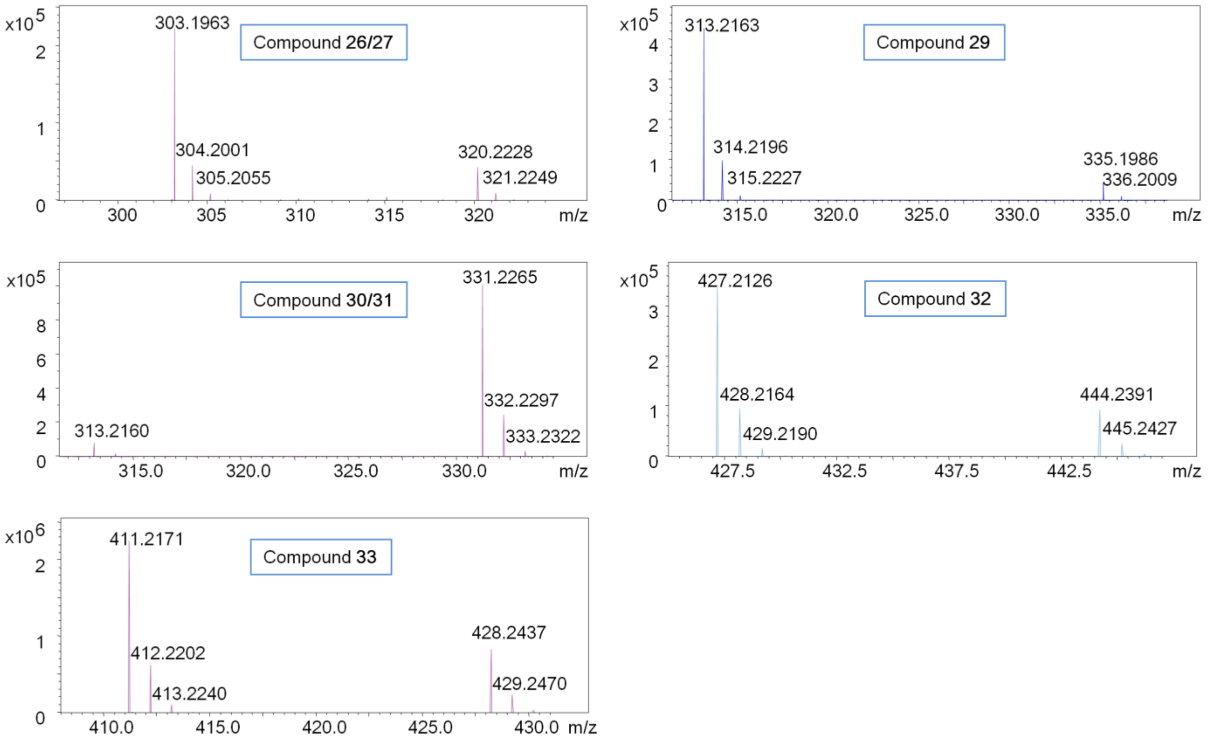

Supplementary Fig. 2 | HRMS spectrum of key compounds.

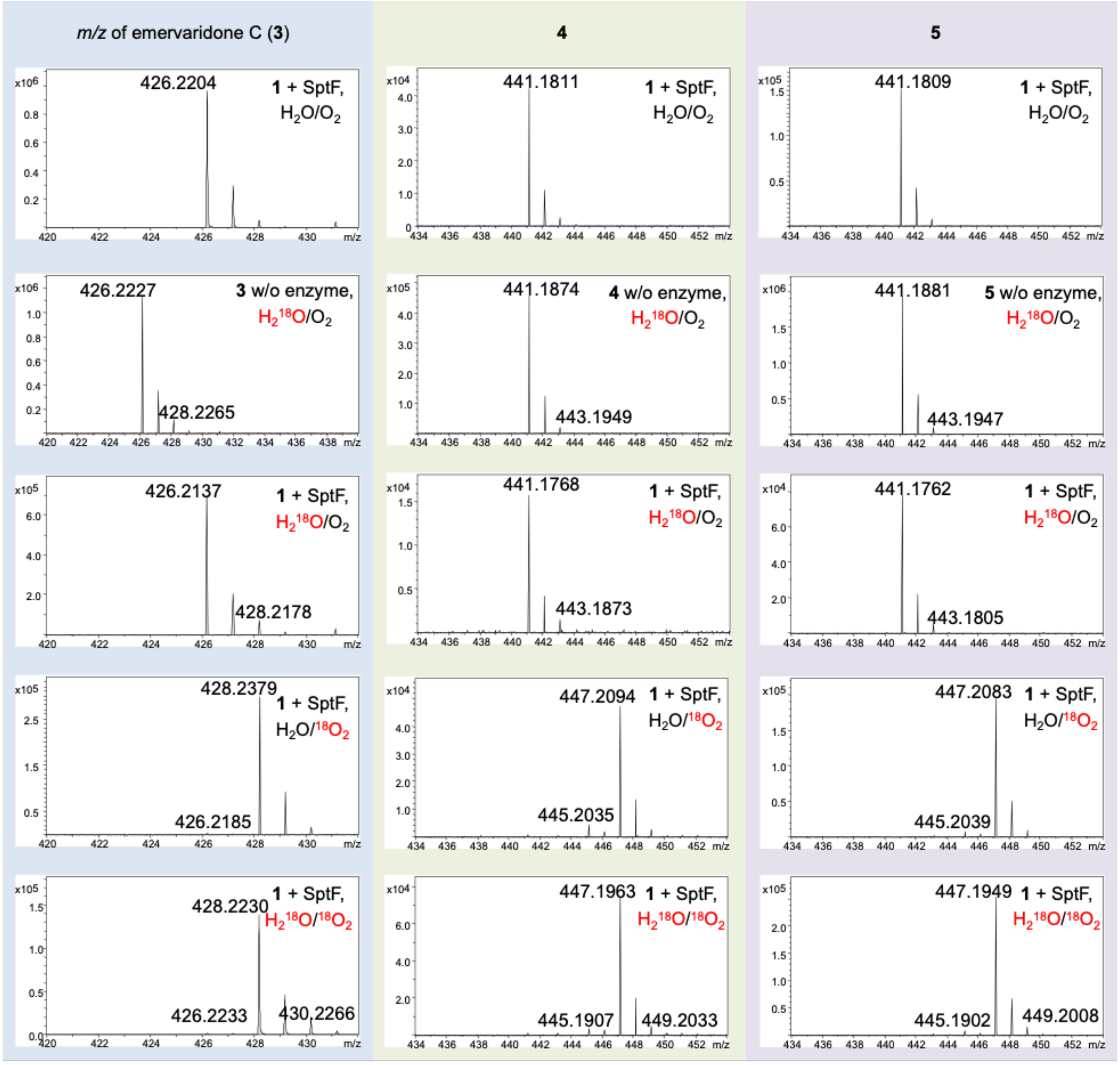

Supplementary Fig. 3 | Results of ^{18}O-labeling experiment.

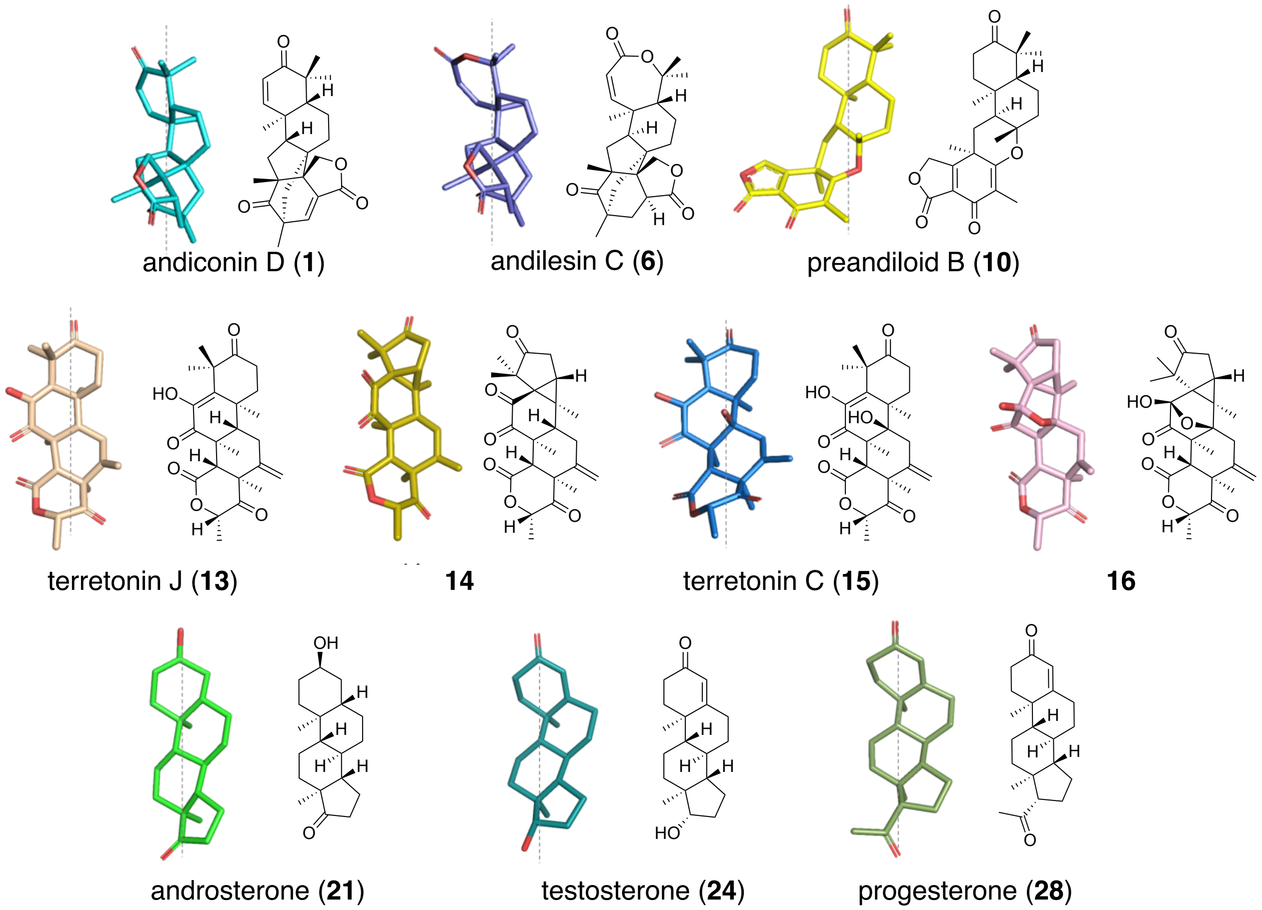

Supplementary Fig. 4 | Model of compounds. Substrates contains hydrophobic and hydrophilic parts, which makes good binding via interactions with the hydrophobic and hydrophilic residues in the active site cavity.

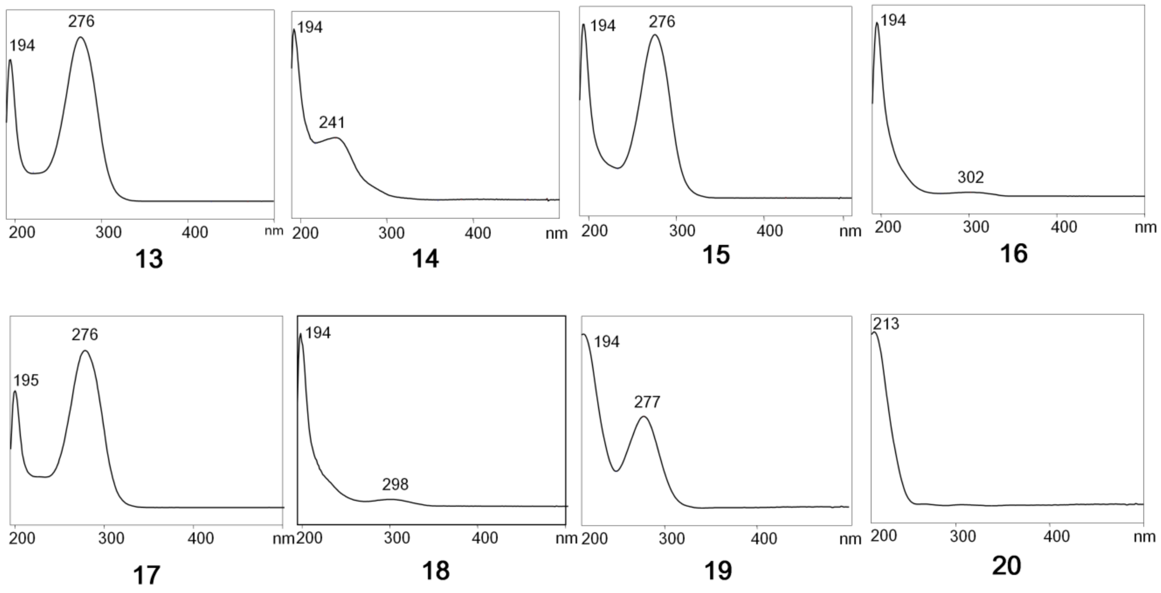

Supplementary Fig. 5 | UV spectra of 13-20.

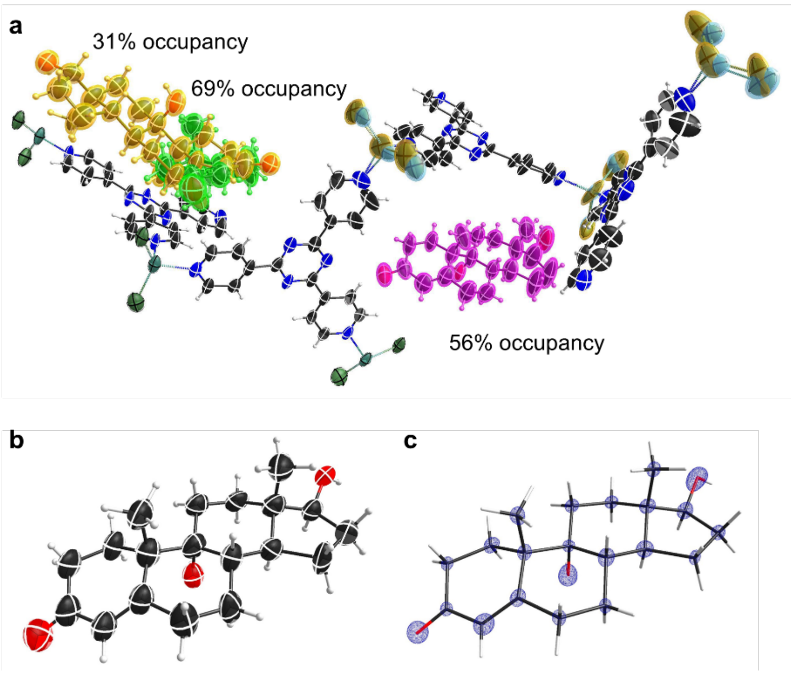

Supplementary Fig. 6 | Crystal structure of the complex of 25 and the crystalline sponge. a, Structure of an asymmetric unit containing three crystallographically independent molecules of **25**. Solvent molecules have been omitted for clarity. **b**, ORTEP drawing with 50 % probability for **25**. **c**, Crystal structure of **25** superimposed with the Fourier electron density map (3.6 eÅ^3 (σ = 1.3)).

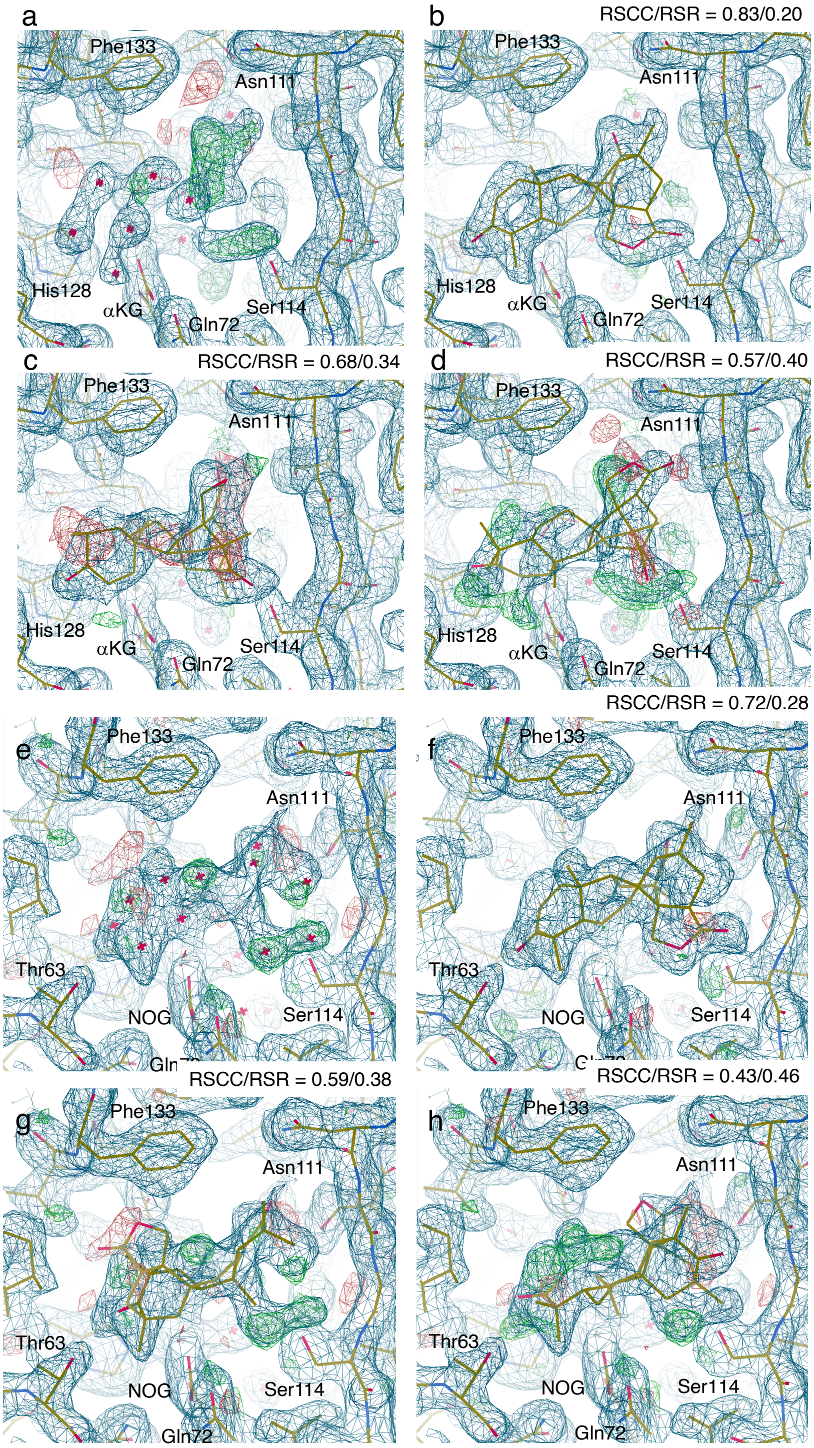
a
Phe133
Asn111
His128
αKG
Gln72
Ser114
b
RSCC/RSR = 0.83/0.20
Phe133
Asn111
His128
αKG
Gln72
Ser114
c
RSCC/RSR = 0.68/0.34
Phe133
Asn111
His128
αKG
Gln72
Ser114
d
RSCC/RSR = 0.57/0.40
Phe133
Asn111
His128
αKG
Gln72
Ser114
RSCC/RSR = 0.72/0.28
e
Phe133
Asn111
Thr63
NOG
Ser114
f
Phe133
Asn111
Thr63
NOG
Ser114
RSCC/RSR = 0.59/0.38
g
Phe133
Asn111
Thr63
NOG
Ser114
Gln72
RSCC/RSR = 0.43/0.46
h
Phe133
Asn111
Thr63
NOG
Ser114
Gln72

(continued)

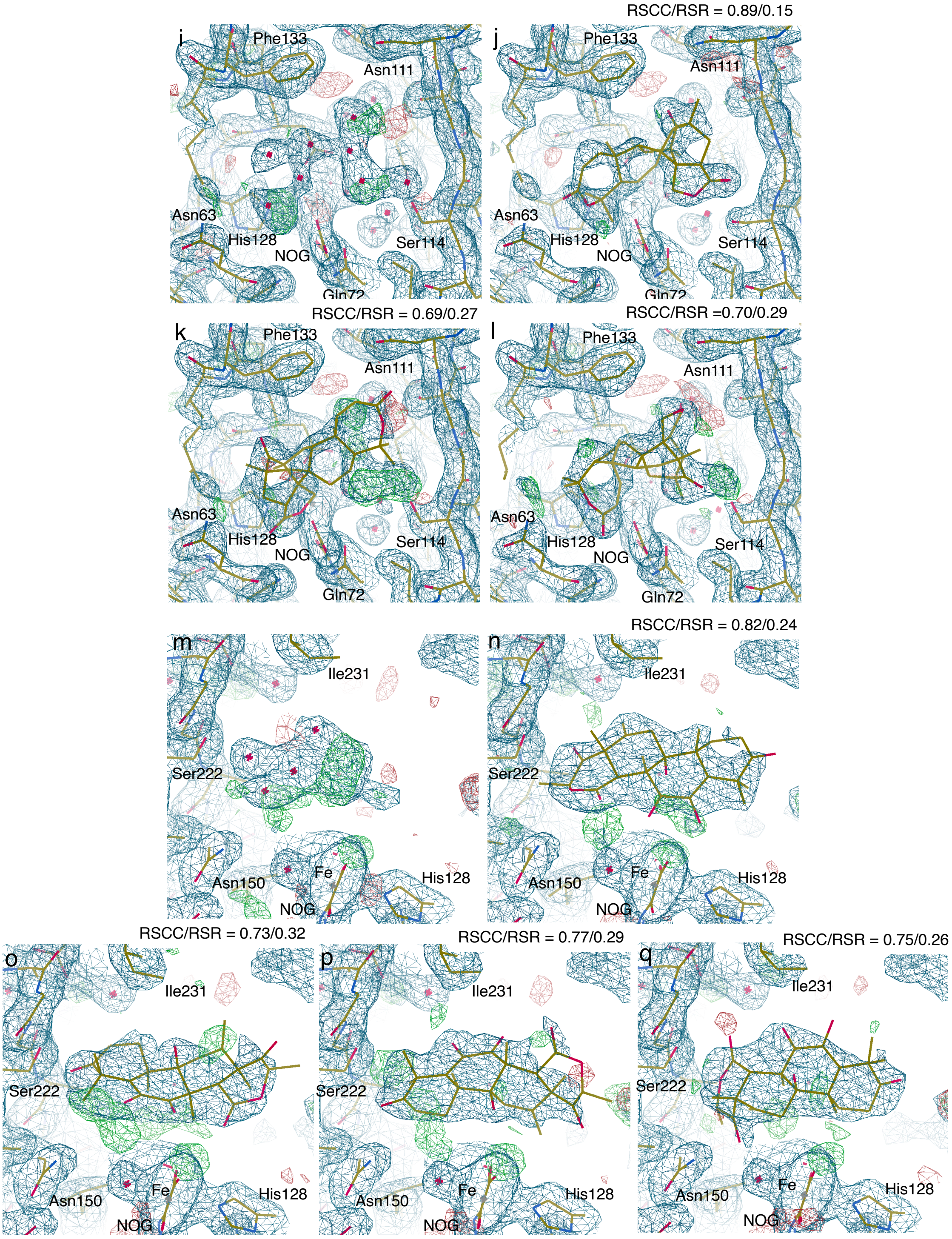

Supplementary Fig. 7 | Fitting of ligands in unassigned density. Comparison of the models fitted in the unassigned electron density map with different conformations. 2*m*Fo-*D*Fc (blue mesh), *m*Fo-*D*Fc maps (positive: green mesh, negative: red mesh) are shown for (**a-d**) SptF-Fe/αKG/**1** (**e-h**) SptF-Fe/NOG/**6**, **(i-l)** SptF N65T-Fe/NOG/**1**, (**m-q**) SptF S114A-Fe/NOG/**15,** respectively. The 2*m*Fo-*D*Fc and *m*Fo-*D*Fc maps and are represented contoured at 0.9 and 3.0 σ, respectively. RSCC and RSR values were calculated by PDB validation server. According to these analyses, we employed models of **b**, **f**, **j**, **n** for further analysis of the binding modes of substrates.

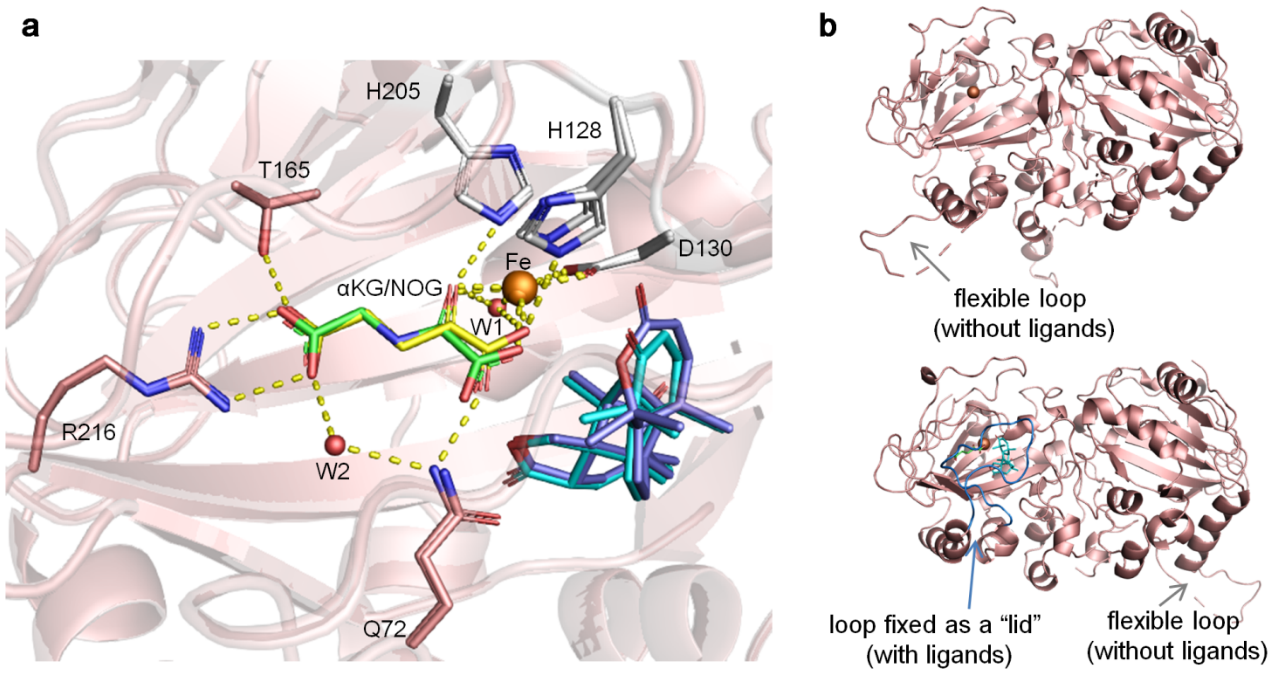

Supplementary Fig. 8 | The active site of SptF. **a**. Conserved 2-His-1-Asp facial triad for metal binding (H205, H128, and D130) is shown as grey sticks. And the binding mode of αKG (in yellow) and NOG (in green) in SptF-Fe/αKG/**1** and SptF-Fe/NOG/**6** are almost identical, and these ligands interact with the facial triad, Gln72, Thr165, and Arg216 via hydrogen bonding networks. **b**, the density of the loop between Trp53 and Asn75 of SptF (shown in blue) was observed only upon substrate binding, and it serves as an lid to encapsulate the substrate in the active site of SptF. Note that water molecules are shown as red spheres, and Fe is shown as orange spheres. **1** and **6** are shown as cyan and purple sticks, respectively. Hydrogen networks are shown as yellow dash lines.

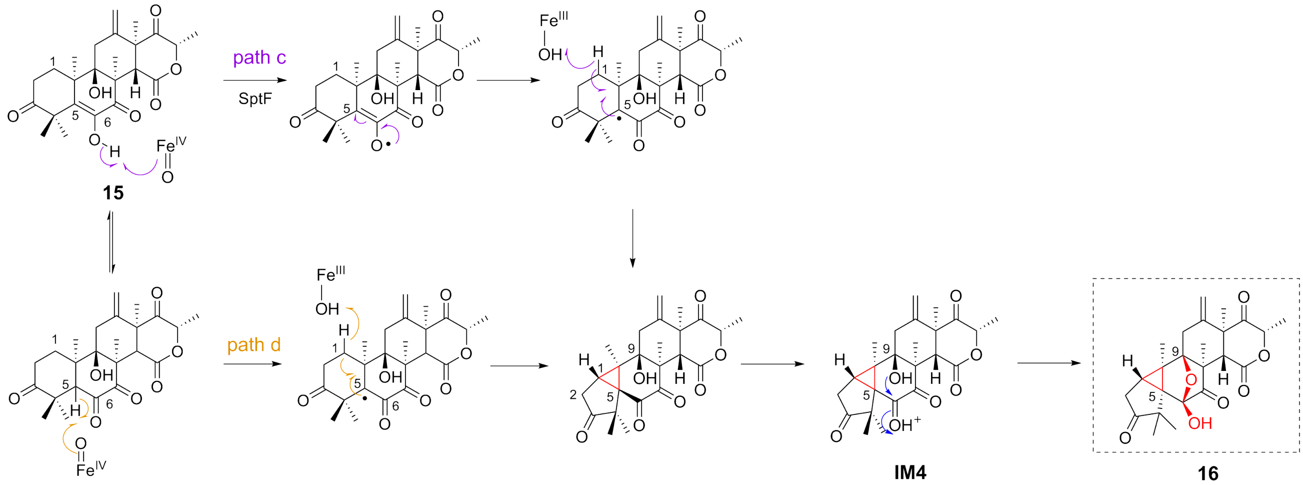

Supplementary Fig. 9 | Proposed mechanism for generation of 16 from terretonin C (15). For path c (shown in purple)**,** the enolic hydrogen is abstracted to form a radical, which leads to the formation of a highly congested cyclopropane ring in IM4 via radical recombination. For path d (shown in orange), the reaction starts with abstraction of hydrogen atom at C5 on the tautomer of **15**, and subsequently radical undergoes recombination to form the cyclopropane ring in **IM4**, which is converted to **16** spontaneously via hemiacetal formation reaction.

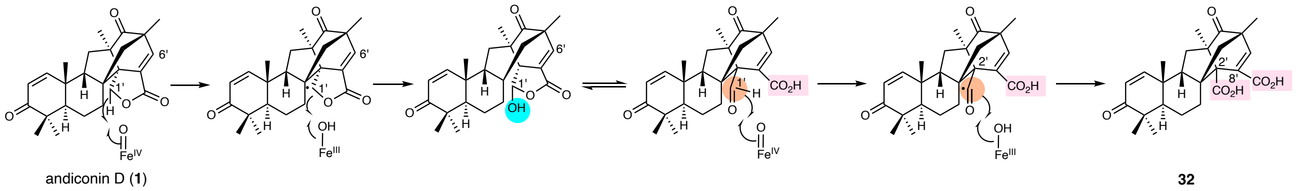

Supplementary Fig. 10 | Proposed mechanism for generation of 32 from 1 by mutants. 32 was proposed to be generated via two rounds of hydroxylations at C1' position of **1** by SptF mutants.

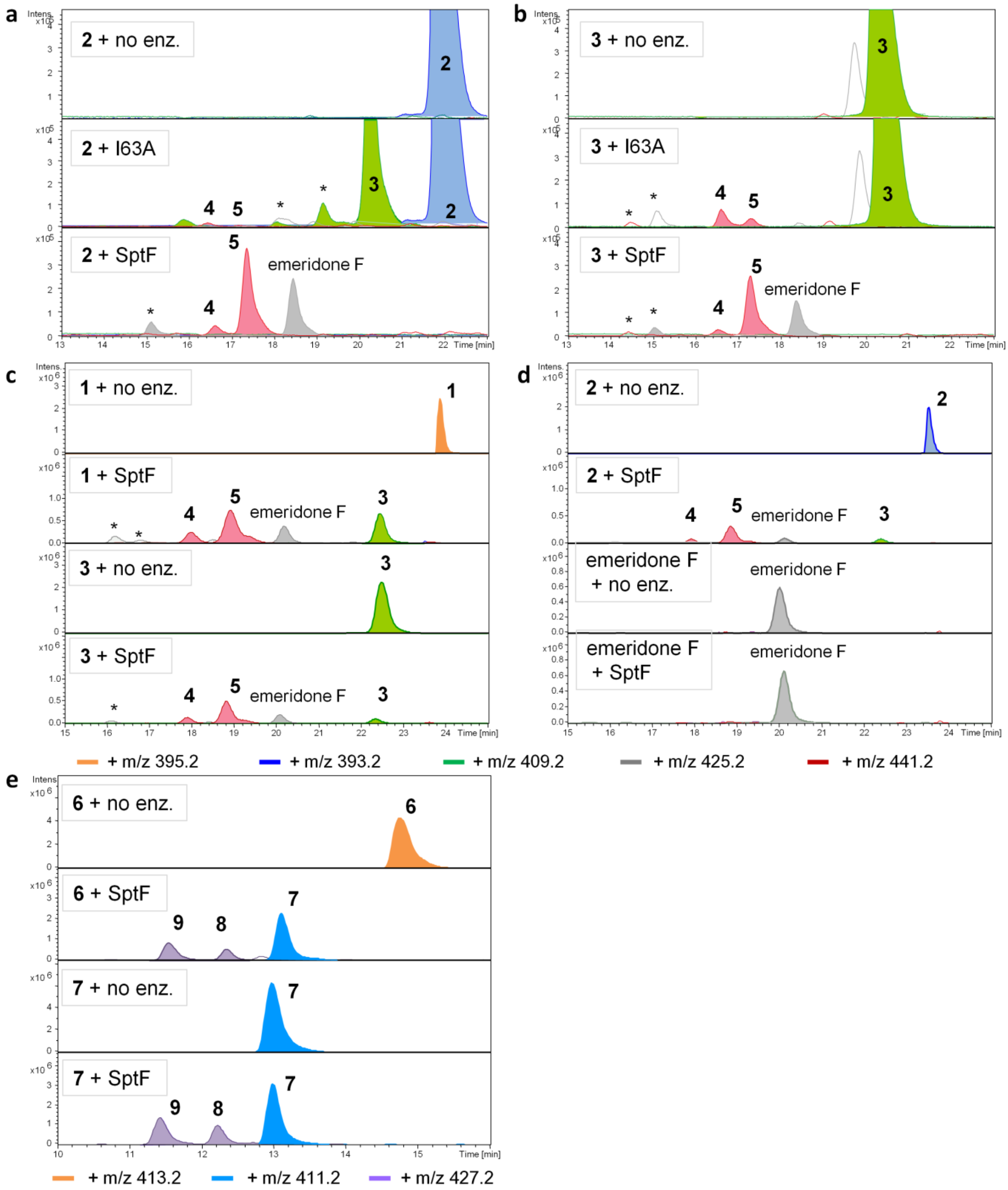

Supplementary Fig. 11 | In vitro assays with mutants or intermediates. a, **b**, using **2** or **3** as substrate with I163A mutant or wild-type SptF. **c**-e, using **1**-**3**, emeridone F, **6** or **7** as substrate with wild-type SptF to determine the reaction order from **1** or **6**. Peaks marked with stars are structure undetermined enzymatic products due to unstability or low yields. For I63A mutant, 40 μM enzyme was incubated with 100 μM substrate at 30 ºC for 24 hrs. For reactions with possible intermediate**s**, 15 μM SptF was used to incubate with 100 μM substrate at 30 ºC for 1 hr. m/z for EIC of each compounds were shown.

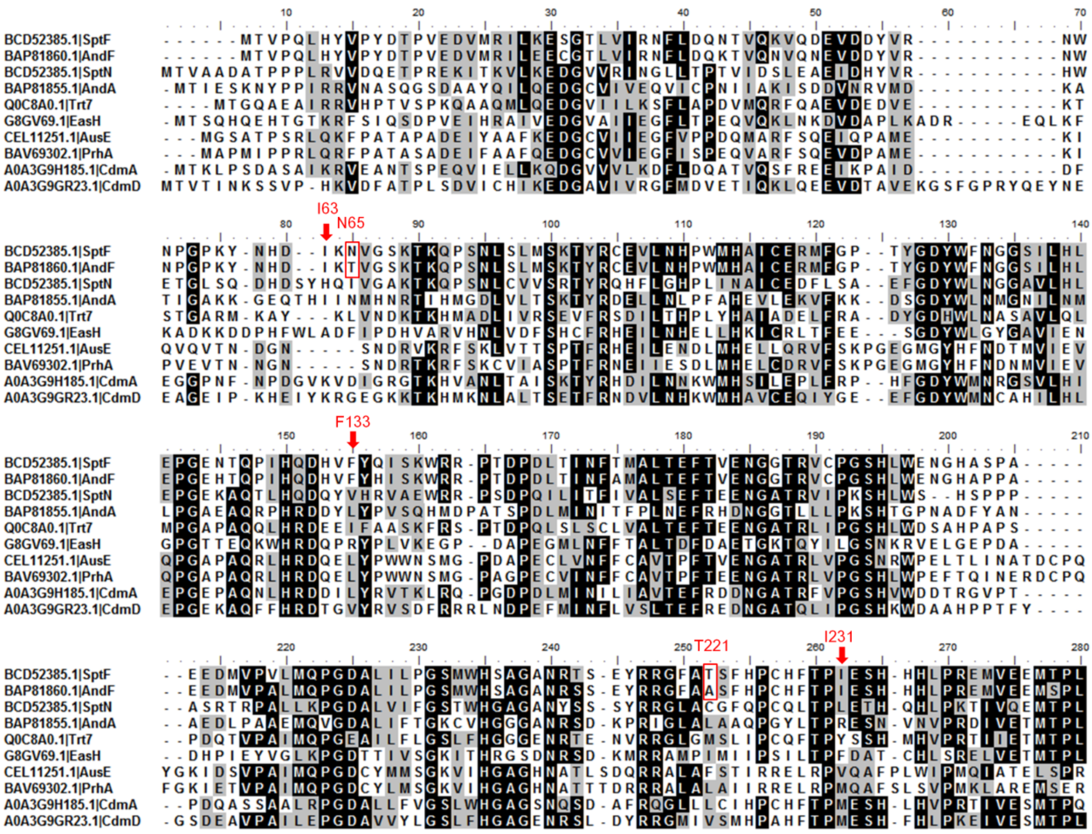

Supplementary Fig. 12 | The sequence alignment of SptF with other Fe(II)/αKG-dependent oxygenases. SptF and AndF share 94.4% amino acid sequence identity. Asn65 and Thr221 in SptF is substituted with threonine and alaline in AndF, respectively, as shown in the red boxes. Three hydrophobic residues (I63, F133, and I231) involved in substrate recognition are marked with red arrows. Shading threshold for identity (black) and similarity (gray) is 60% and 30%, respectively.

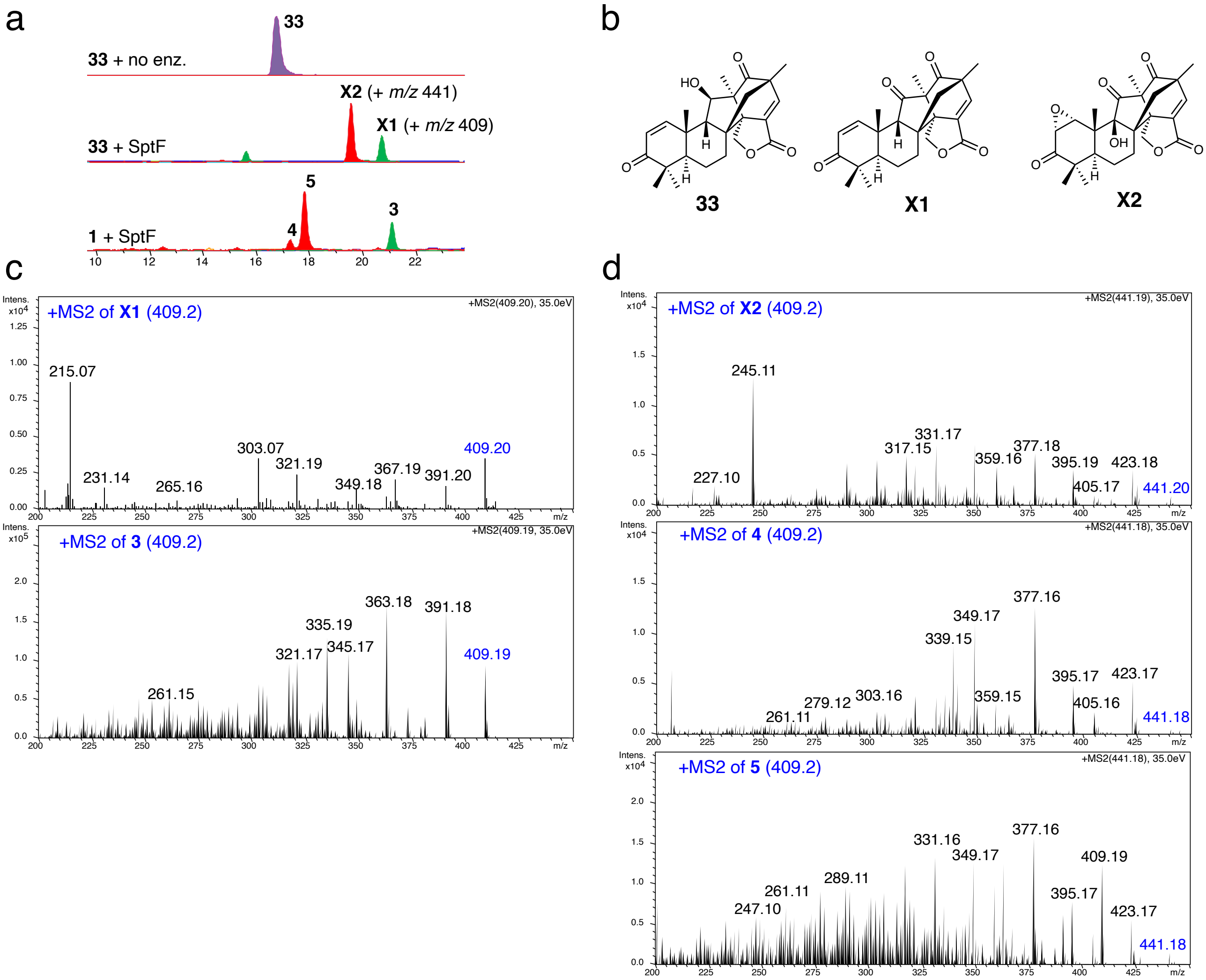

Supplementary Fig. 13 | *In vitro* assay using SptF and 33. a. LC-MS analysis of enzyme reactions of SptF with **1** and **33**. Structures of **X1** and **X2** could not be determined because of instability and low yield. **b**. Structure of **33**, and postulated structures of **X1** and **X2** based on MS analyses. **c**. Comparison of the MS/MS fragments of **3** and **X1**. **d**. Comparison of the MS/MS fragments of **4**, **5**, and **X1**. Here 15 µM SptF was incubated with 100 µM **33** at 30 ºC for 1 hr.

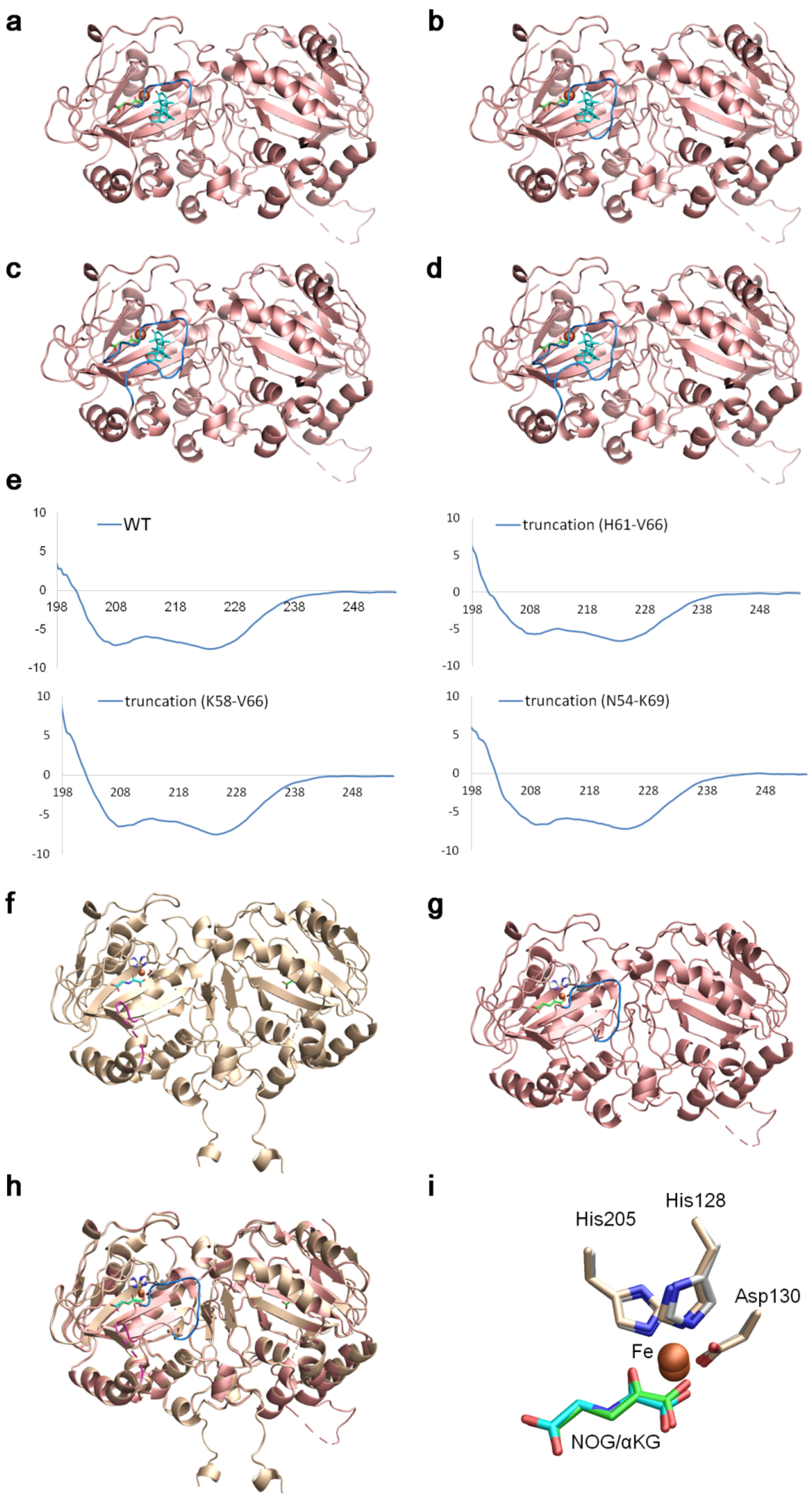

Supplementary Fig. 14 | Loop truncation. a, 6 residues (His61-Val66); **b**, 9 residues (Lys58-Val66); **c**, 16

residues (Asn54-Lys69); **d**, 19 residues (Trp53-Lys71) which cover the active site, were truncated from SptF. Truncated regions were shown as blue cartoon. **e**, CD spectra of SptF wild-type and loop truncation variants. **f**, **g**, crytal structures of 9-residue (Lys58-Val66) truancated mutant and wild-type SptF. **h**, **i**, alignment of overall structures and a close view of active sites in 9-residue truncated mutant (wheat) and wild-type SptF (salmon), respectively. NOG and αKG were shown as cyan and green sticks, respectively.

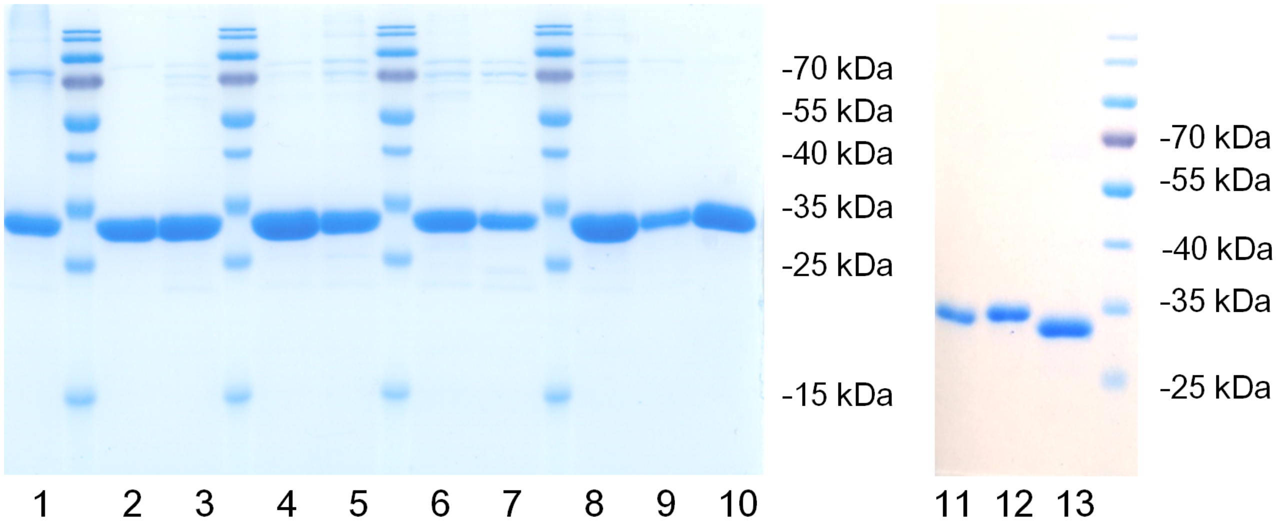

Supplementary Fig. 15 | SDS-PAGE analysis of His-tagged proteins. Lane 1-13: 63A, N65A, N65T, S114A, F133A, F133Y, T148S, N150A, I231A, SptF, loop truncation mutant Δ6 (Lys58-Val66), Δ9 (H61- Val66), Δ16 (Asn54-Lys69). All experiments were repeated independently more than three times with similar results.

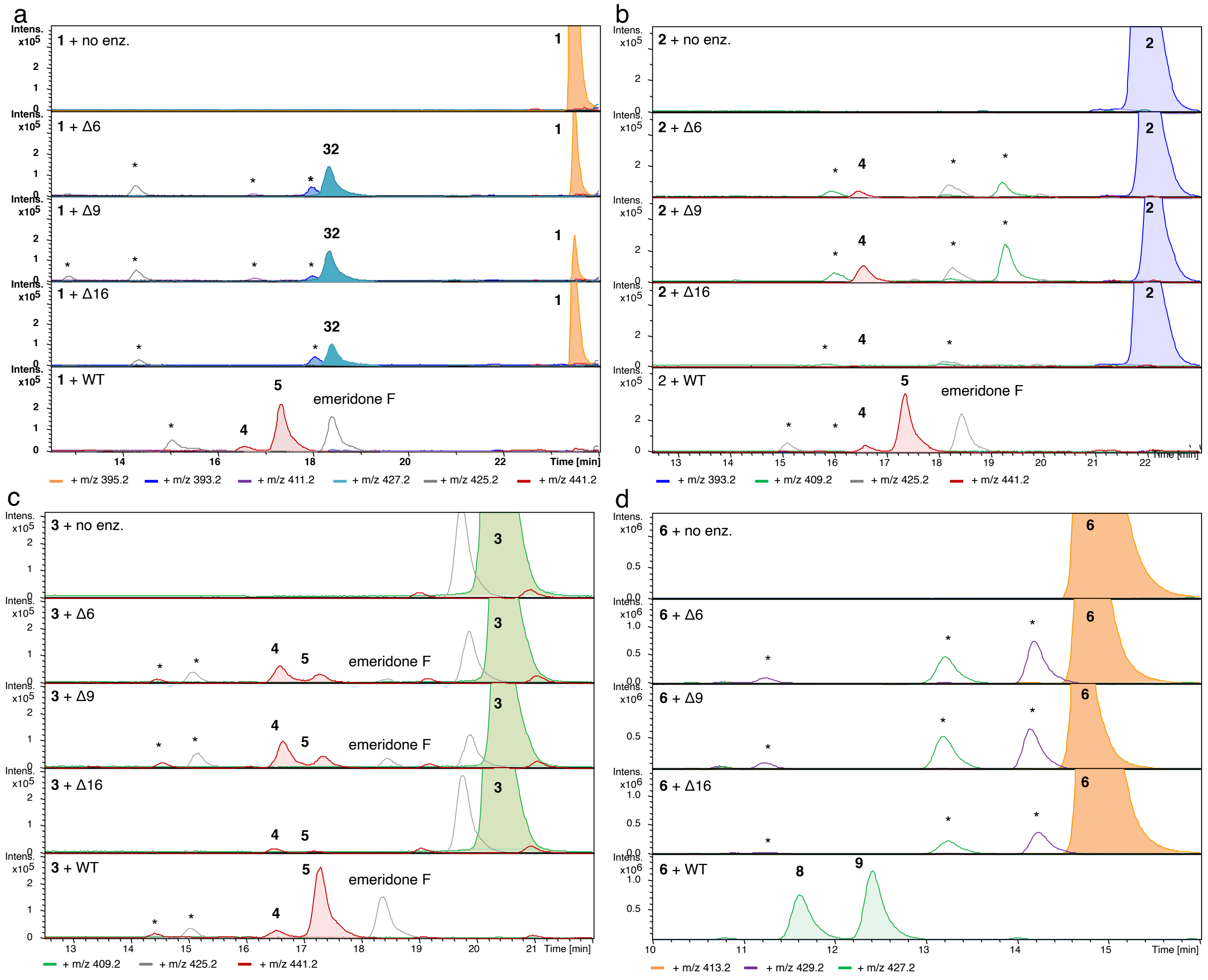

Supplementary Fig. 16 | In vitro assay using SptF loop truncation mutants with a-d, 1-3, or 6 as substrate, respectively. Peaks marked with stars are structure undetermined enzymatic products due to low yields. 40 μM loop truncation mutant was incubated with 100 μM substrate at 30 ºC for 24 hrs.

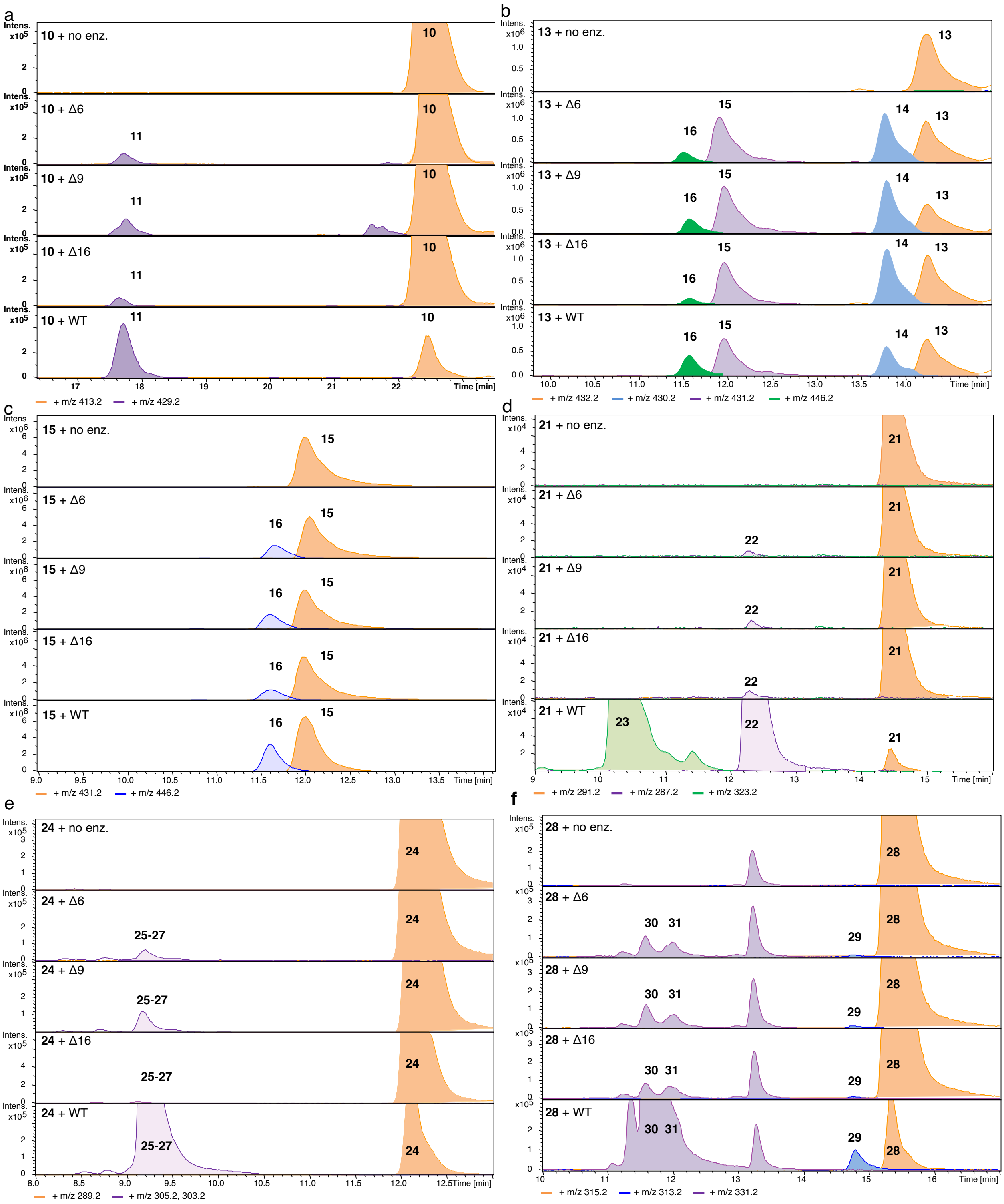

Supplementary Fig. 17 | In vitro assay using loop truncation mutants with unnatural substrates a-f, 10, 13, 15, 21, 24, 28, respectively. 40 μM SptF truncation mutants were incubated with 100 μM substrate at 30 ºC for 24 hrs, and conversion rates were shown in Supplementary Table 5.

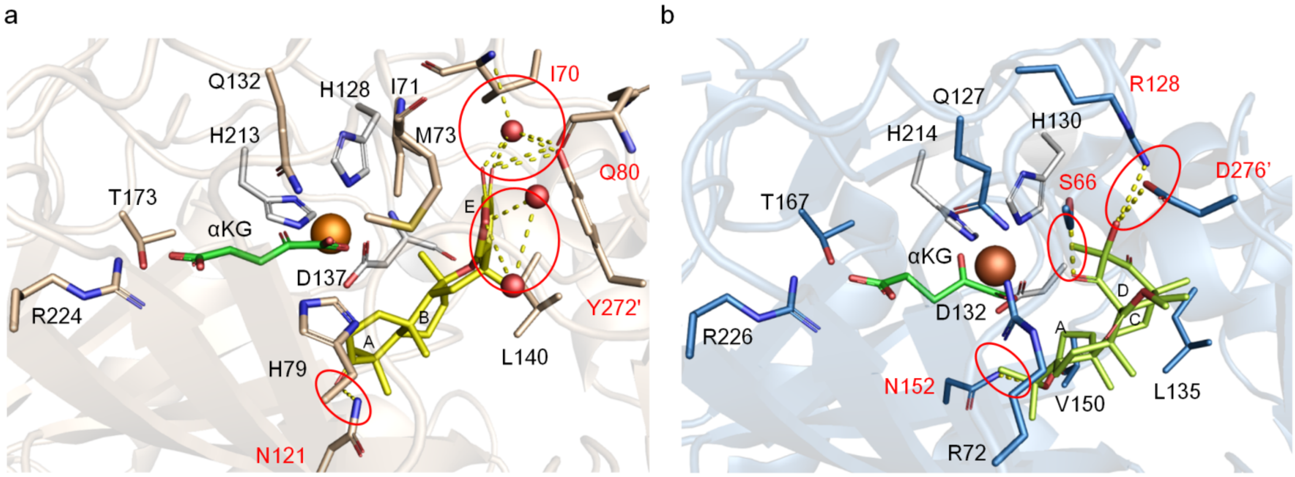

Supplementary Fig. 18 | The substrate binding mode in AndA and PrhA. a, In AndA (PDB:5ZM3, shown in wheat color) case, the D/E rings of preandiloid B (**10,** yellow sticks**)** was firmly fixed by residues (I70 and Q80) located on the lid-like loop and Y272' from ajacent monomer via hydrogen bond network, and A-ring of **10** was fixed by only one hydrogen bond via N121 in deeper cavity. **b**, Similarly, in PrhA case (PDB: 5YBO, shown in blue color), the A-ring of preausinoid A1 (shown as limone sticks) was firmly fixed with hydrogen bond networks via S66, R128, and D276', whereas the D-ring of preausinoid A1 was fixed by only one hydrogen bond via N152 in the deeper cavity. Note that water molecules are shown as red spheres, and Fe is shown as orange spheres. αKG was shown as green sticks. Hydrogen networks are shown as yellow dash lines in the red boxes.

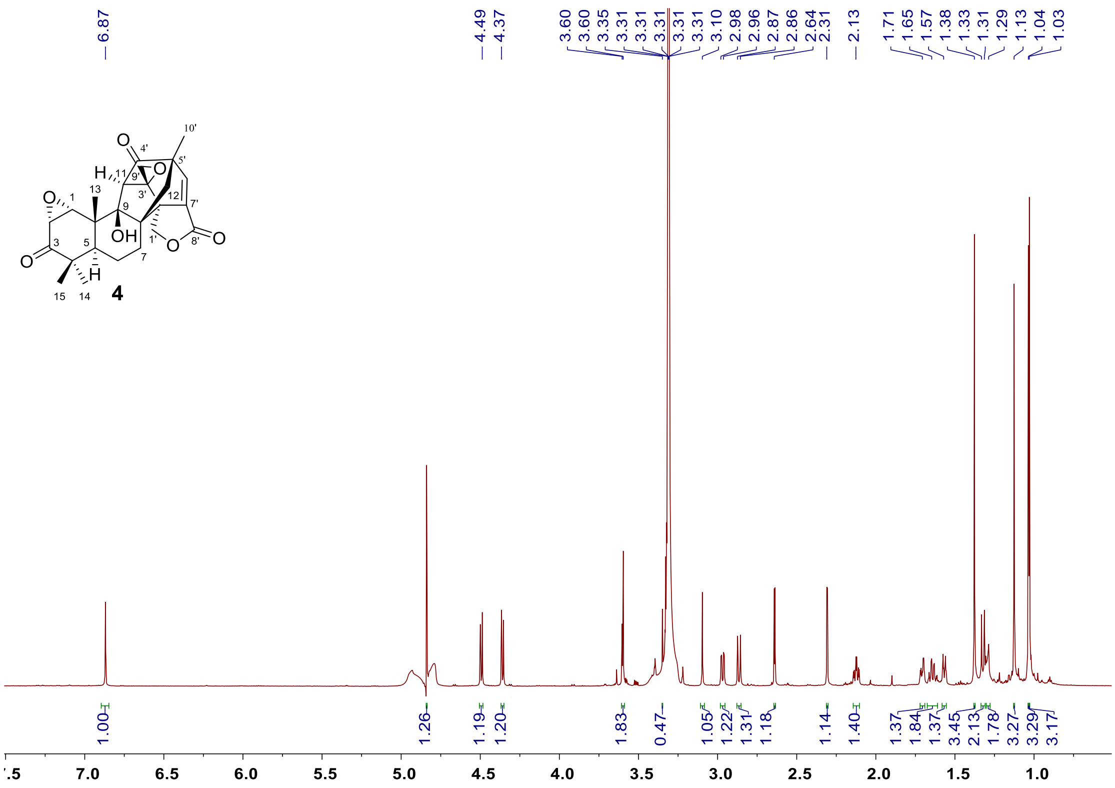

Supplementary Fig.19 | ^{1}H NMR spectrum of 4 in CD3OD.

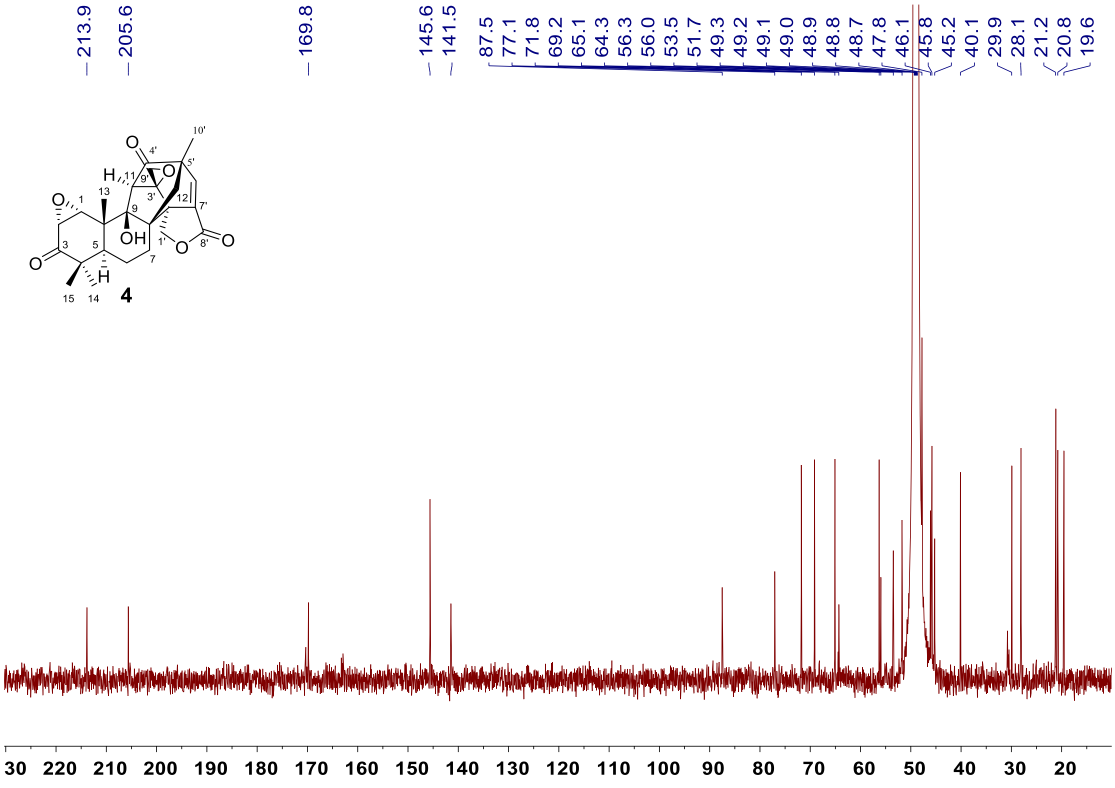

Supplementary Fig. 20 | ^{13}C NMR spectrum of 4 in CD3OD.

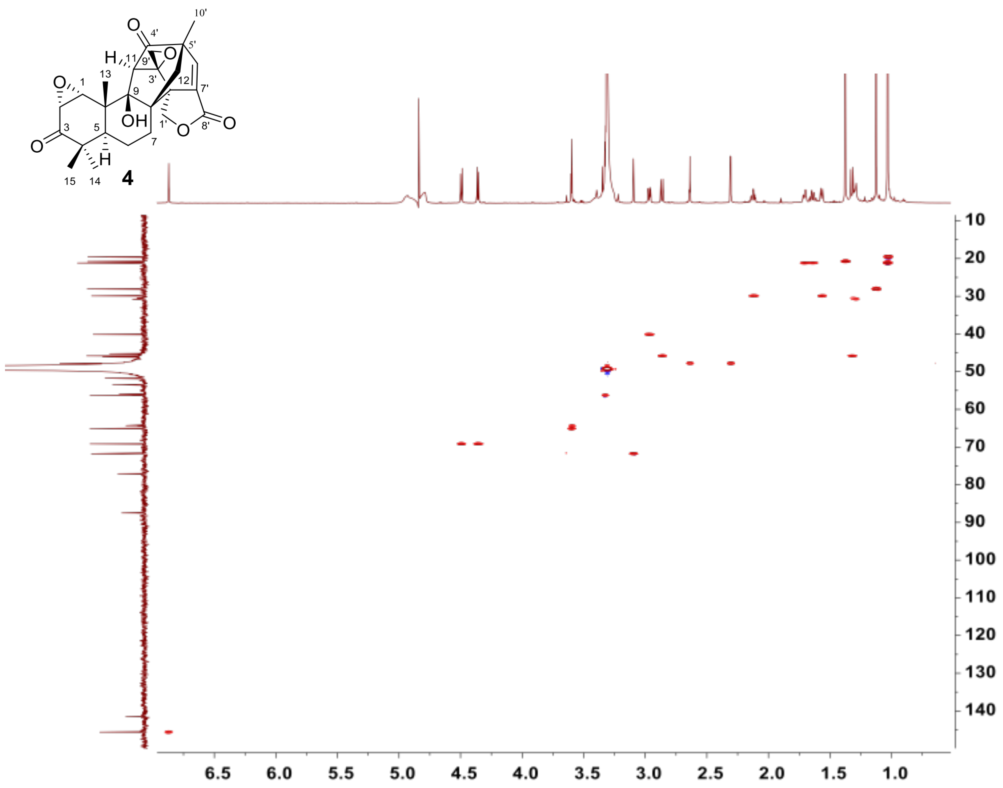

Supplementary Fig. 21 | HSQC NMR spectrum of **4** in CD_3OD.

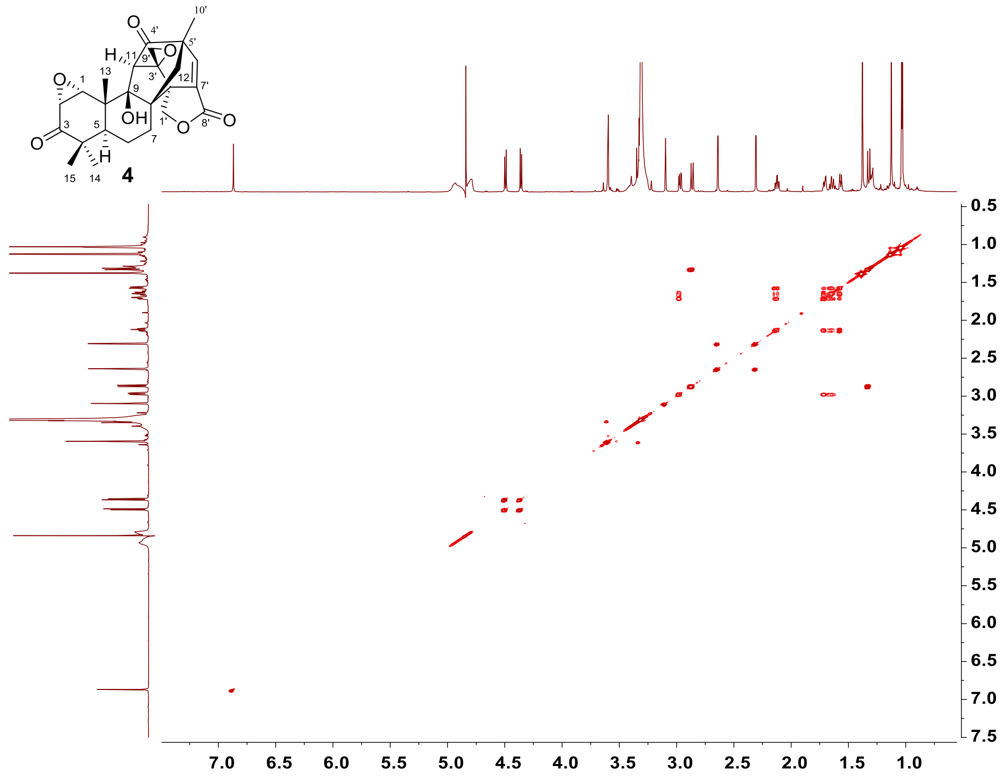

Supplementary Fig. 22 | COSY NMR spectrum of **4** in CD_3OD.

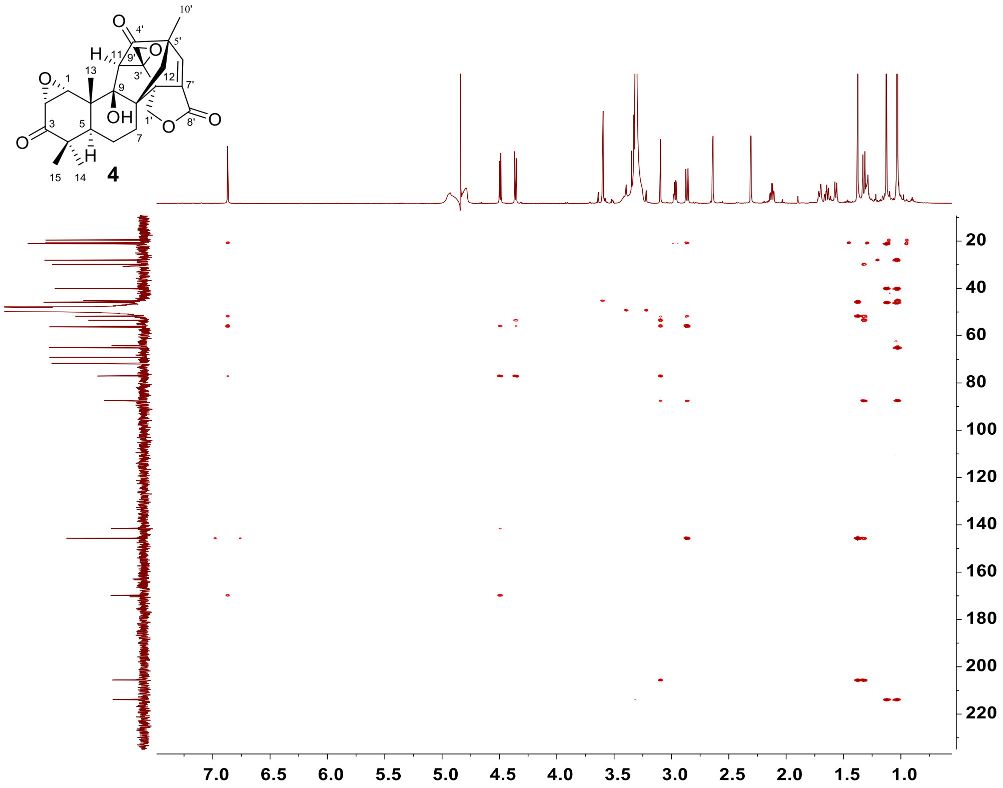

Supplementary Fig. 23 | HMBC NMR spectrum of 4 in CD3OD.

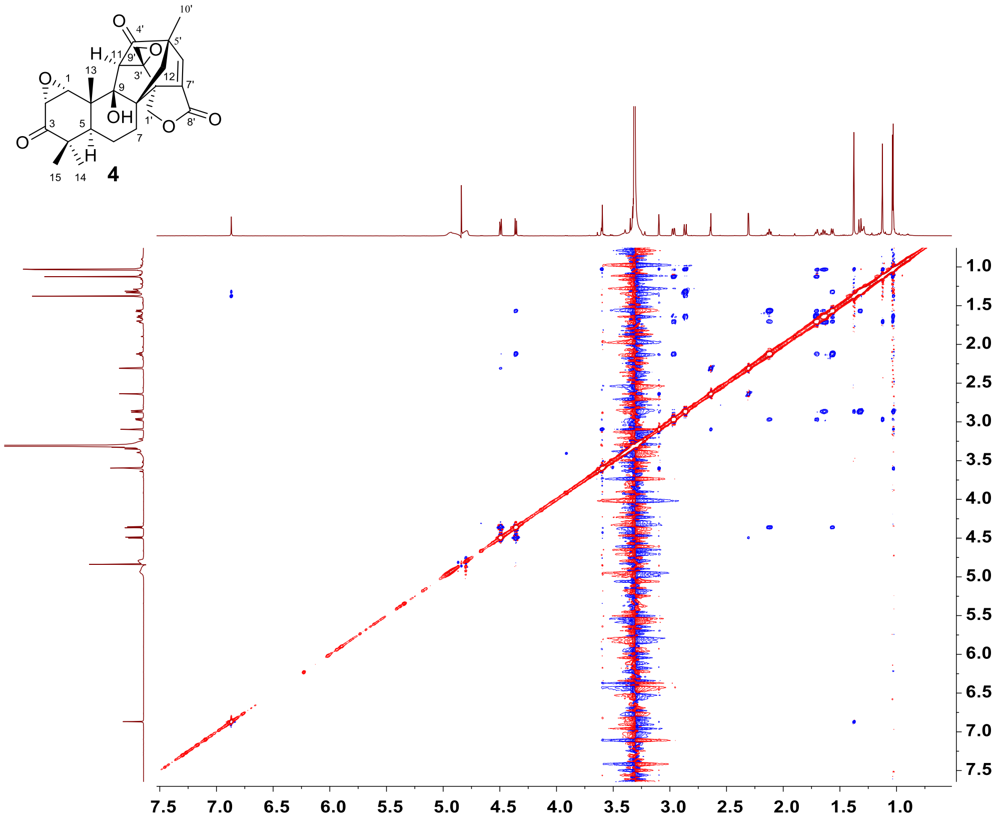

Supplementary Fig. 24 | NOESY NMR spectrum of 4 in CD3OD.

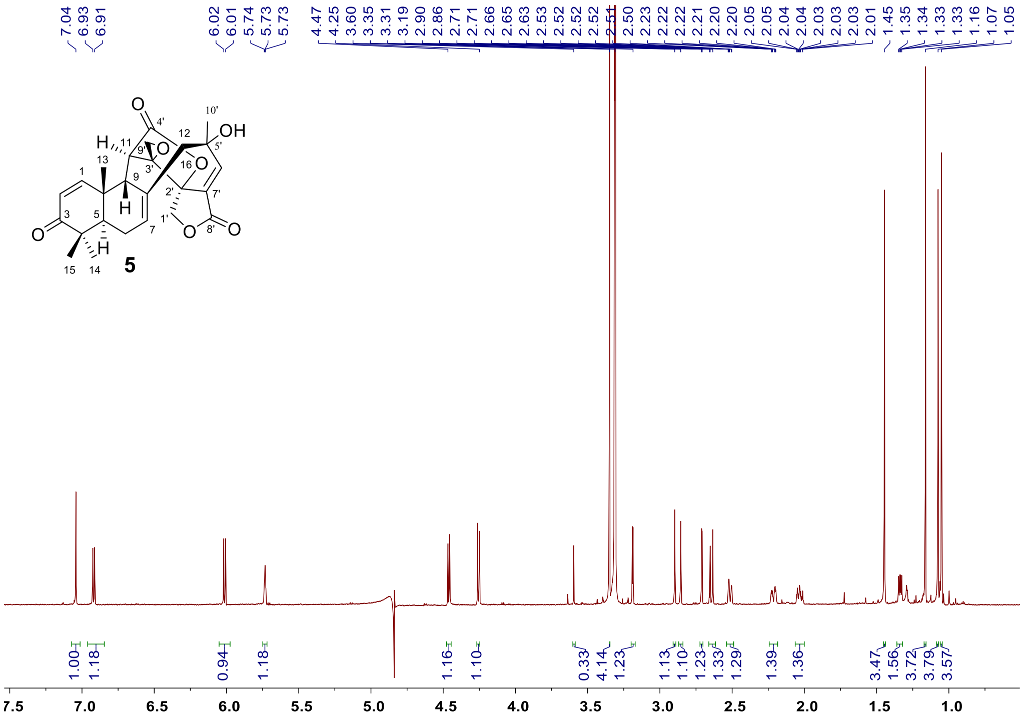

Supplementary Fig. 25 | ** ^{1}H NMR spectrum of **5 in CD3OD.

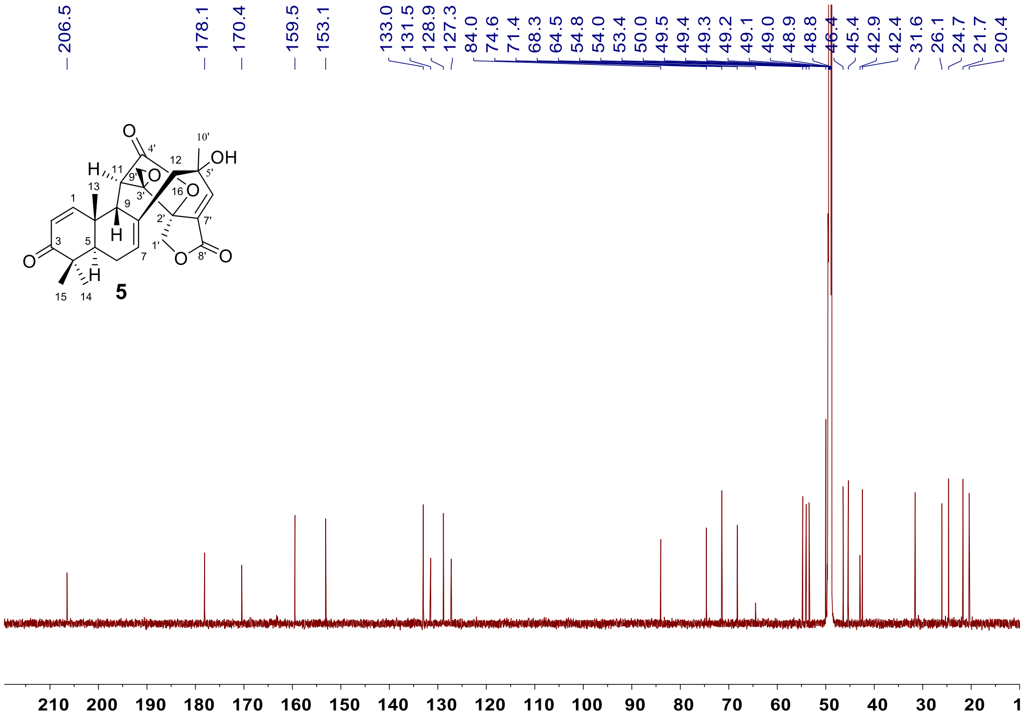

Supplementary Fig. 26 | ** ^{13}C NMR spectrum of **5 in CD3OD.

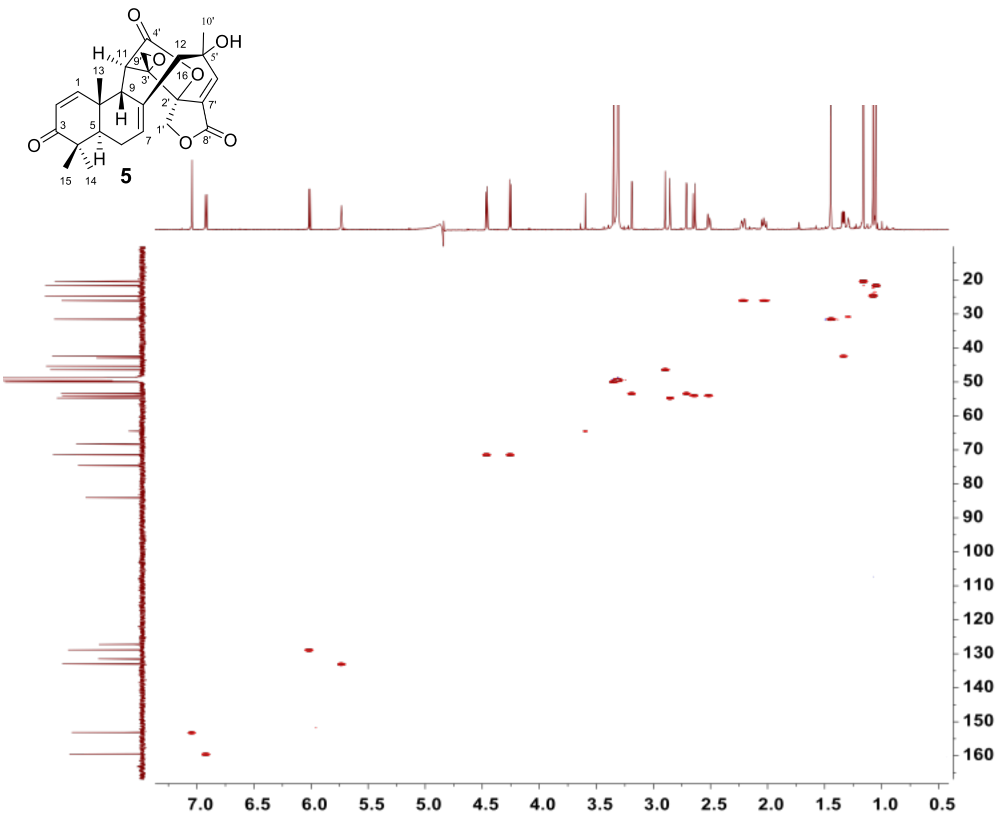

Supplementary Fig. 27 | HSQC NMR spectrum of **5** in CD3OD.

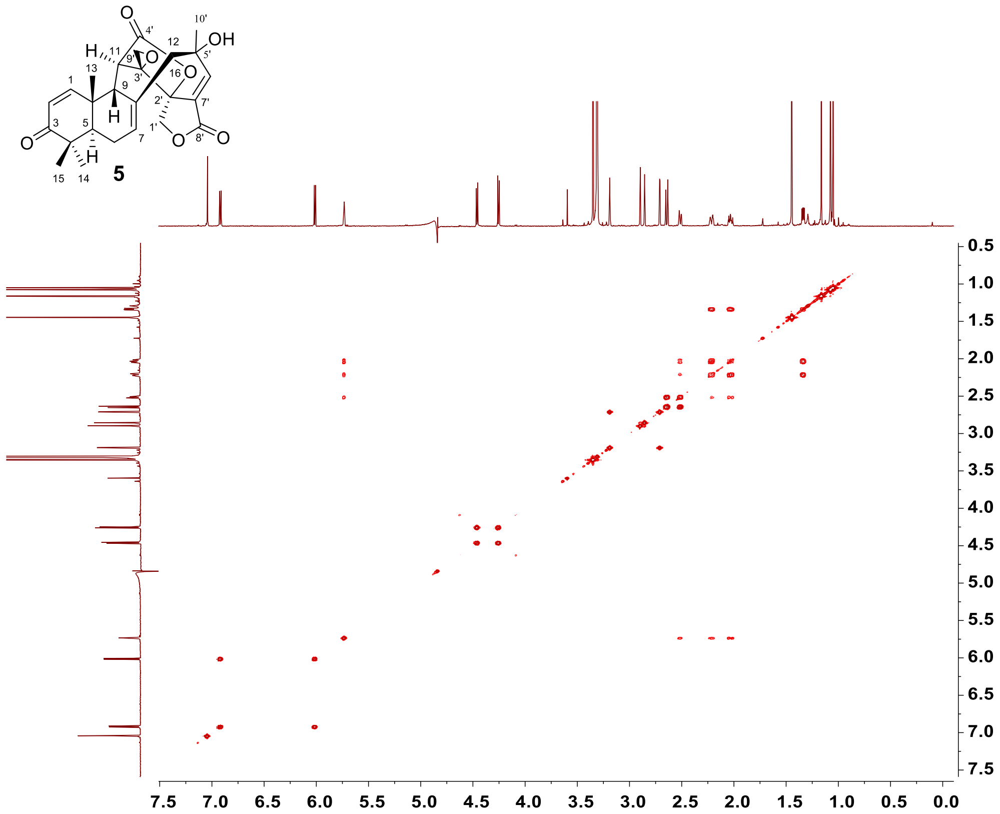

Supplementary Fig. 28 | COSY NMR spectrum of **5** in CD3OD.

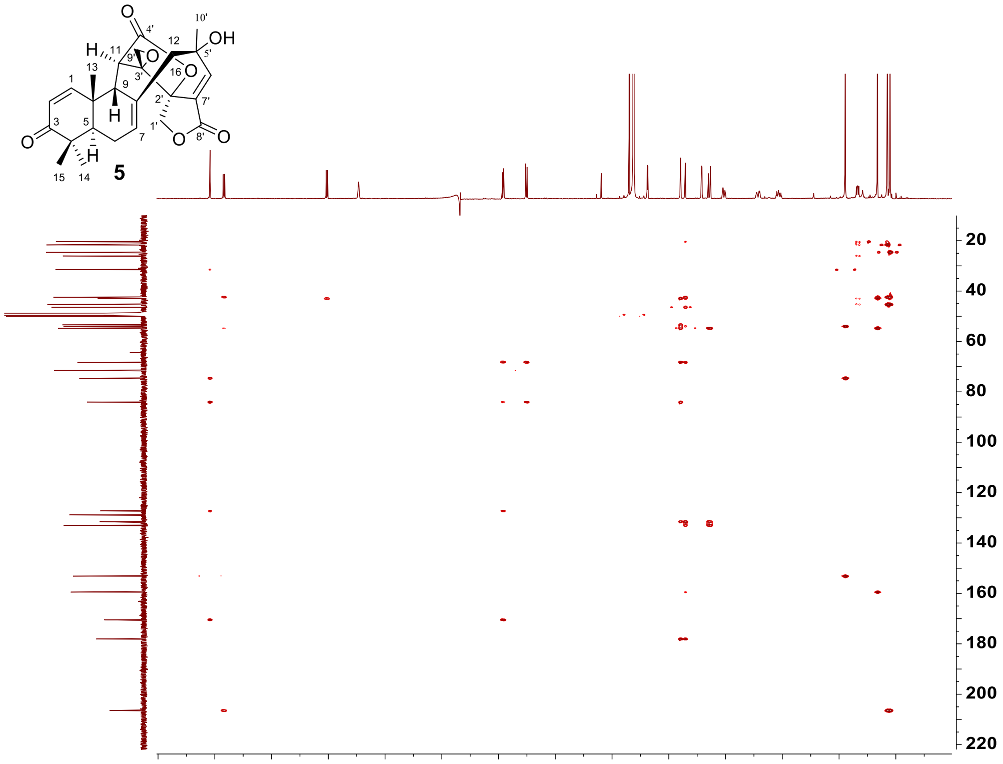

Supplementary Fig. 29 | ** HMBC NMR spectrum of **5 in CD_3OD.

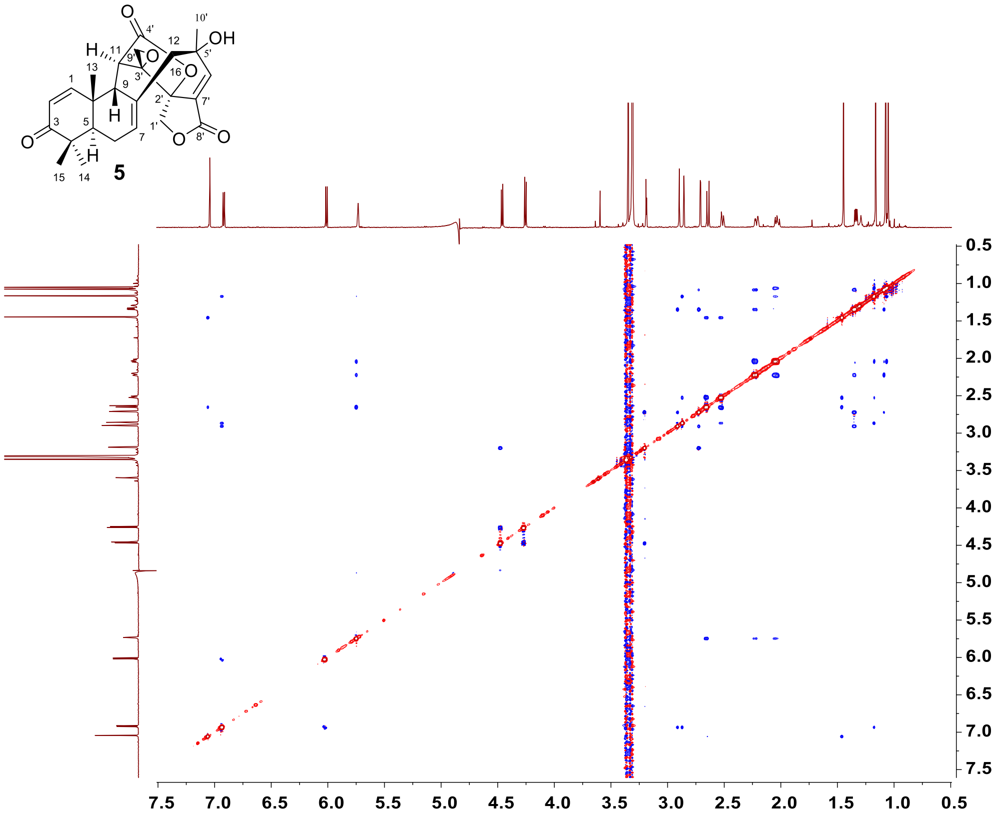

Supplementary Fig. 30 | ** NOESY NMR spectrum of **5 in CD_3OD.

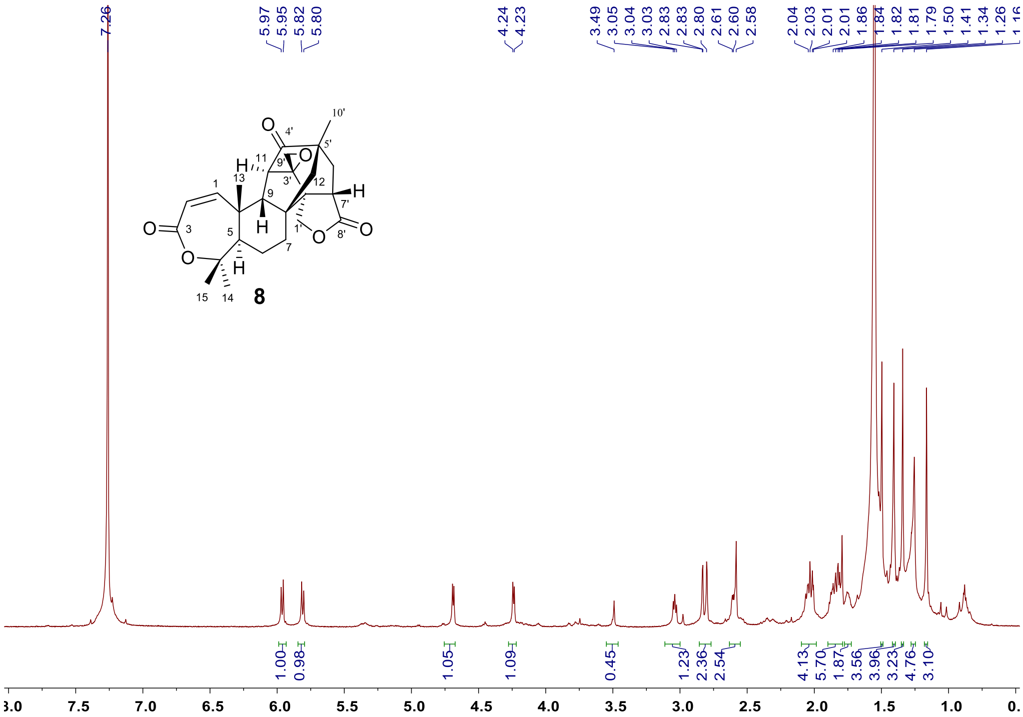

Supplementary Fig. 31 | ^{1}H NMR spectrum of **8** in $CDCl_3$.

1

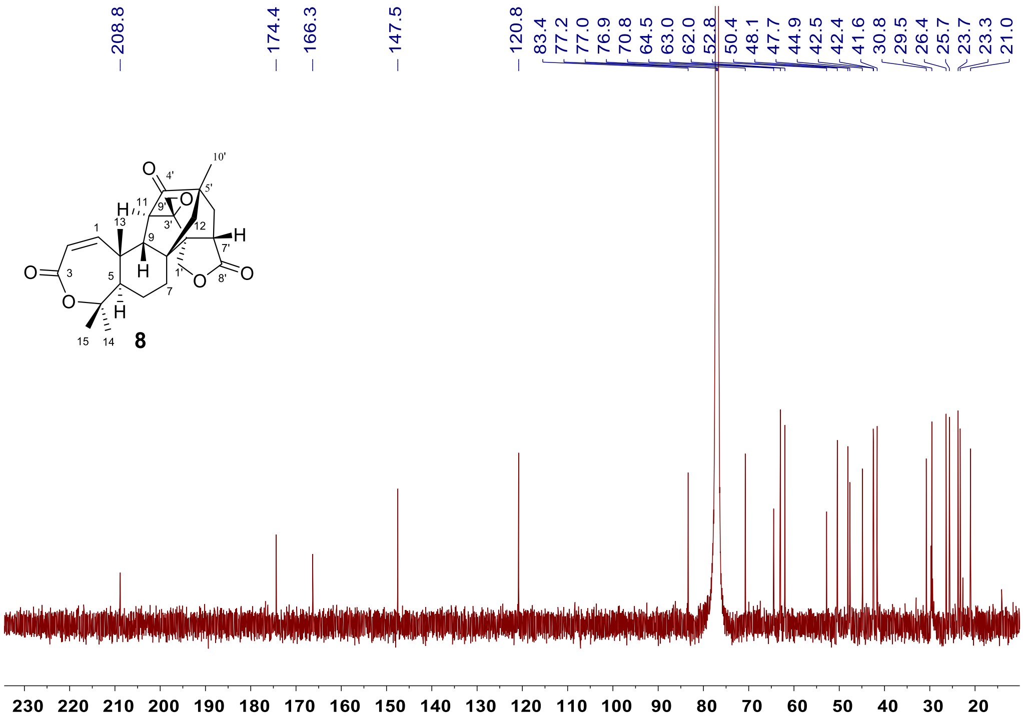

Supplementary Fig. 32 | ^{13}C NMR spectrum of **8** in $CDCl_3$.

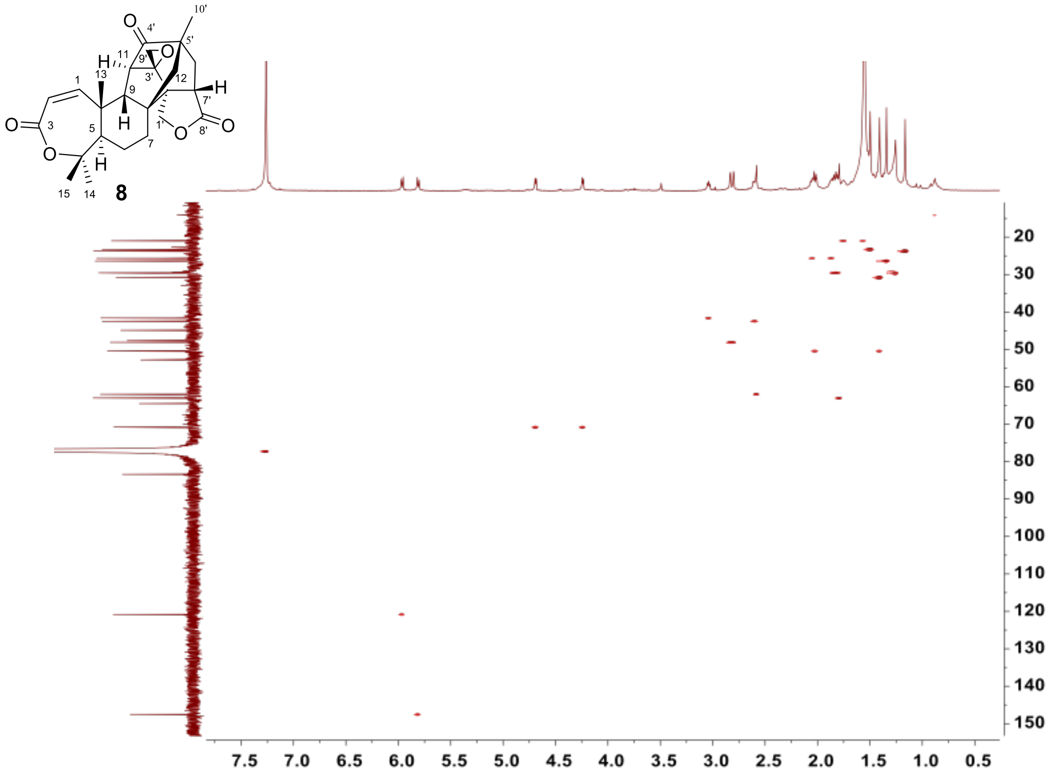

Supplementary Fig. 33 | HSQC NMR spectrum of 8 in $CDCl_3$.

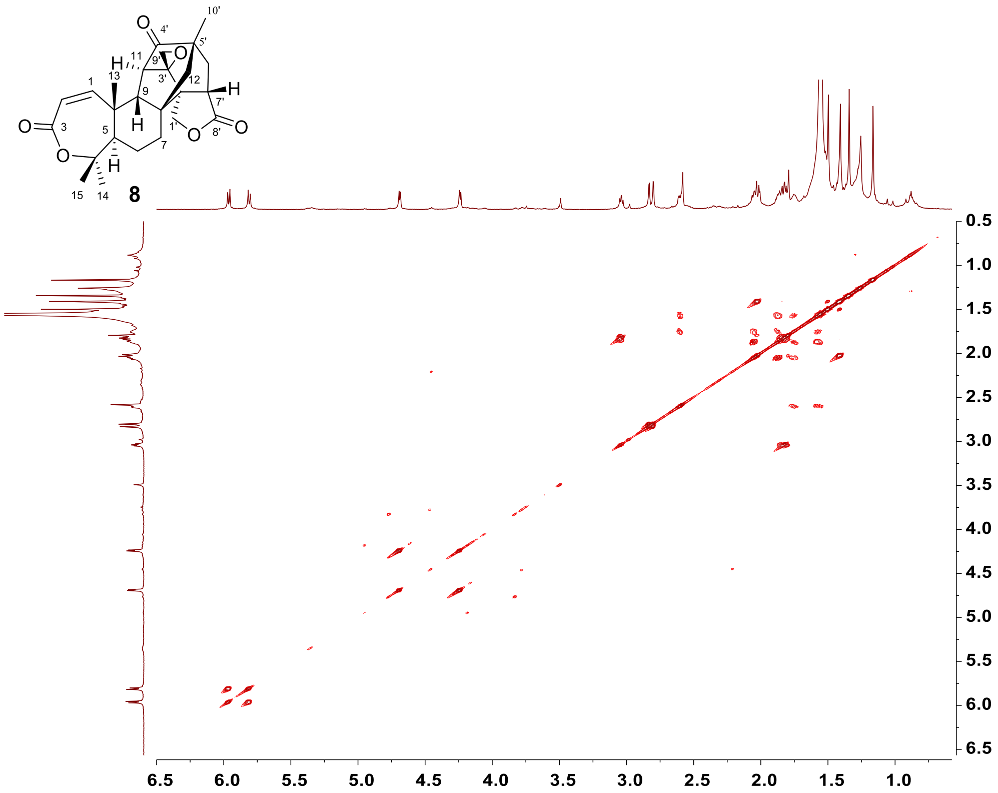

Supplementary Fig. 34 | COSY NMR spectrum of 8 in $CDCl_3$.

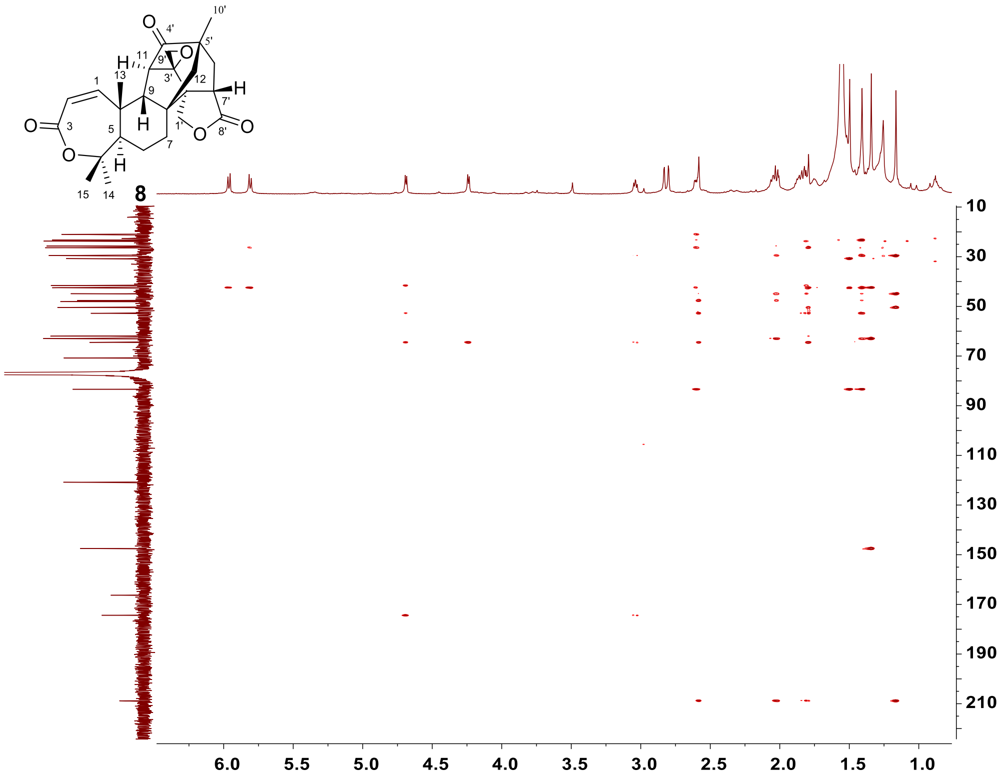

Supplementary Fig. 35 | HMBC NMR spectrum of 8 in CDCl3.

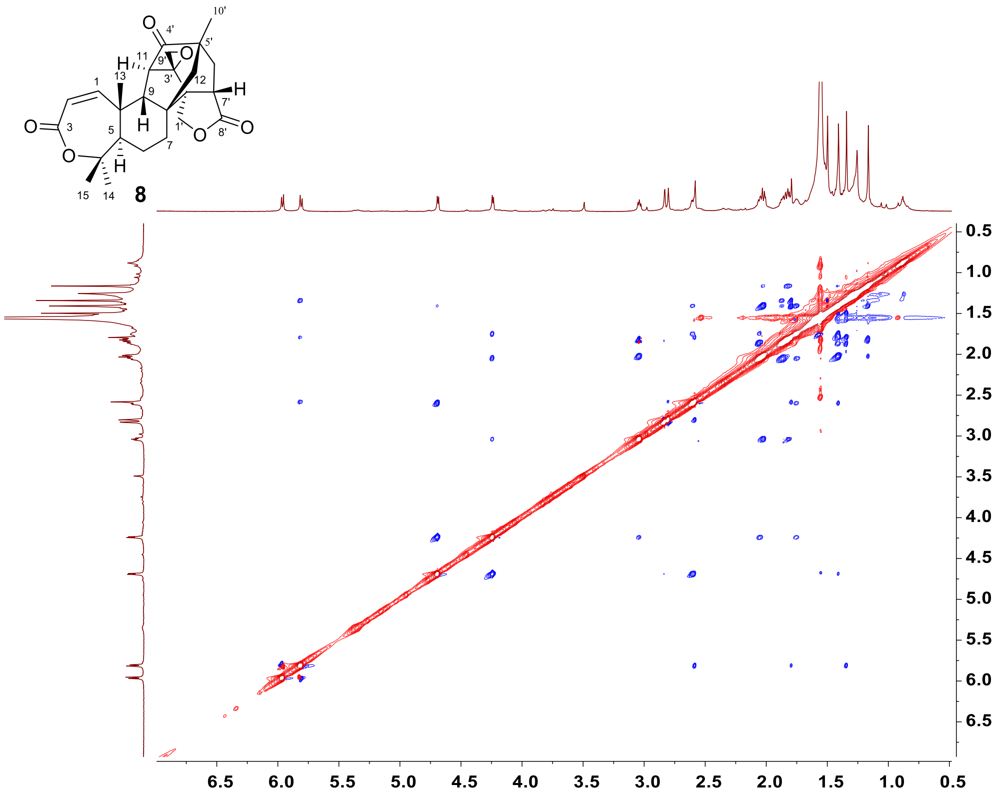

Supplementary Fig. 36 | NOESY NMR spectrum of 8 in CDCl3.

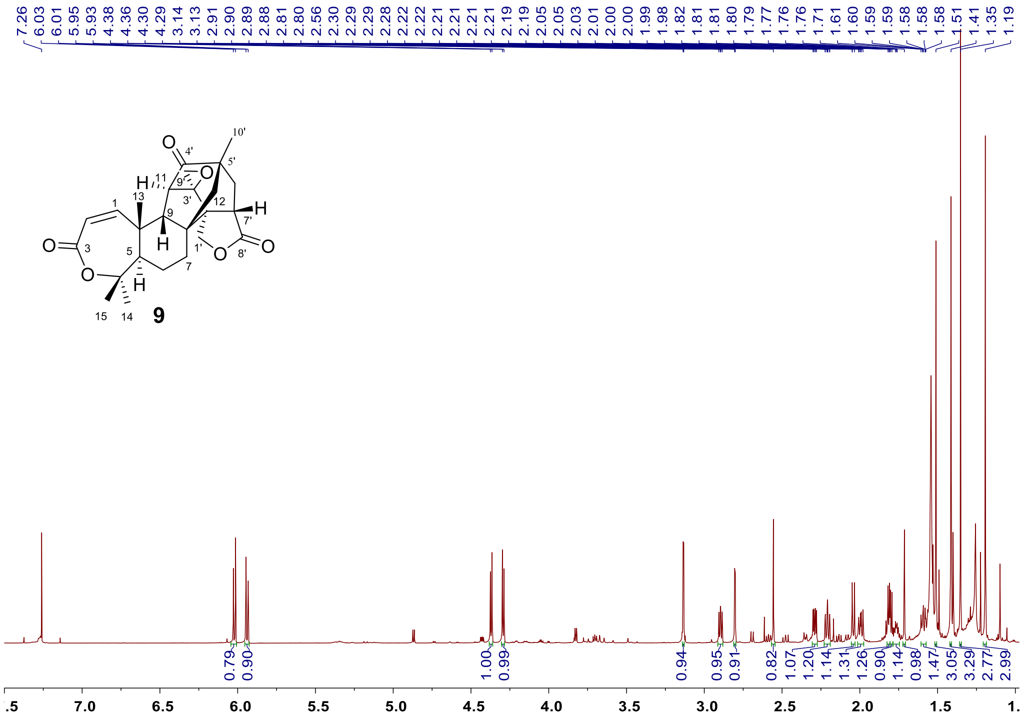

Supplementary Fig. 37 | ^{1}H NMR spectrum of **9** in $CDCl_3$.

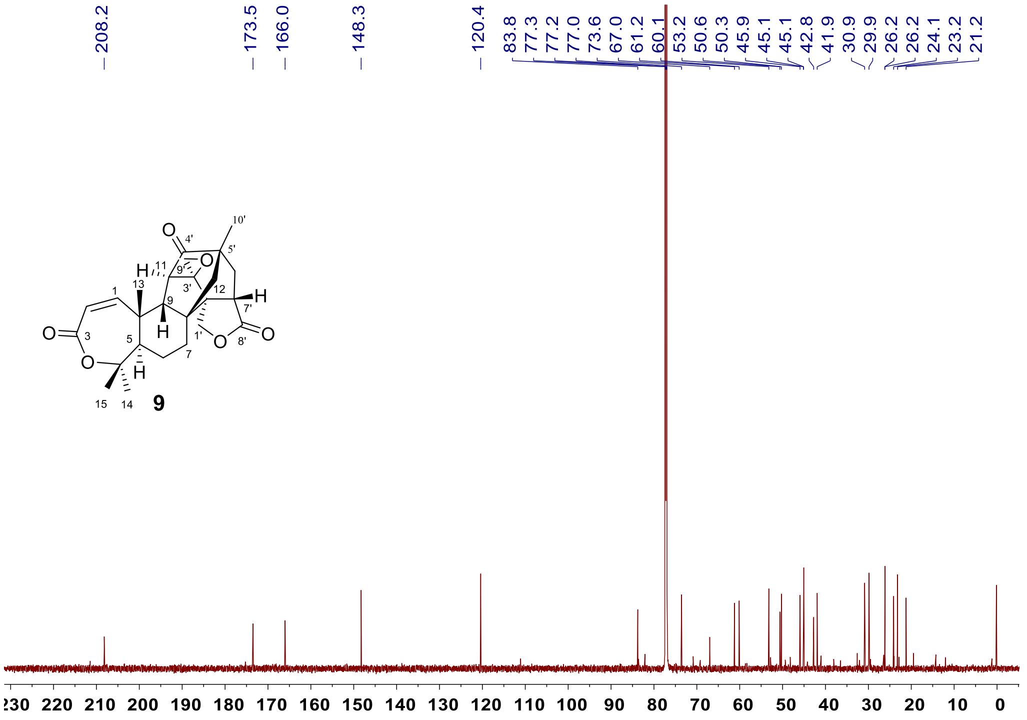

Supplementary Fig. 38 | ^{13}C NMR spectrum of **9** in $CDCl_3$.

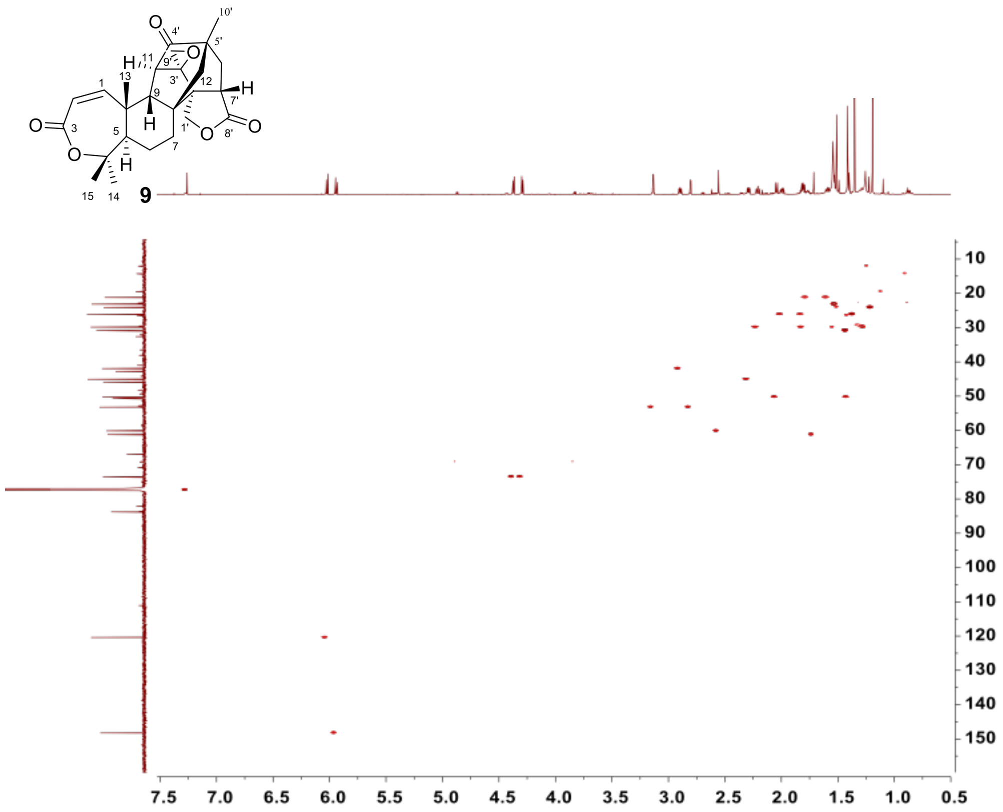

Supplementary Fig. 39 | ** HSQC NMR spectrum of **9 in $CDCl_3$.

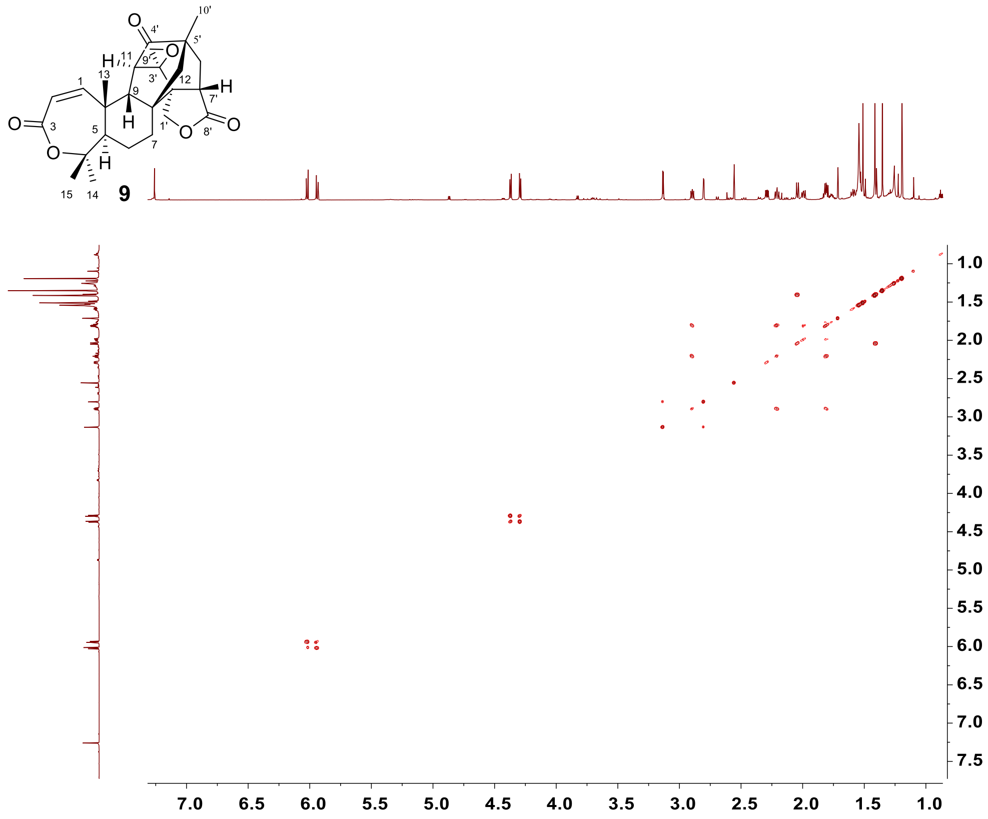

Supplementary Fig. 40 | ** COSY NMR spectrum of **9 in $CDCl_3$.

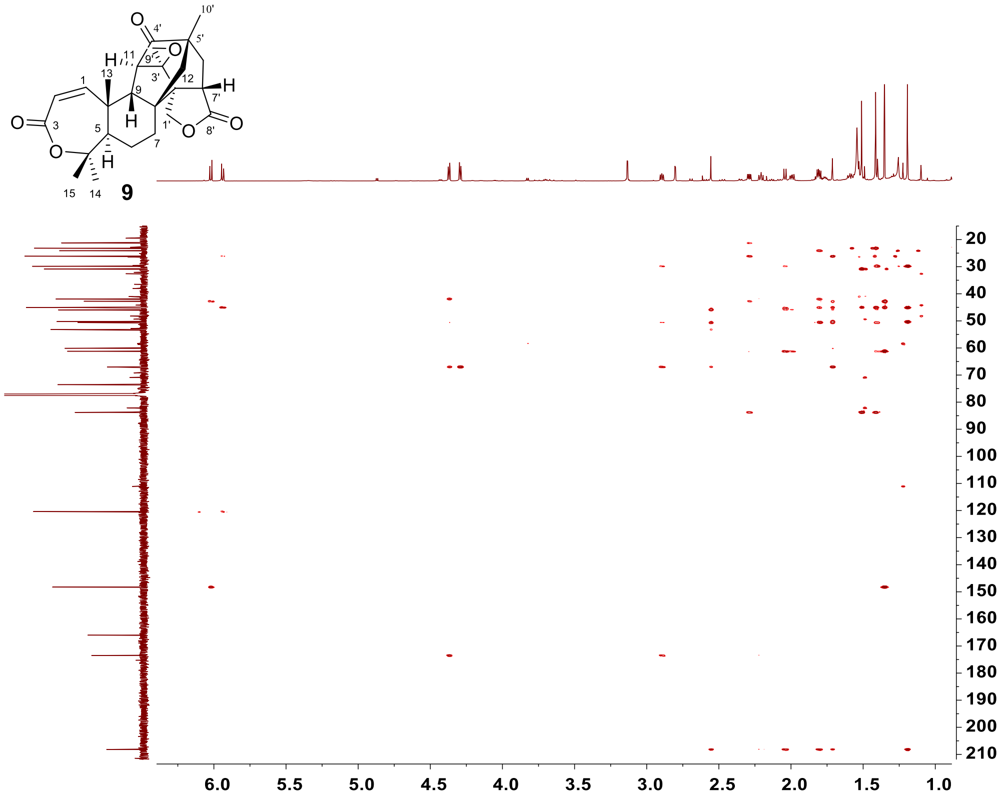

Supplementary Fig. 41 | ** HMBC NMR spectrum of **9 in CDCl3.

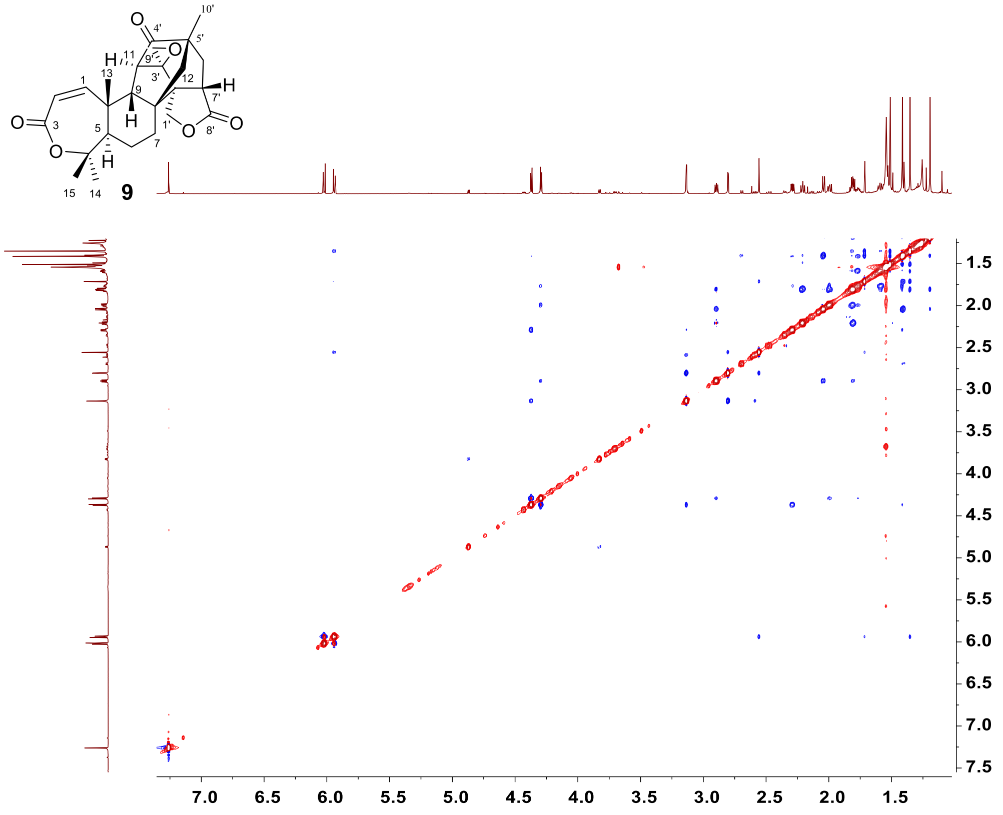

Supplementary Fig. 42 | ** NOESY NMR spectrum of **9 in CDCl3.

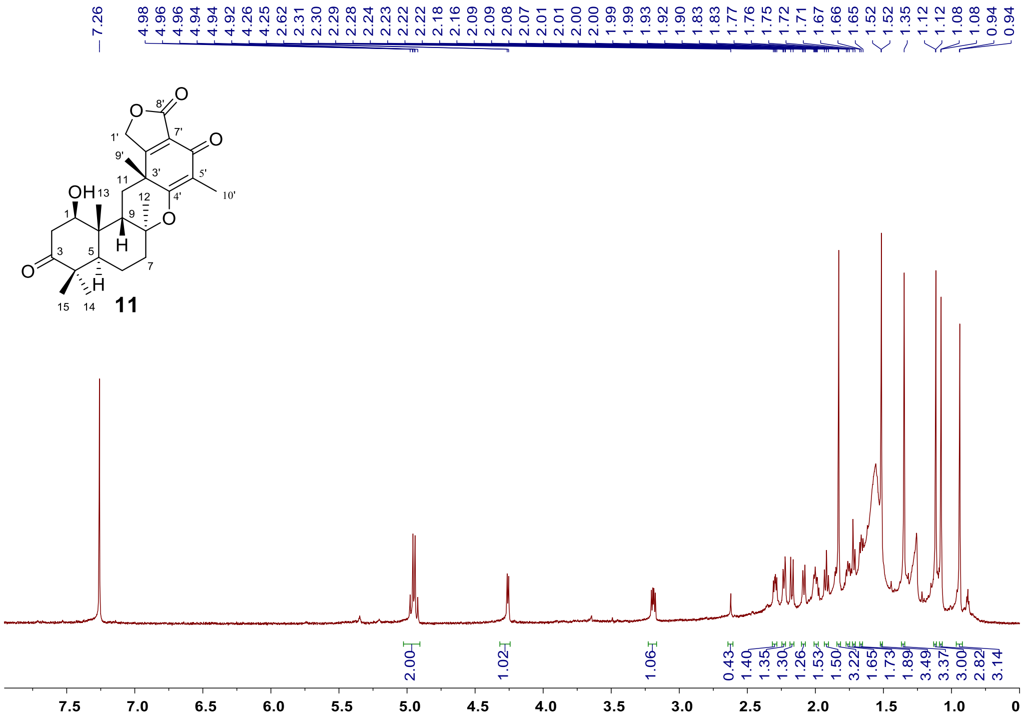

Supplementary Fig. 43 | **^{1}H NMR spectrum of **11 in CDCl3.

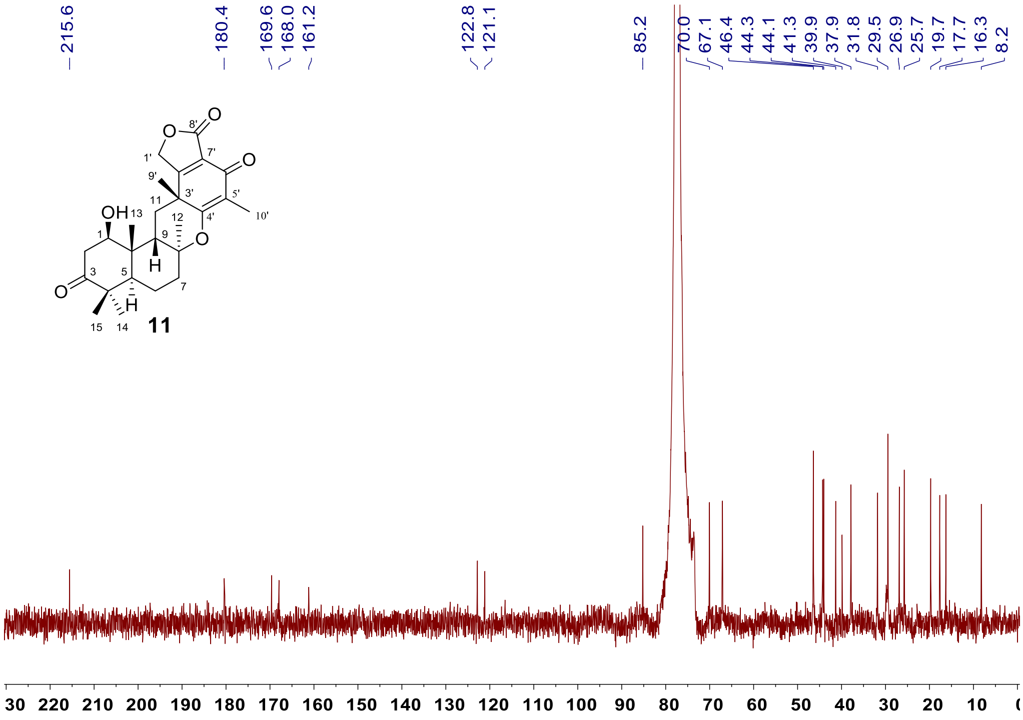

Supplementary Fig. 44 | **^{13}C NMR spectrum of **11 in CDCl3.

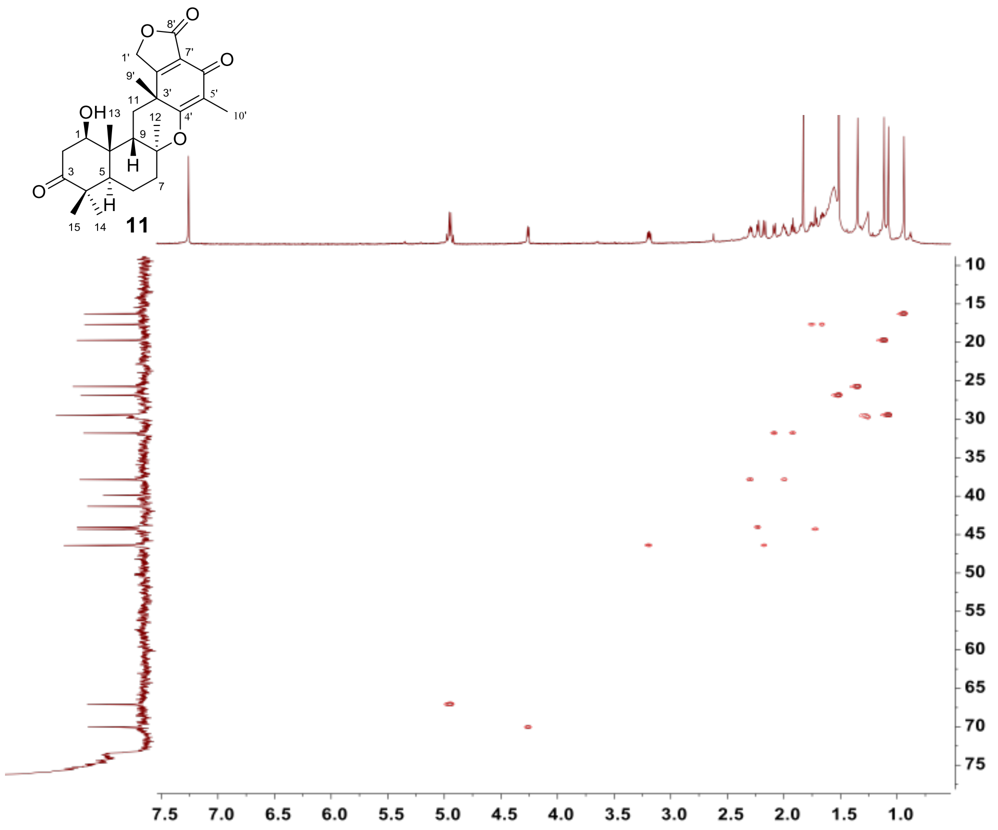

Supplementary Fig. 45 | ** HSQC NMR spectrum of **11 in CDCl3.

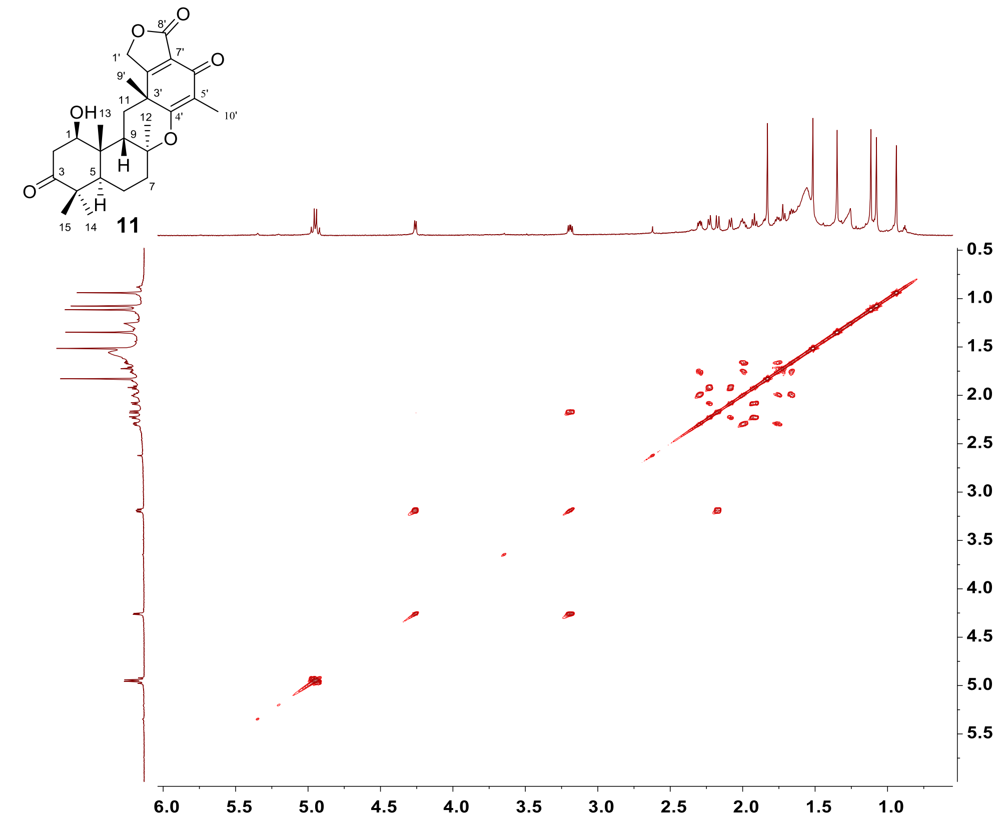

Supplementary Fig. 46 | ** COSY NMR spectrum of **11 in CDCl3.

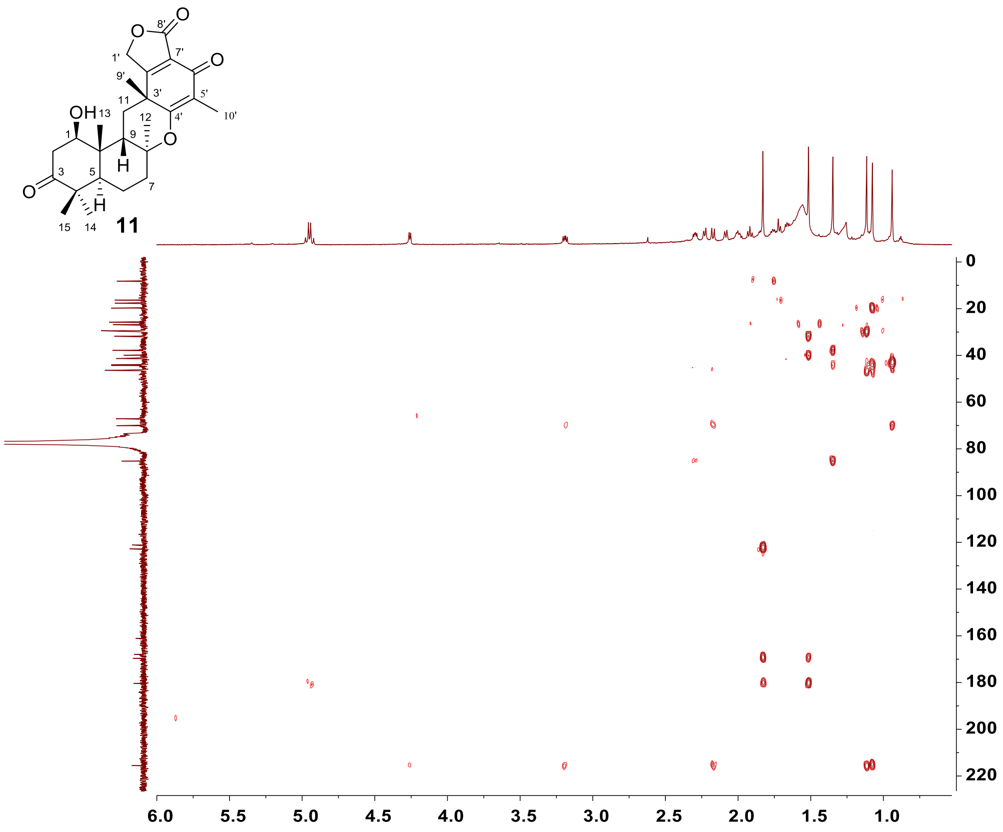

Supplementary Fig. 47 | HMBC NMR spectrum of **11** in $CDCl_3$.

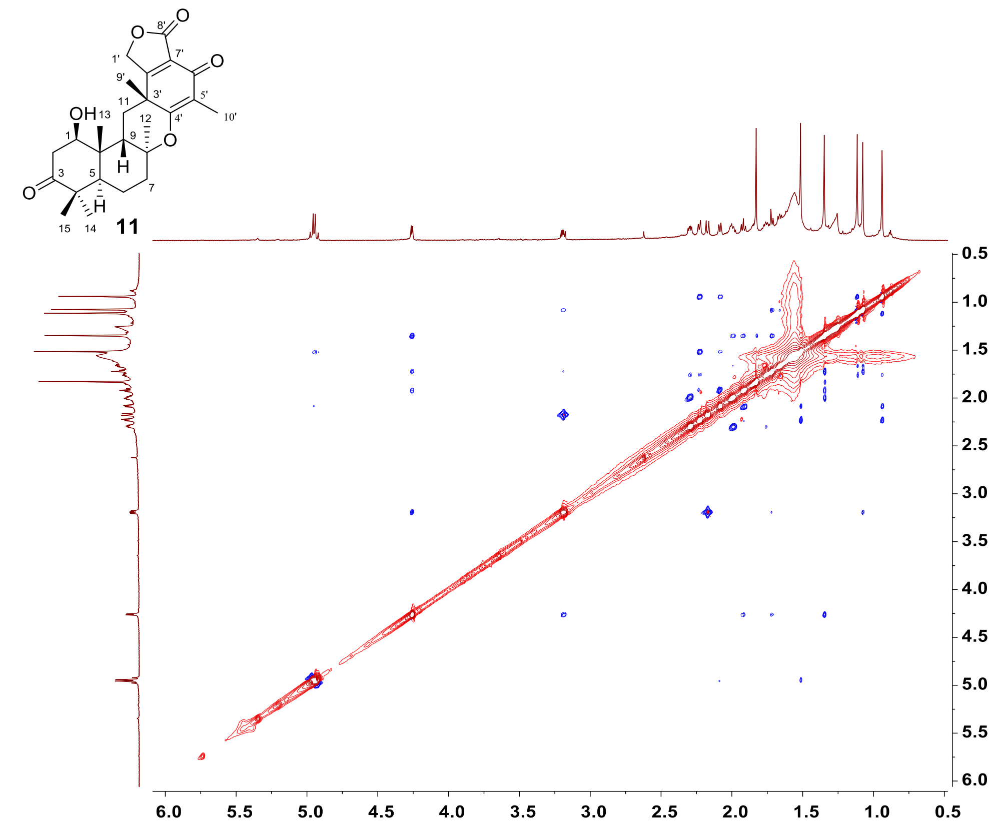

Supplementary Fig. 48 | NOESY NMR spectrum of **11** in $CDCl_3$.

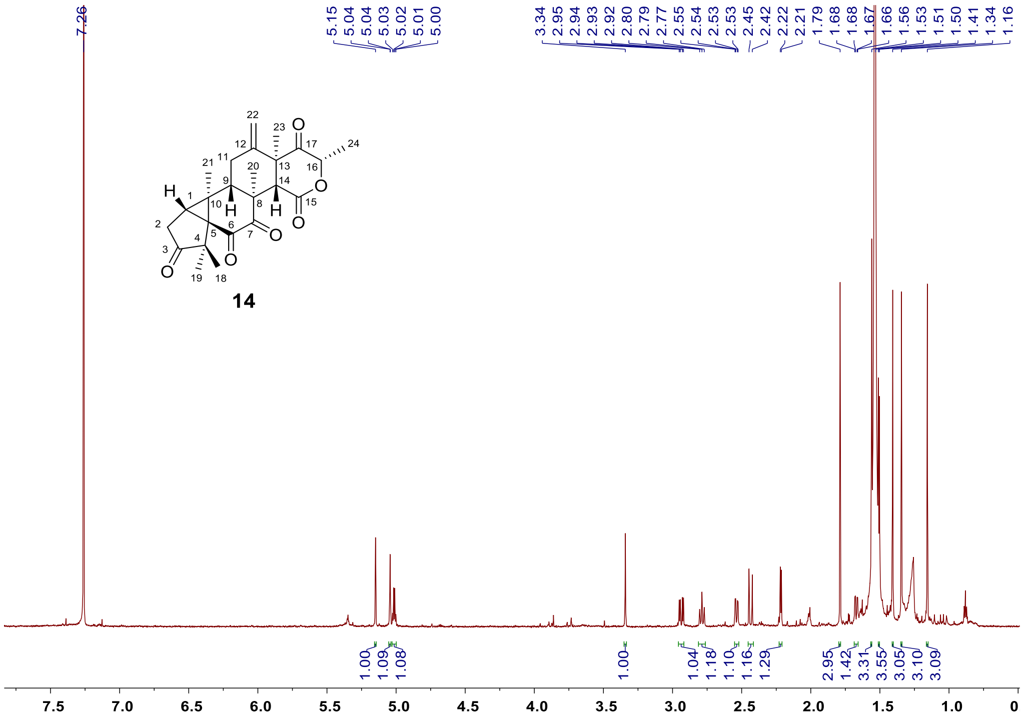

Supplementary Fig. 49 | ^{1}H NMR spectrum of **14** in $CDCl_3$.

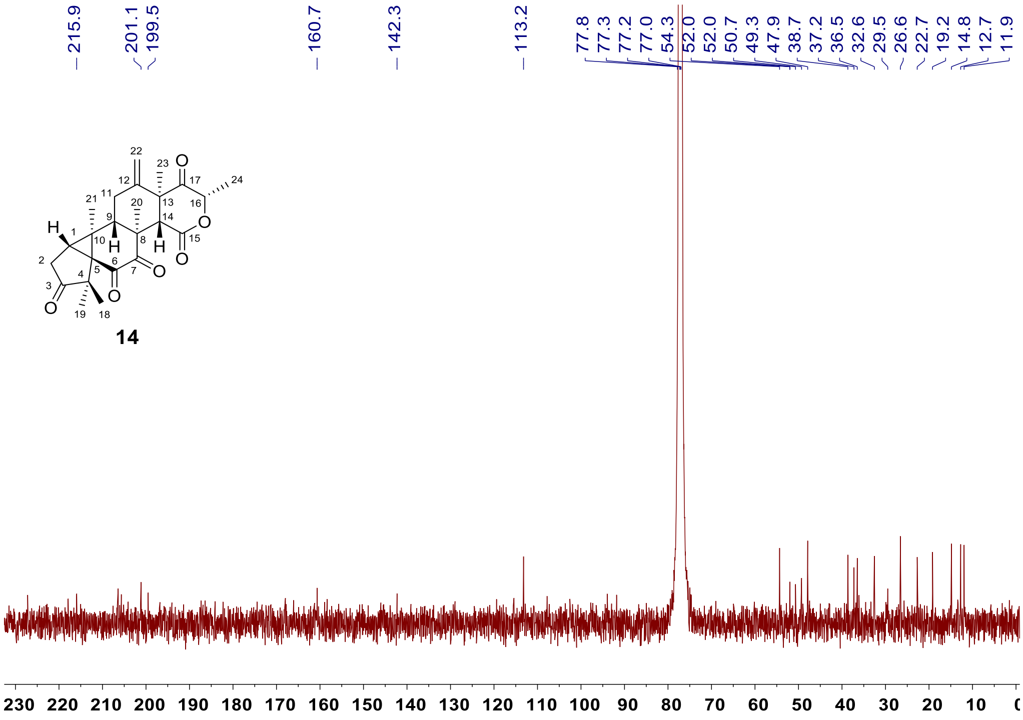

Supplementary Fig. 50 | ^{13}C NMR spectrum of **14** in $CDCl_3$.

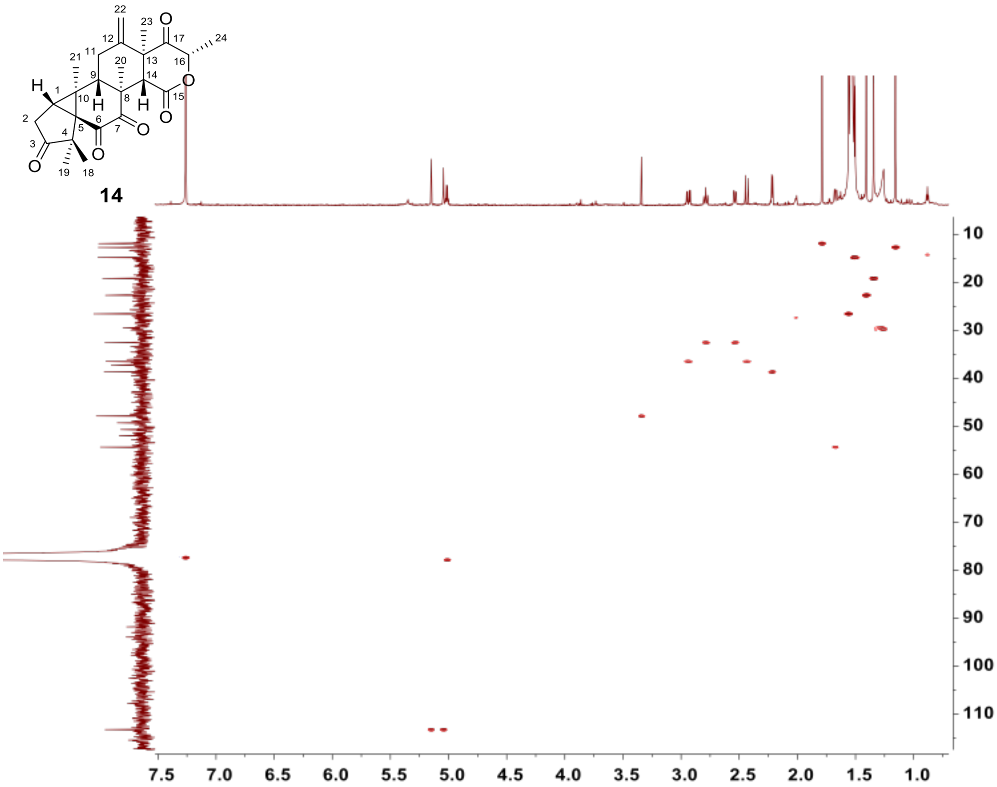

Supplementary Fig. 51 | HSQC NMR spectrum of 14 in CDCl3.

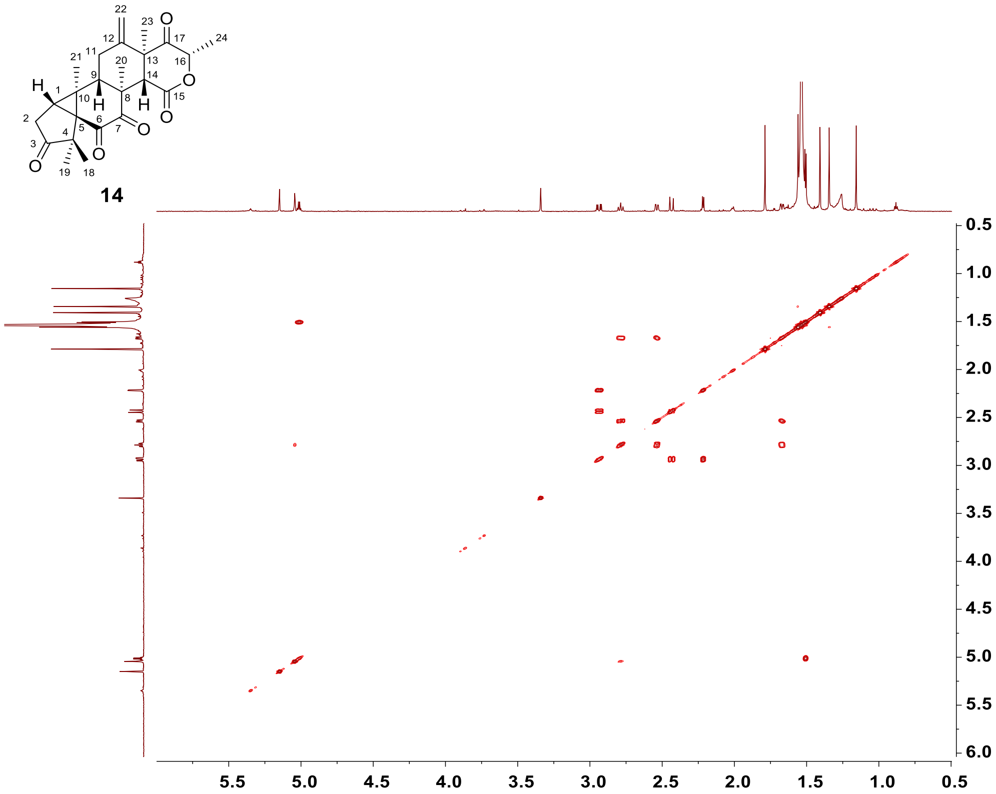

Supplementary Fig. 52 | COSY NMR spectrum of 14 in CDCl3.

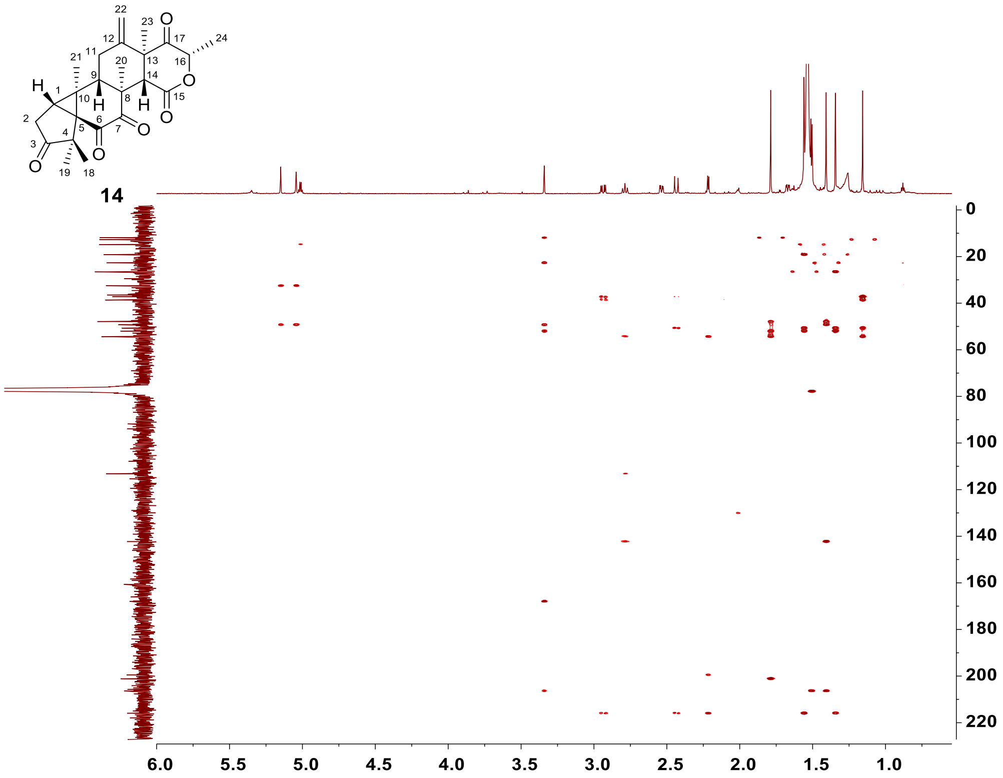

Supplementary Fig. 53 | HMBC NMR spectrum of 14 in $CDCl_3$.

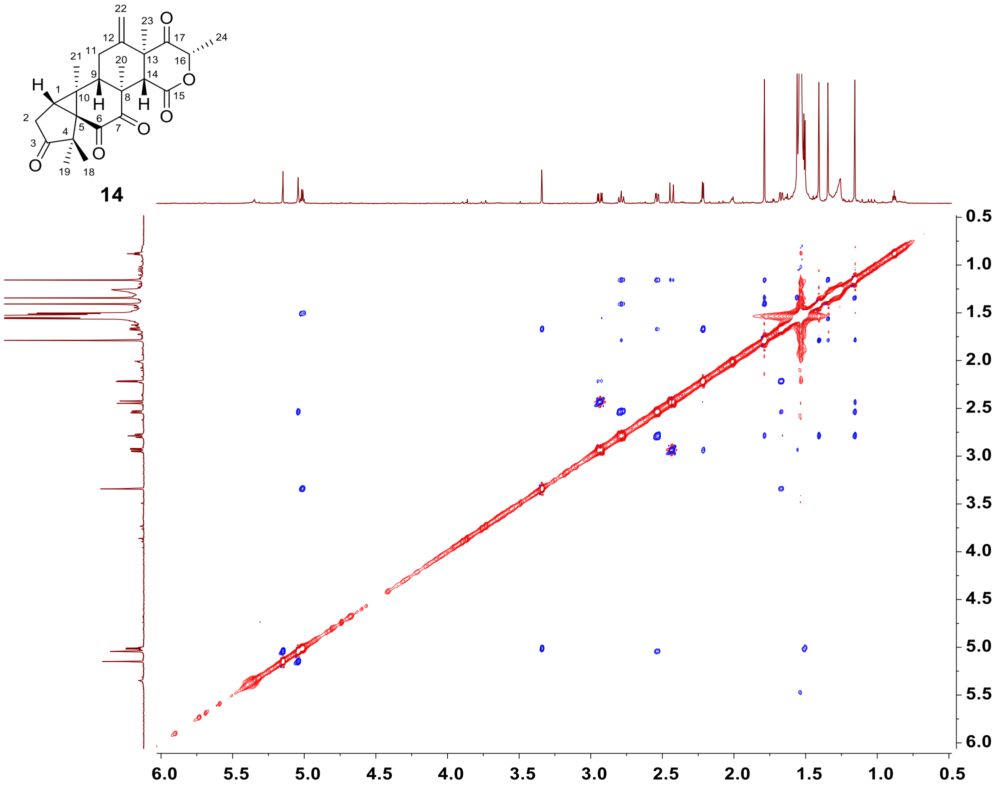

Supplementary Fig. 54 | NOESY NMR spectrum of 14 in $CDCl_3$.

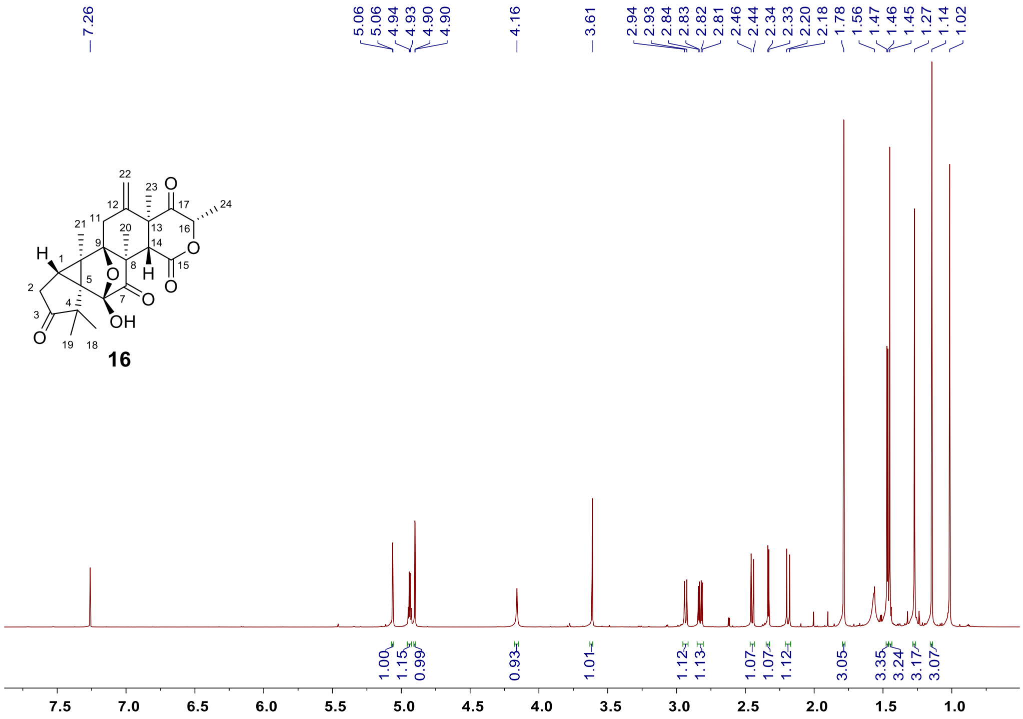

Supplementary Fig. 55 | ^{1}H NMR spectrum of **16** in $CDCl_3$.

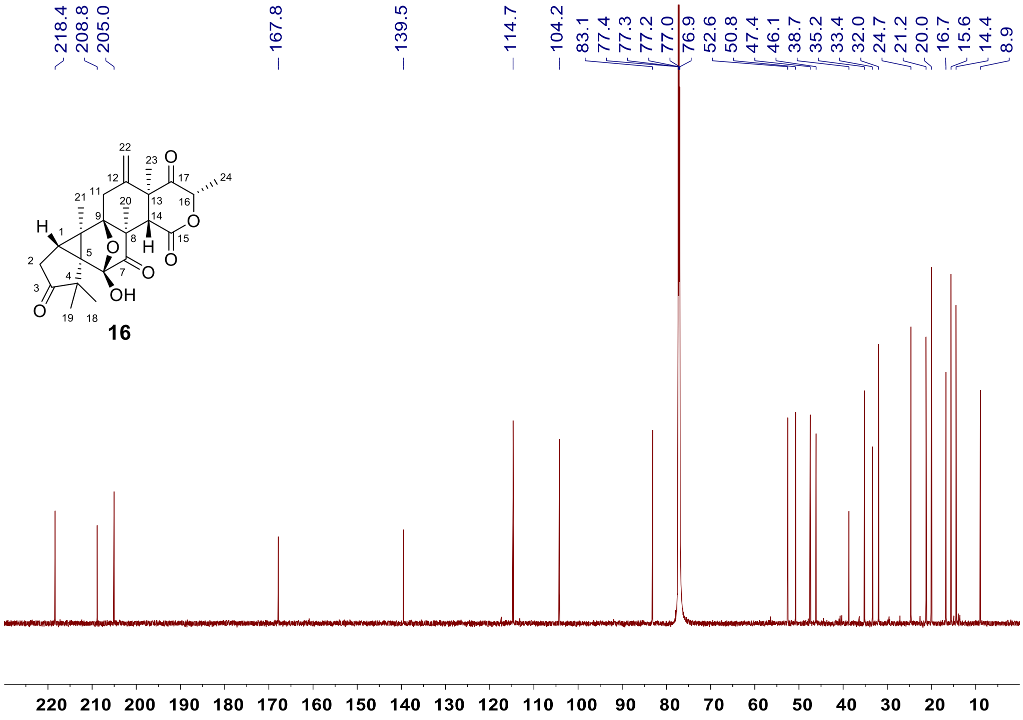

Supplementary Fig. 56 | ^{13}C NMR spectrum of **16** in $CDCl_3$.

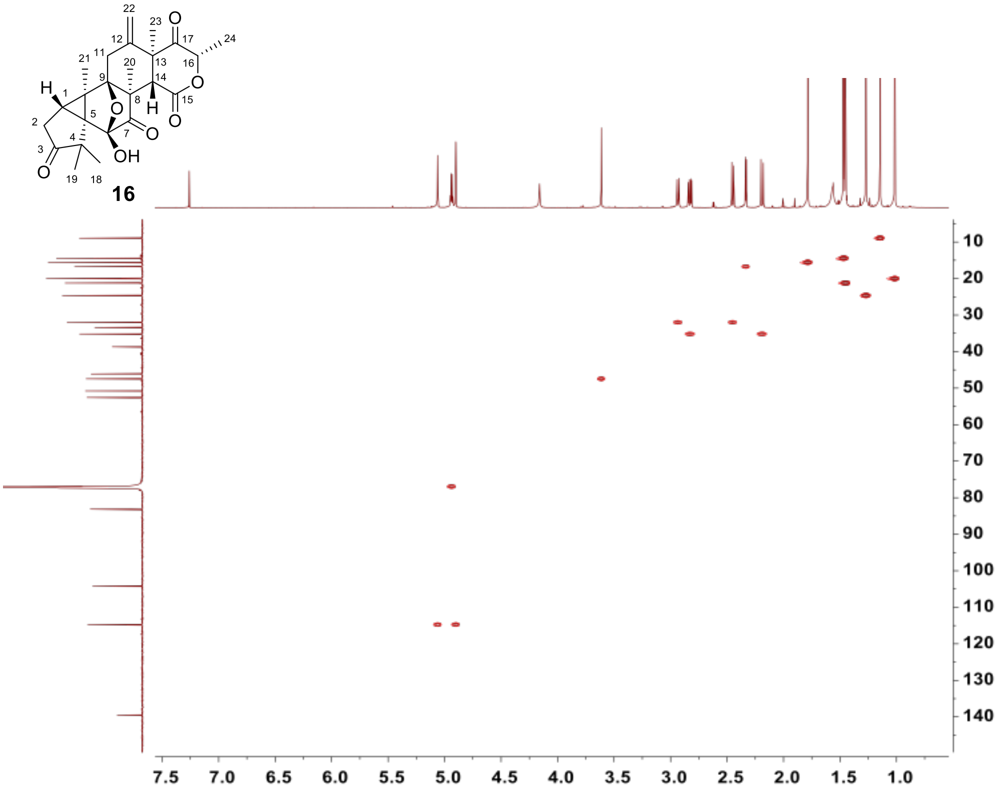

Supplementary Fig. 57 | HSQC NMR spectrum of **16** in $CDCl_3$.

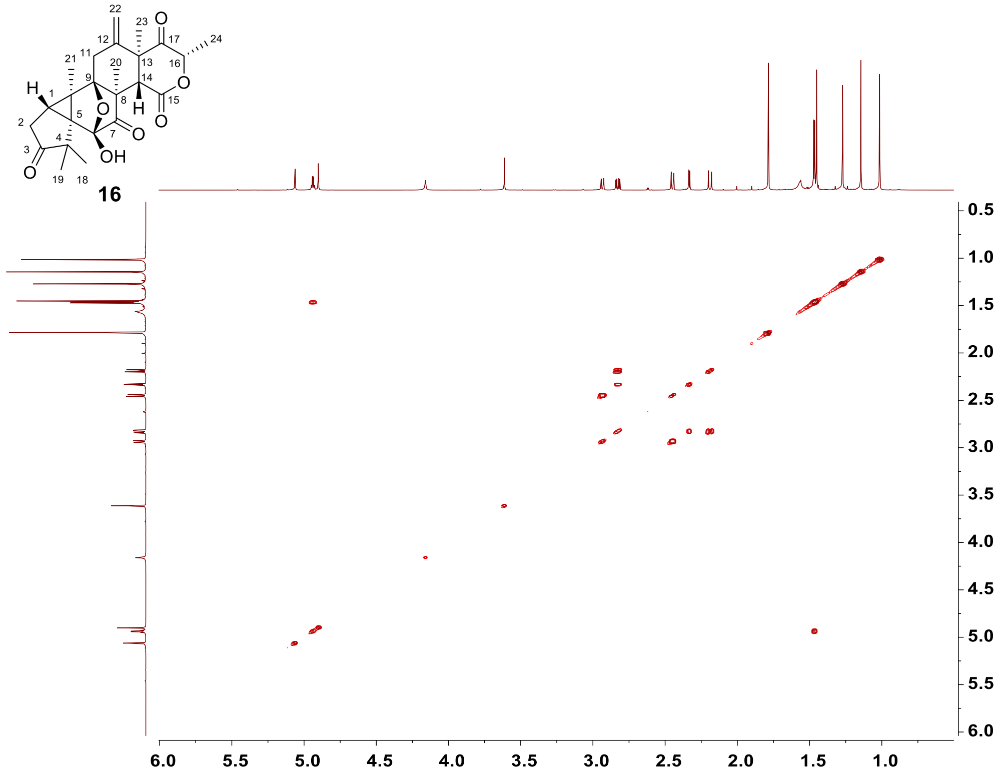

Supplementary Fig. 58 | COSY NMR spectrum of **16** in $CDCl_3$.

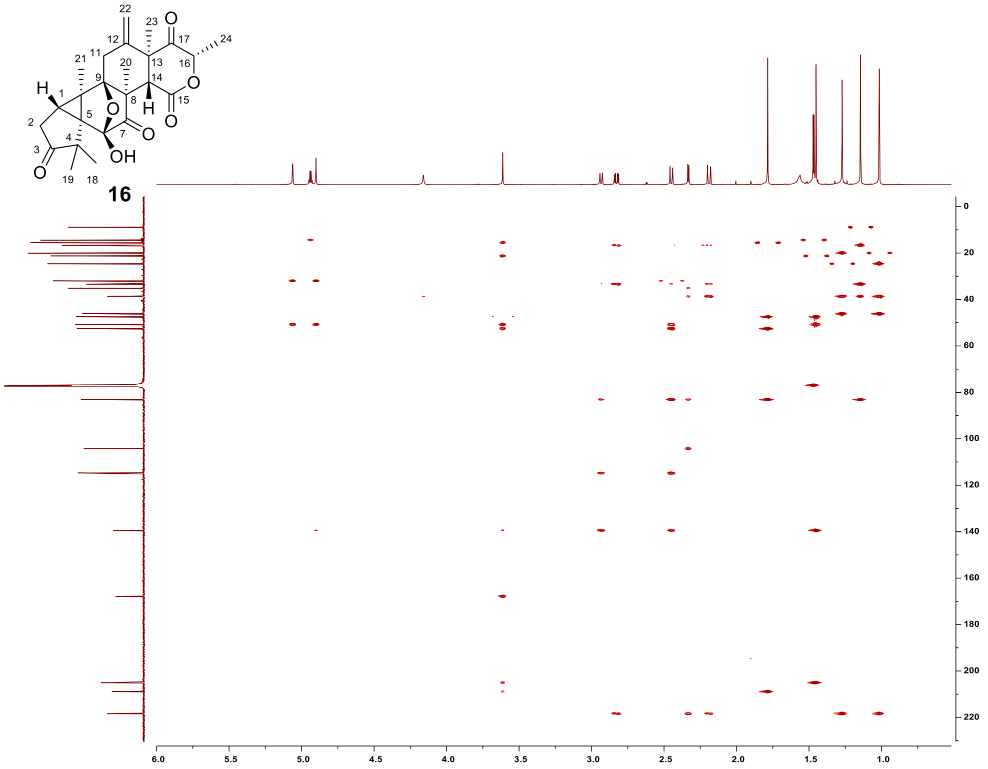

Supplementary Fig. 59 | ** HMBC NMR spectrum of **16 in $CDCl_3$.

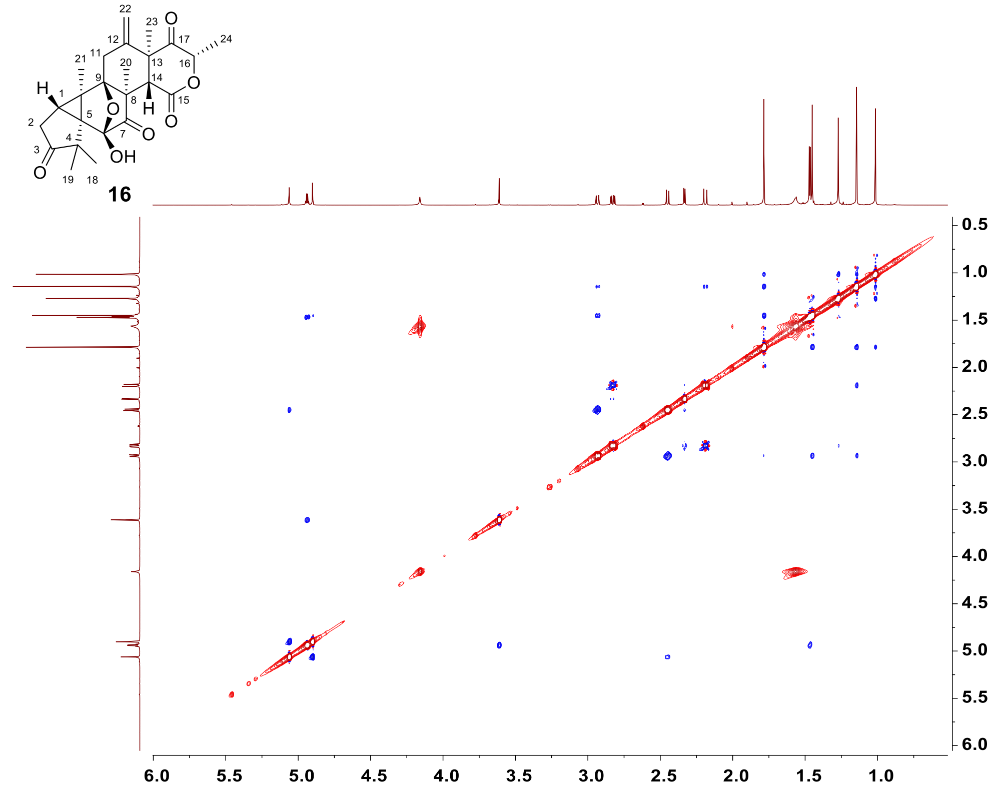

Supplementary Fig. 60 | ** NOESY NMR spectrum of **16 in $CDCl_3$.

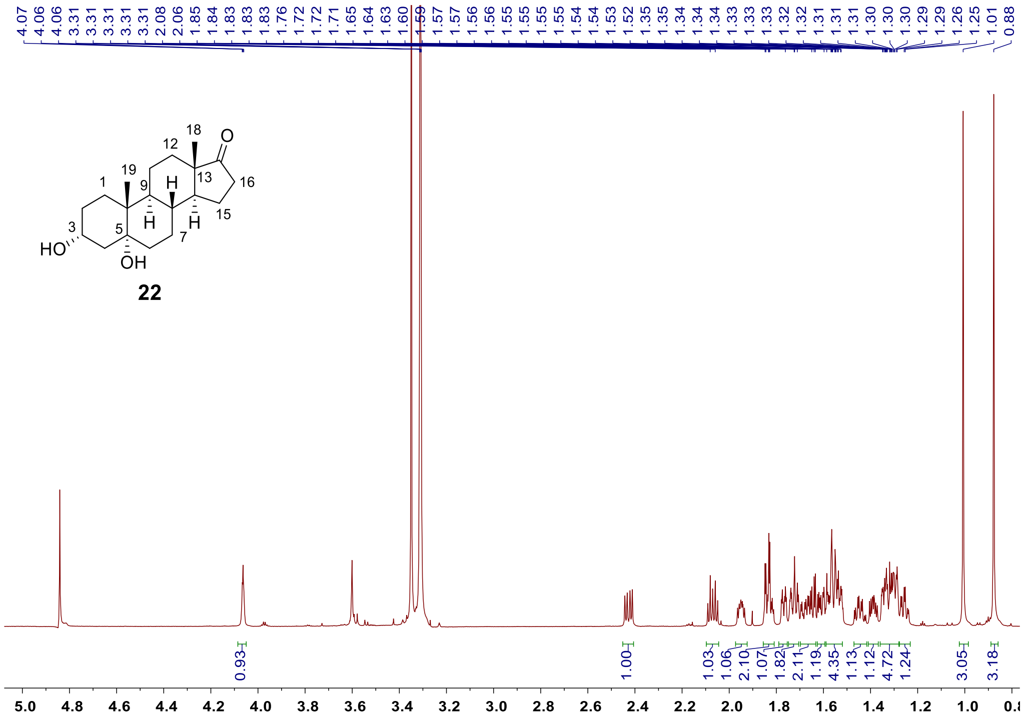

Supplementary Fig. 61 | **^{1}H NMR spectrum of **22 in CD_3OD.

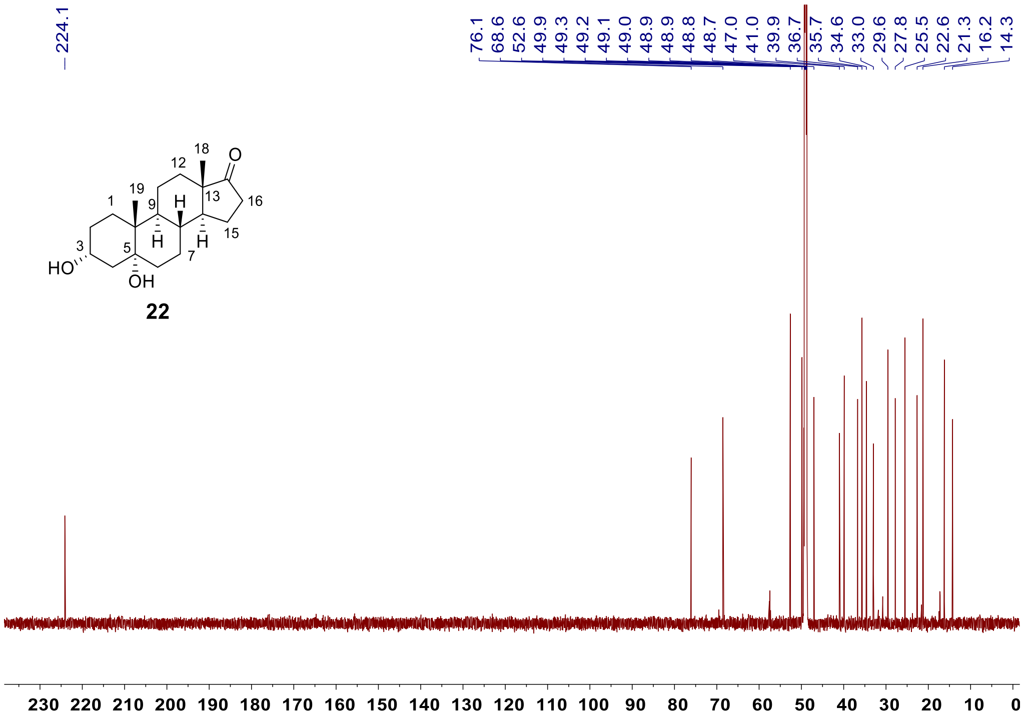

Supplementary Fig. 62 | **^{13}C NMR spectrum of **22 in CD_3OD.

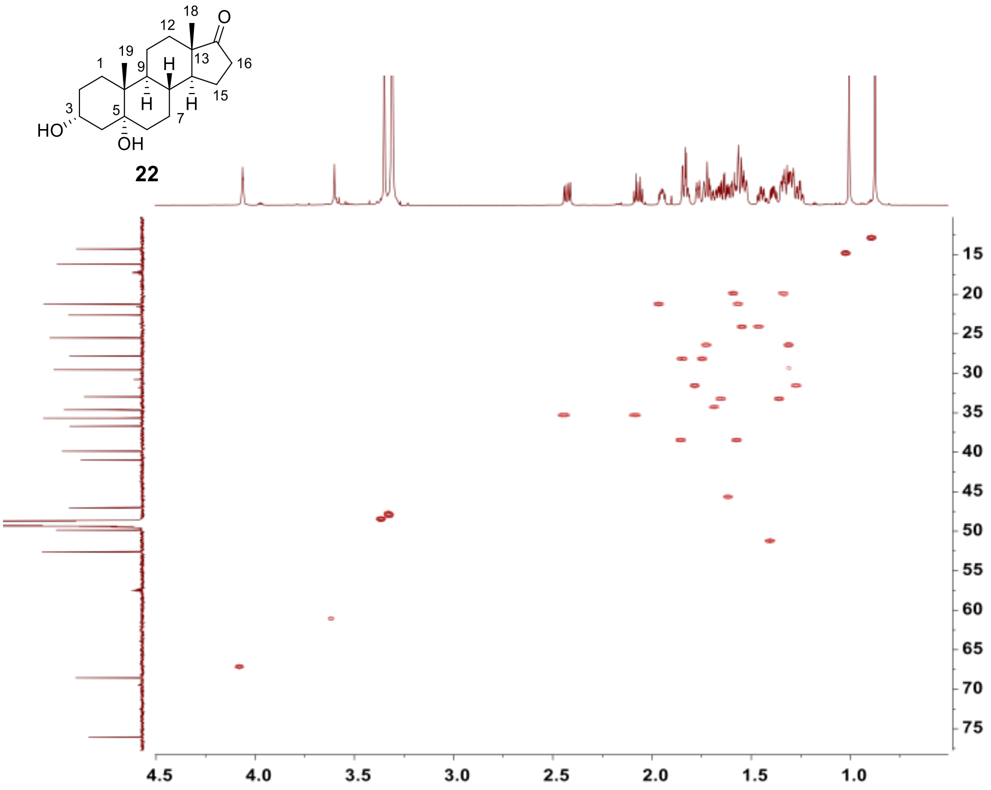

Supplementary Fig. 63 | ** HSQC spectrum of **22 in CD_3OD.

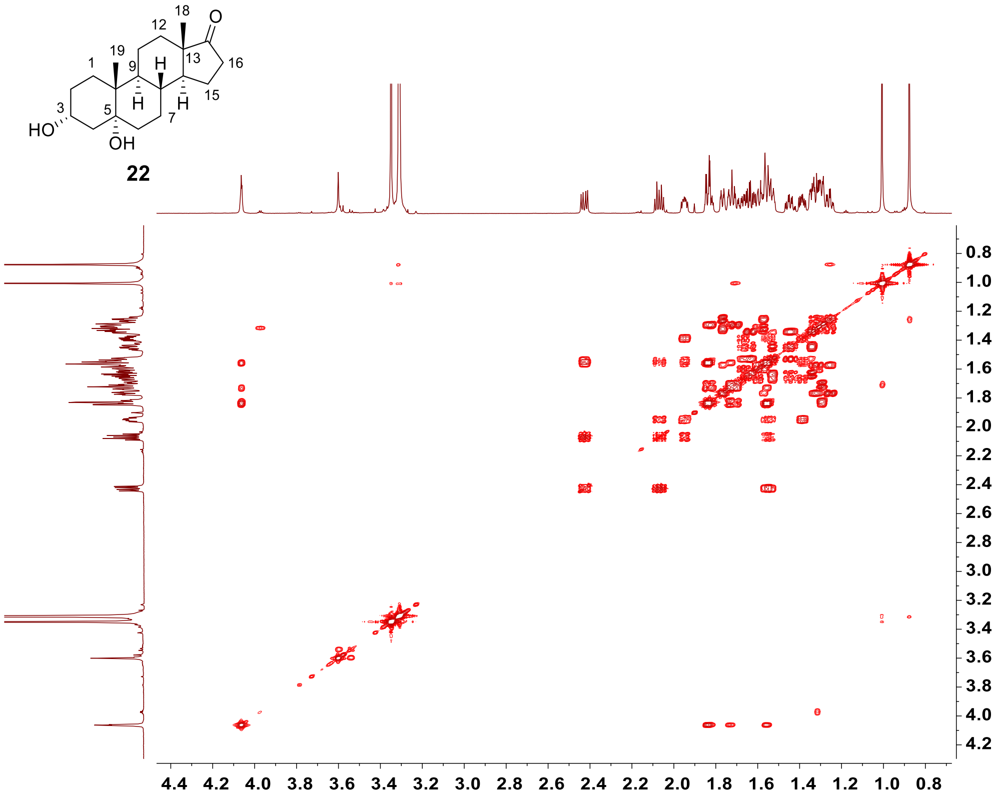

Supplementary Fig. 64 | ** COSY spectrum of **22 in CD_3OD.

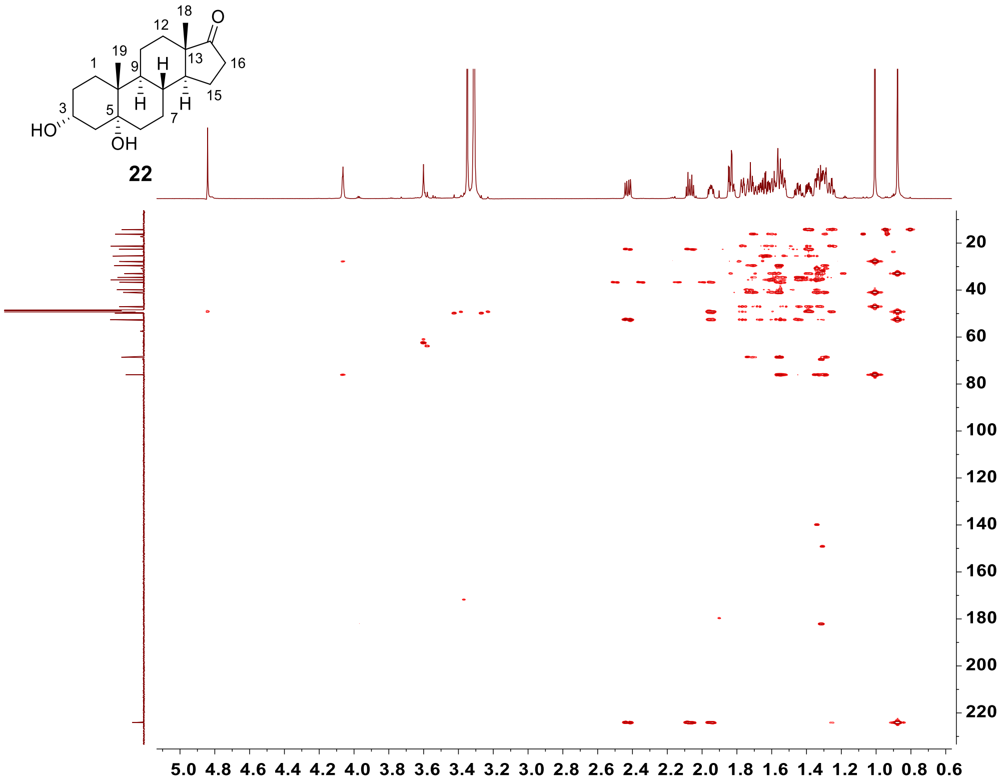

Supplementary Fig. 65 | ** HMBC NMR spectrum of **22 in CD_3OD.

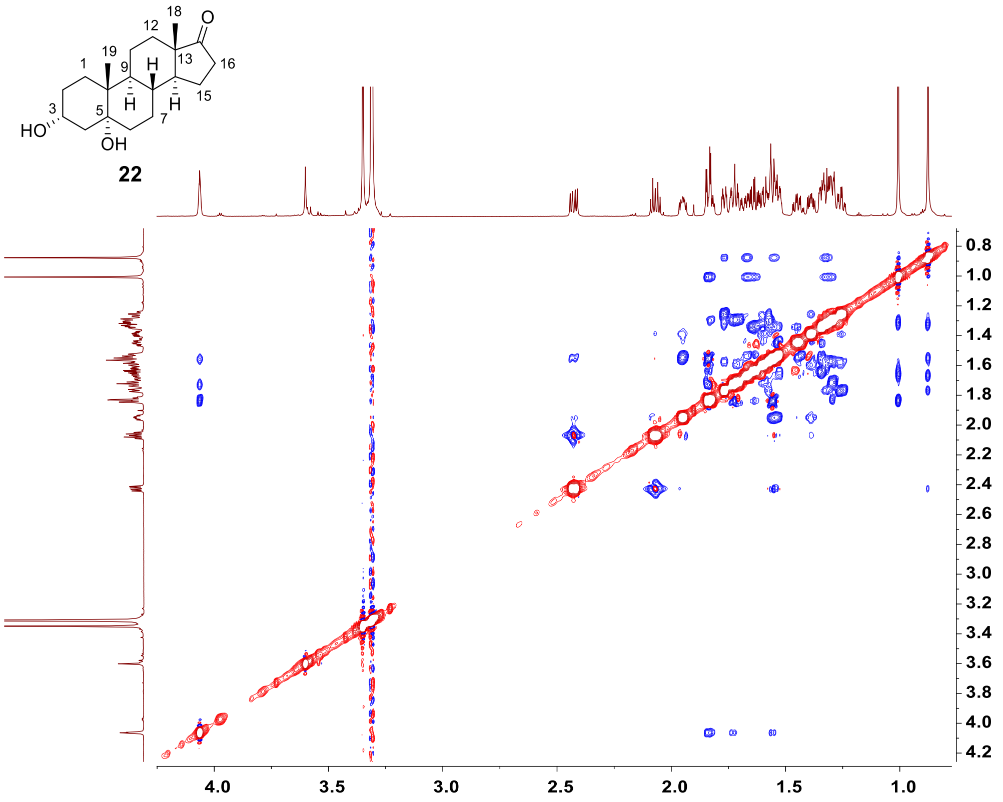

Supplementary Fig. 66 | ** NOESY NMR spectrum of **22 in CD_3OD.

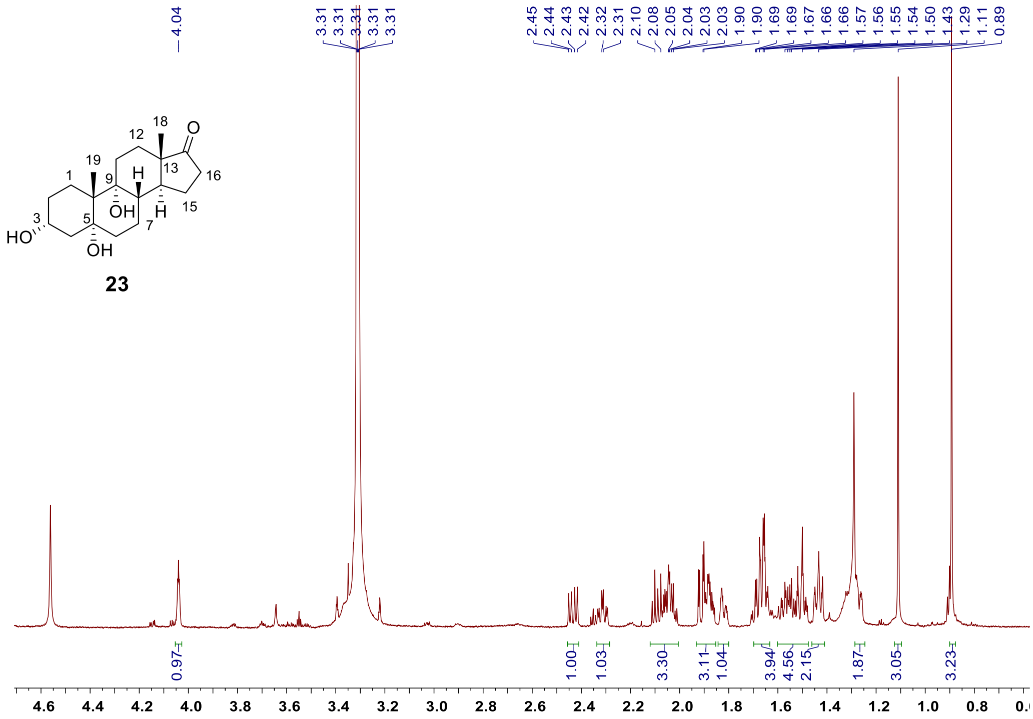

Supplementary Fig. 67 | **^{1}H NMR spectrum of **23 in CD_3OD.

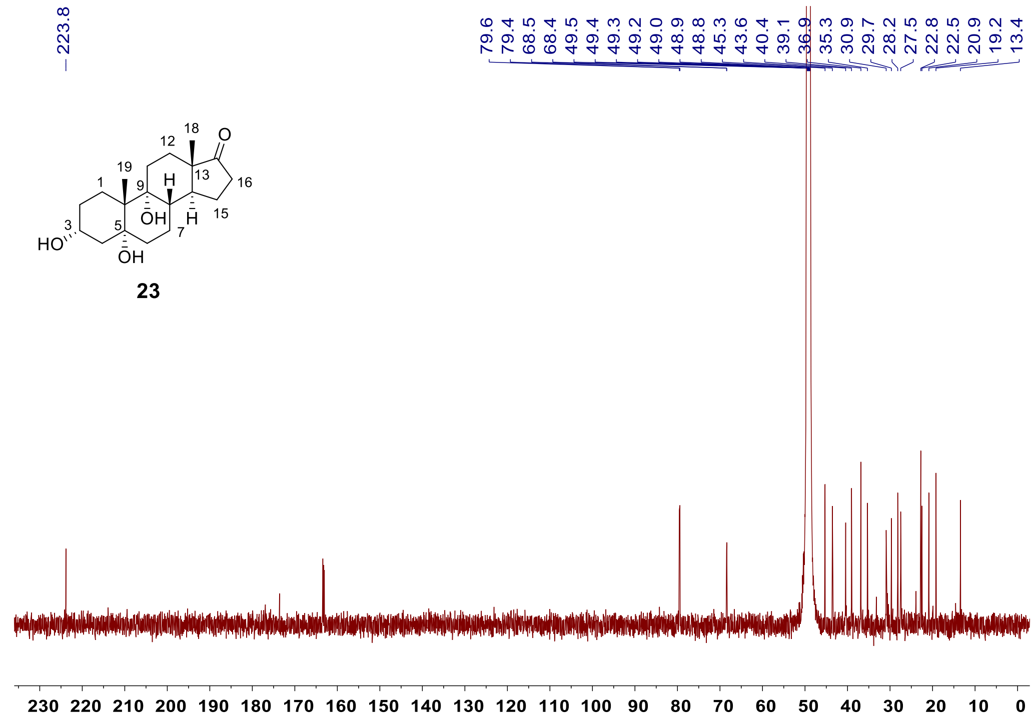

Supplementary Fig. 68 | **^{13}C NMR spectrum of **23 in CD_3OD.

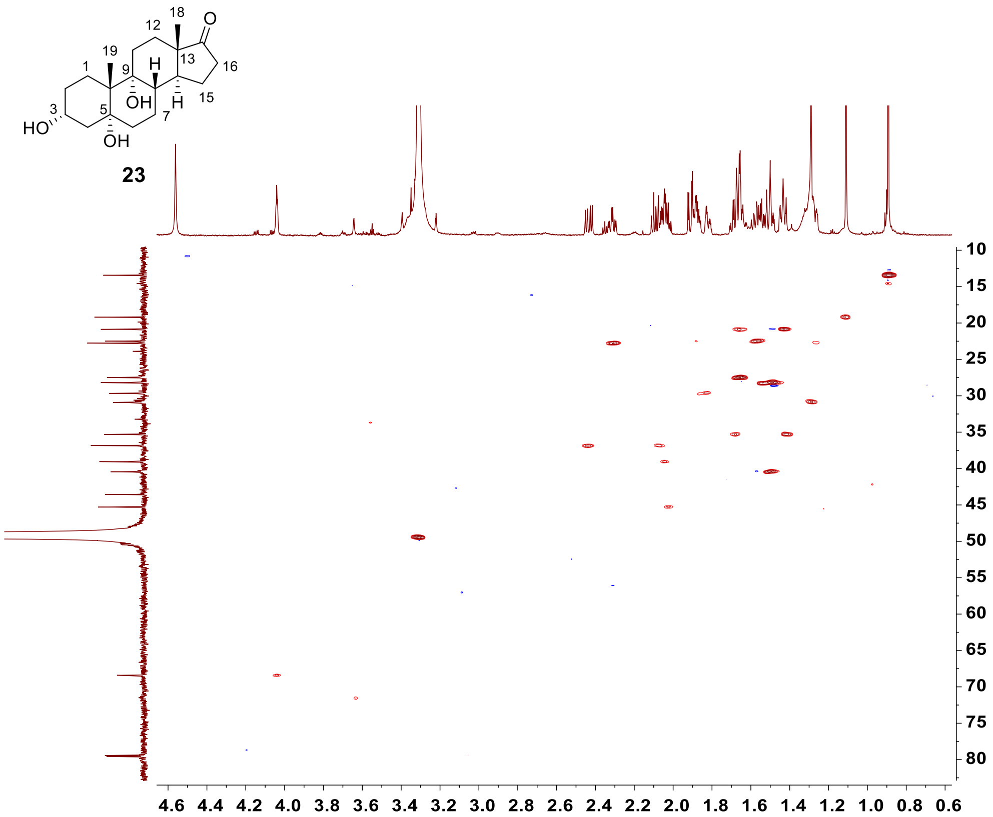

Supplementary Fig. 69 | ** HSQC spectrum of **23 in CD_3OD.

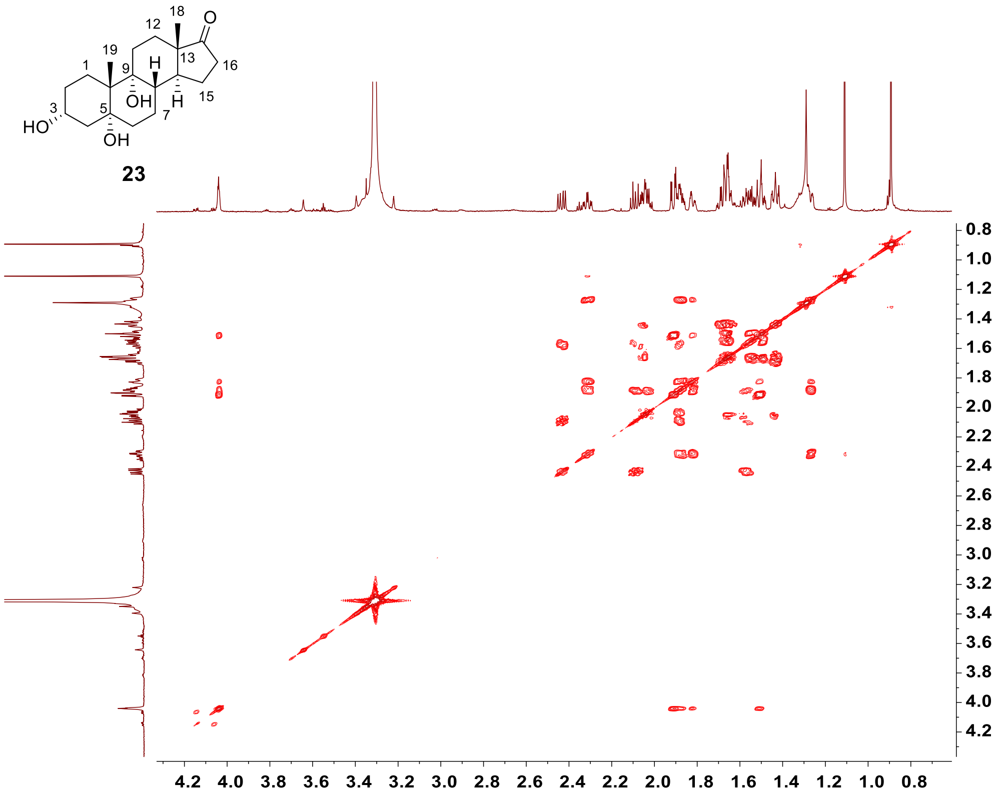

Supplementary Fig. 70 | ** COSY spectrum of **23 in CD_3OD.

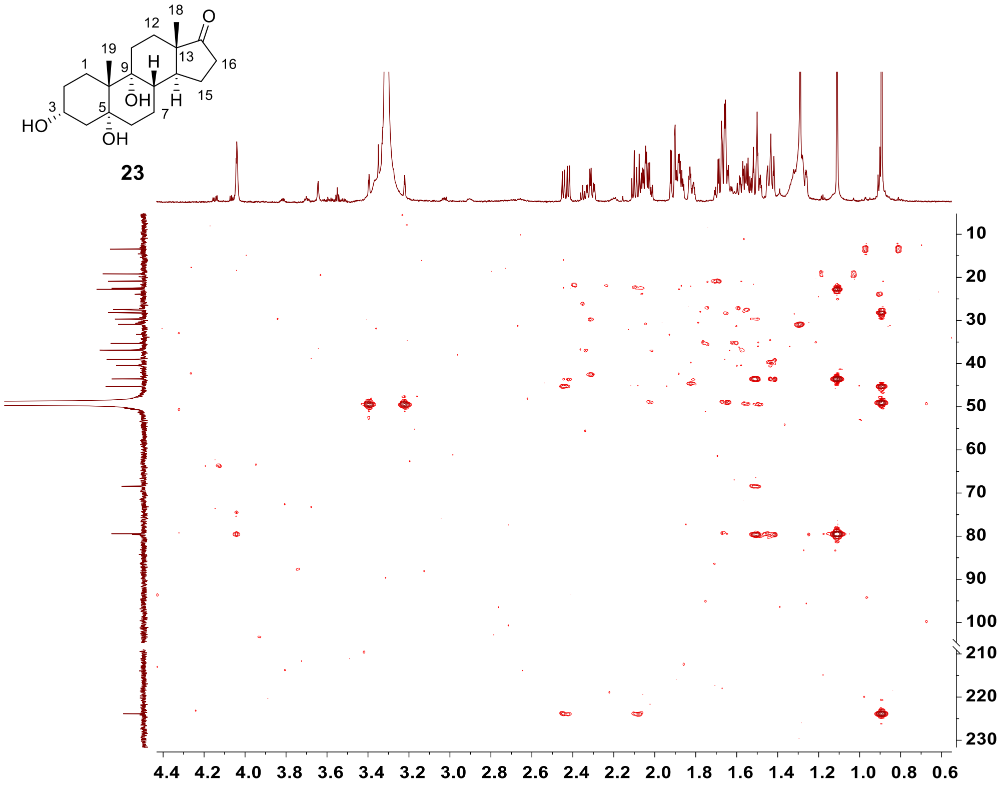

Supplementary Fig. 71 | HMBC spectrum of **23** in CD_3OD.

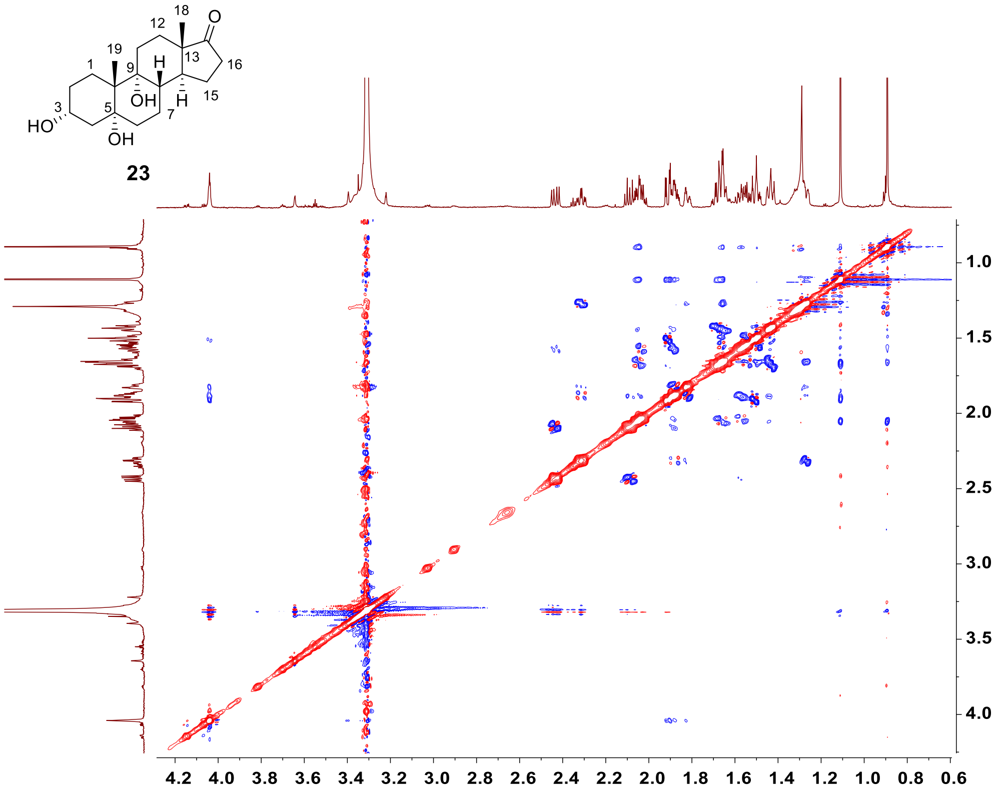

Supplementary Fig. 72 | NOESY spectrum of **23** in CD_3OD.

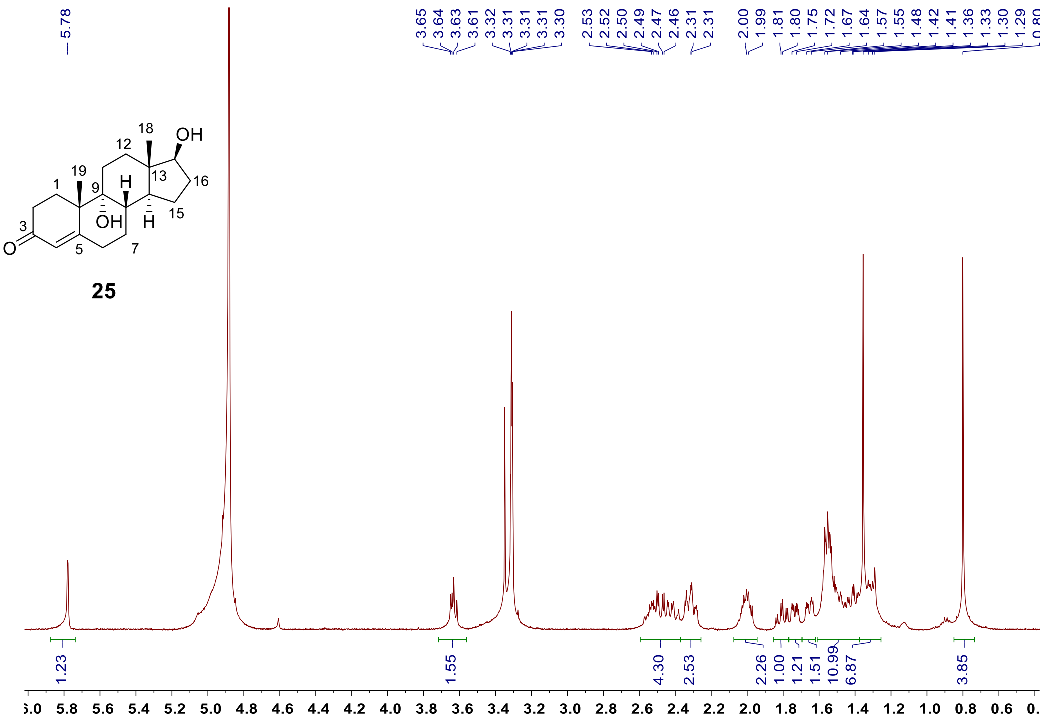

Supplementary Fig. 73 | **^{1}H NMR spectrum of **25 in CD_3OD.

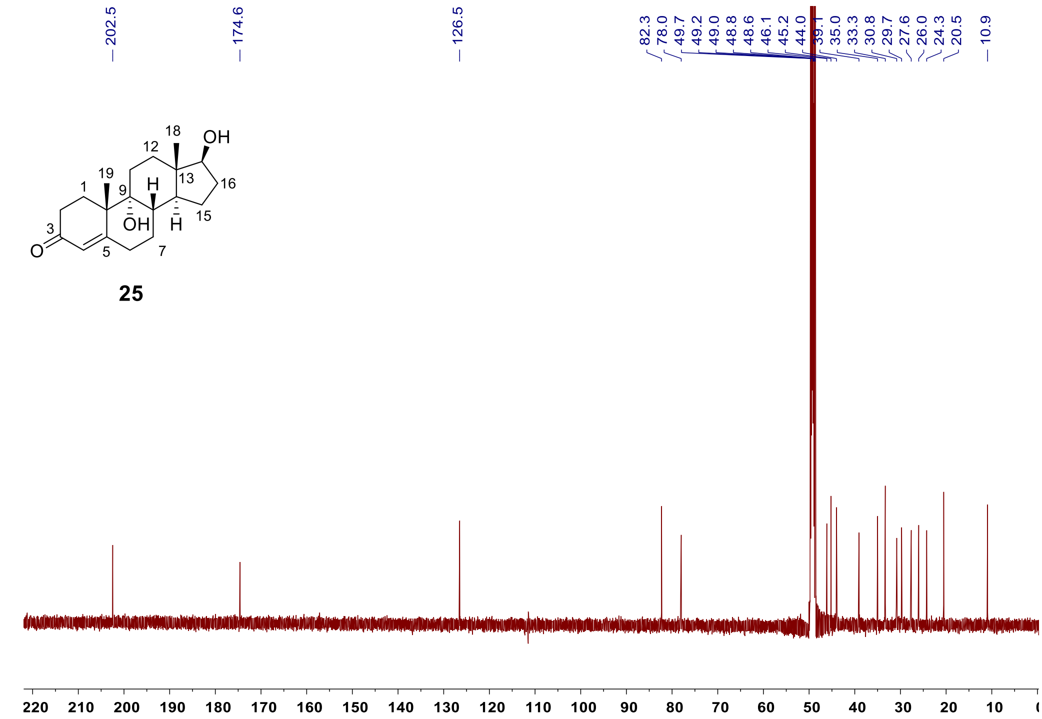

Supplementary Fig. 74 | **^{13}C NMR spectrum of **25 in CD_3OD.

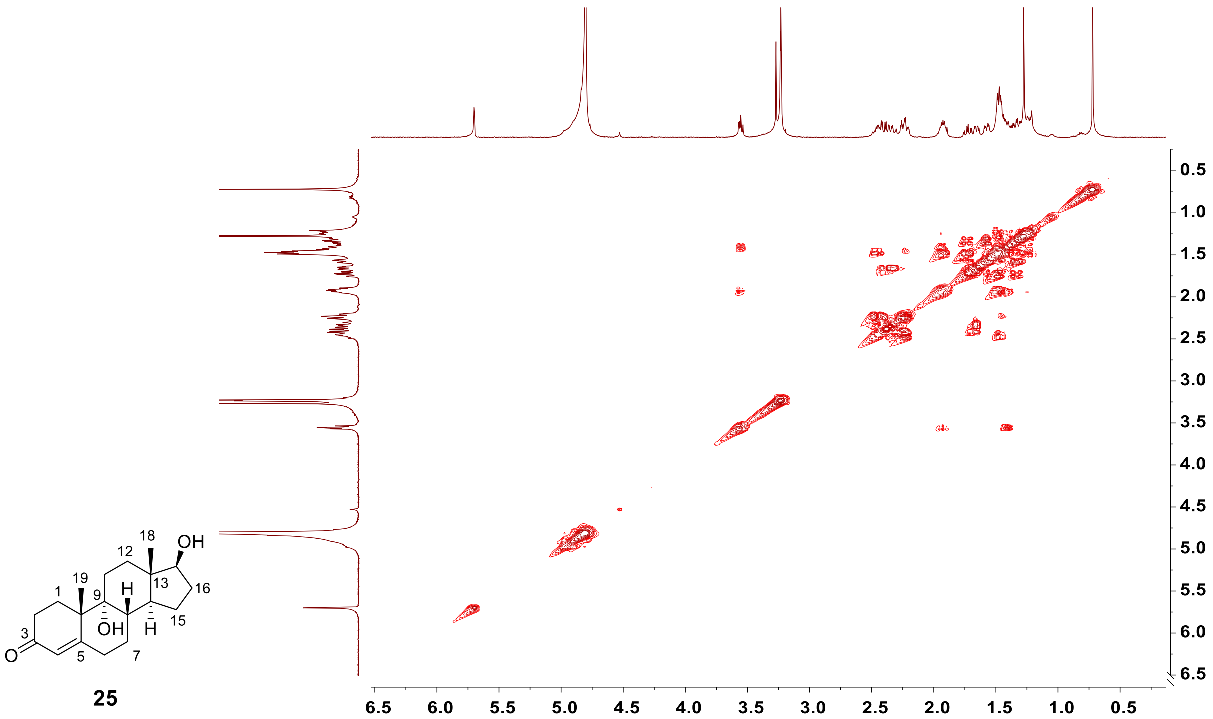

Supplementary Fig. 75 | COSY NMR spectrum of **25** in CD_3OD.

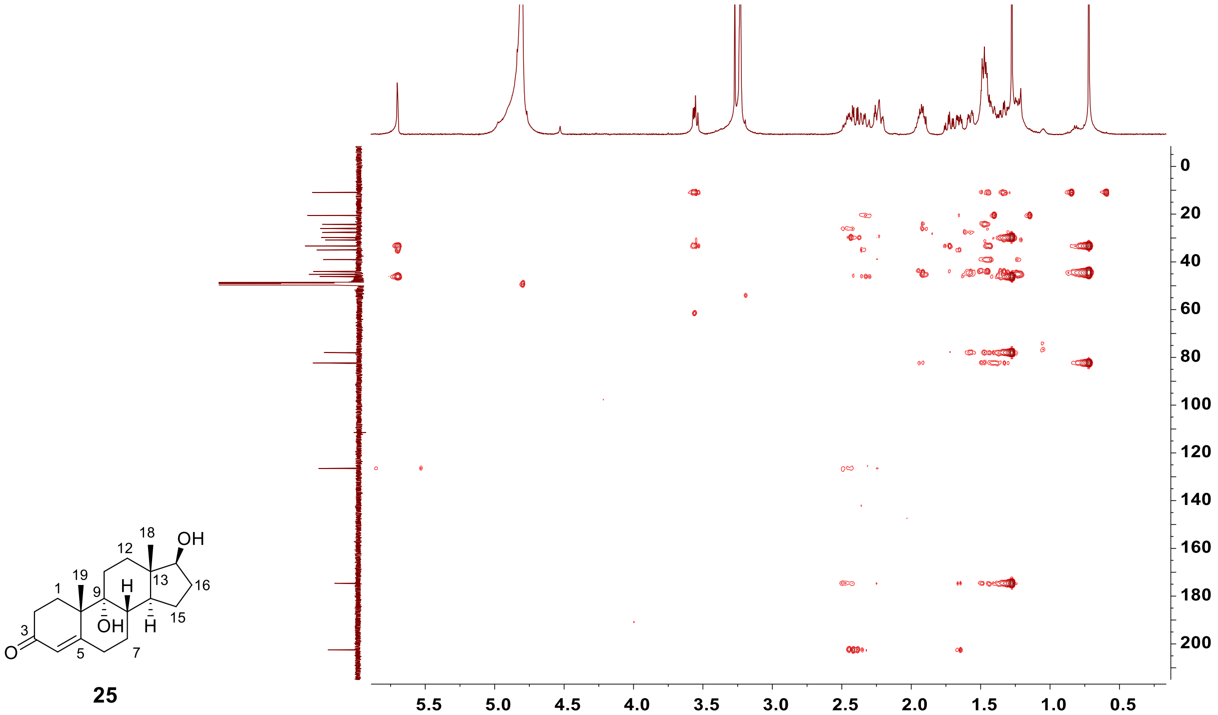

Supplementary Fig. 76 | HMBC NMR spectrum of **25** in CD_3OD.

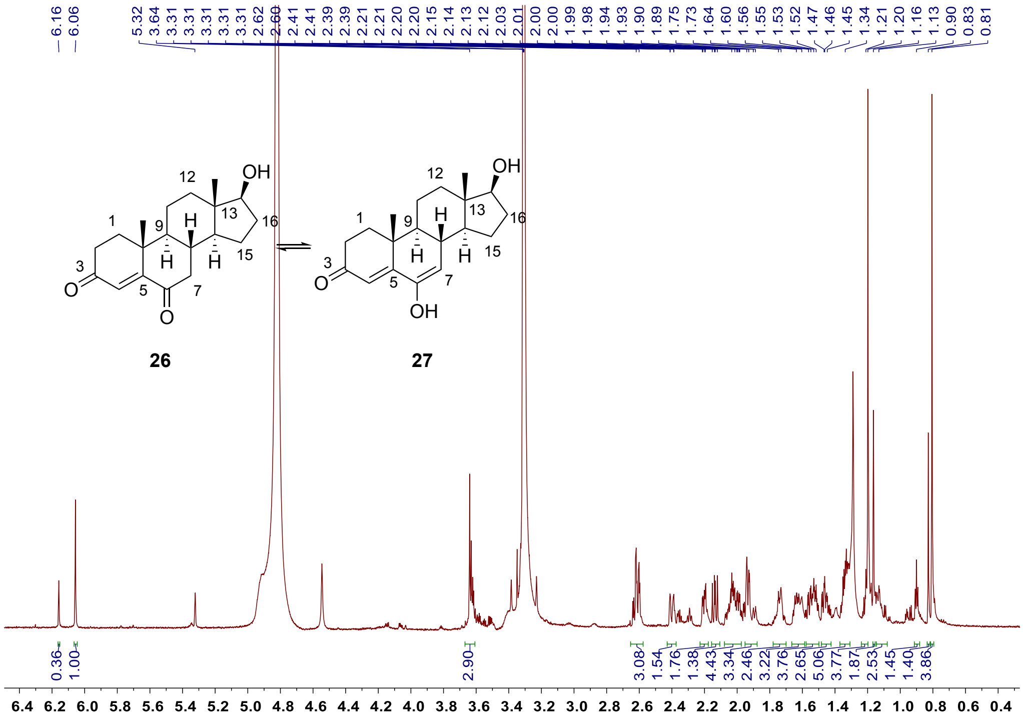

Supplementary Fig. 77 | **^{1}H NMR spectrum of **26/27 in CD_3OD.

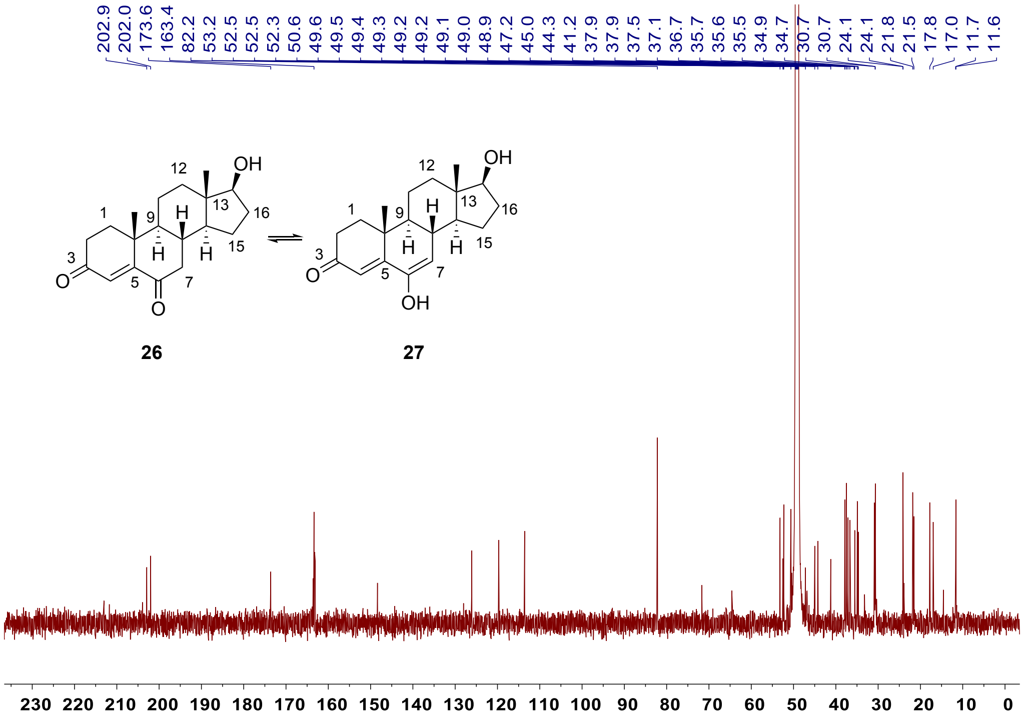

Supplementary Fig. 78 | **^{13}C NMR spectrum of **26/27 in CD_3OD.

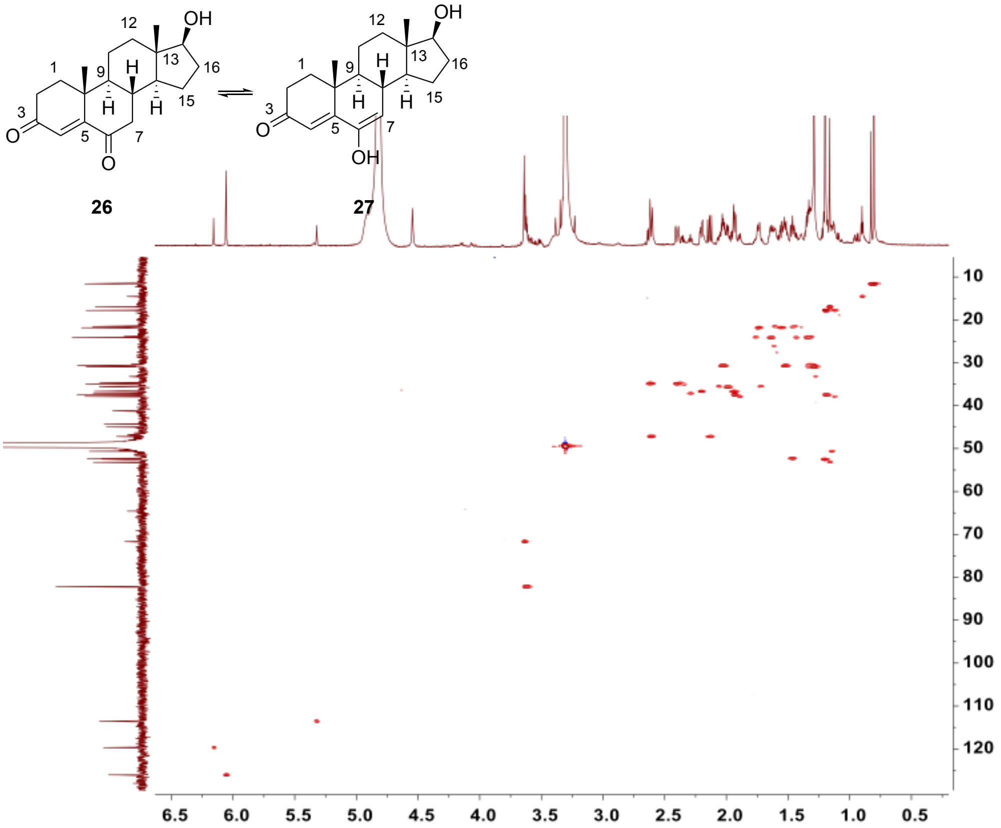

Supplementary Fig. 79 | ** HSQC NMR spectrum of **26/27 in CD_3OD.

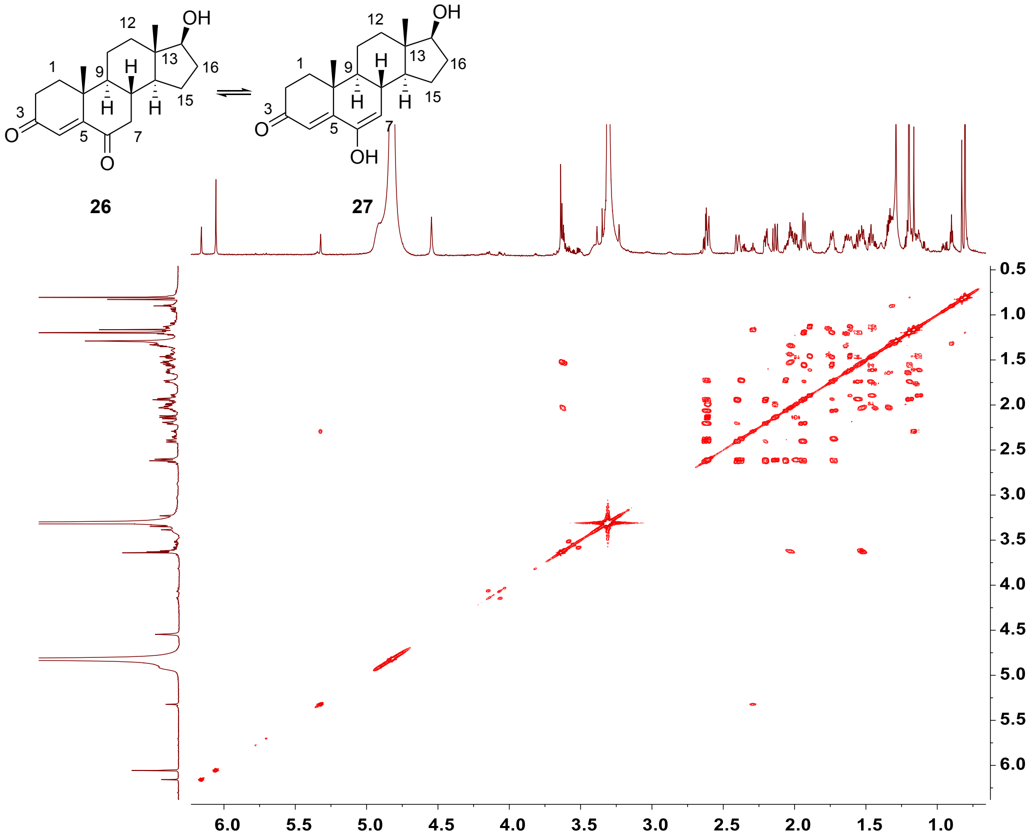

Supplementary Fig. 80 | ** COSY NMR spectrum of **26/27 in CD_3OD.

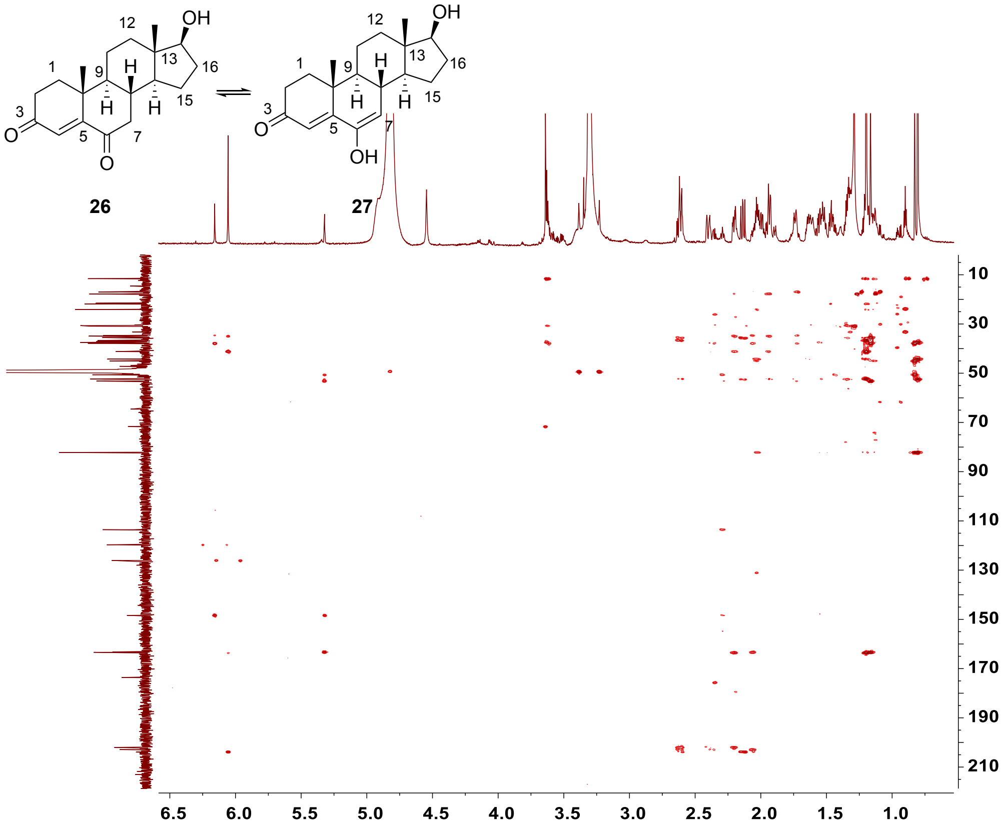

Supplementary Fig. 81 | HMBC NMR spectrum of **26/27** in CD_3OD.

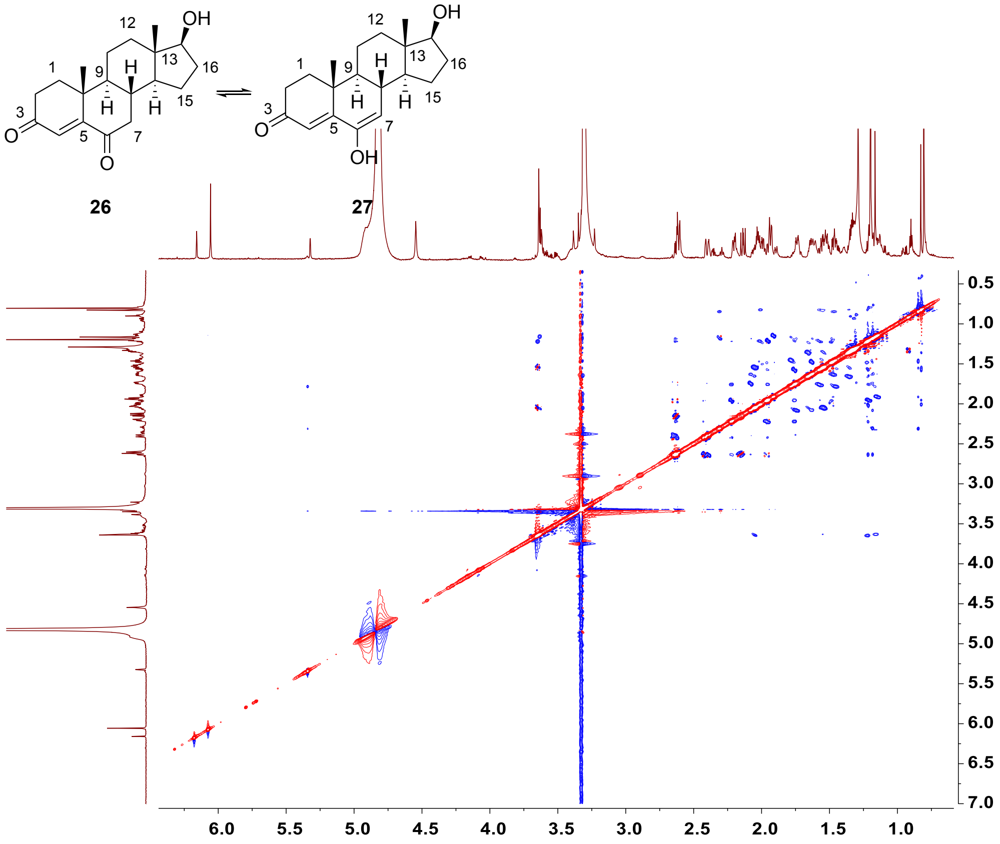

Supplementary Fig. 82 | NOESY NMR spectrum of **26/27** in CD_3OD.

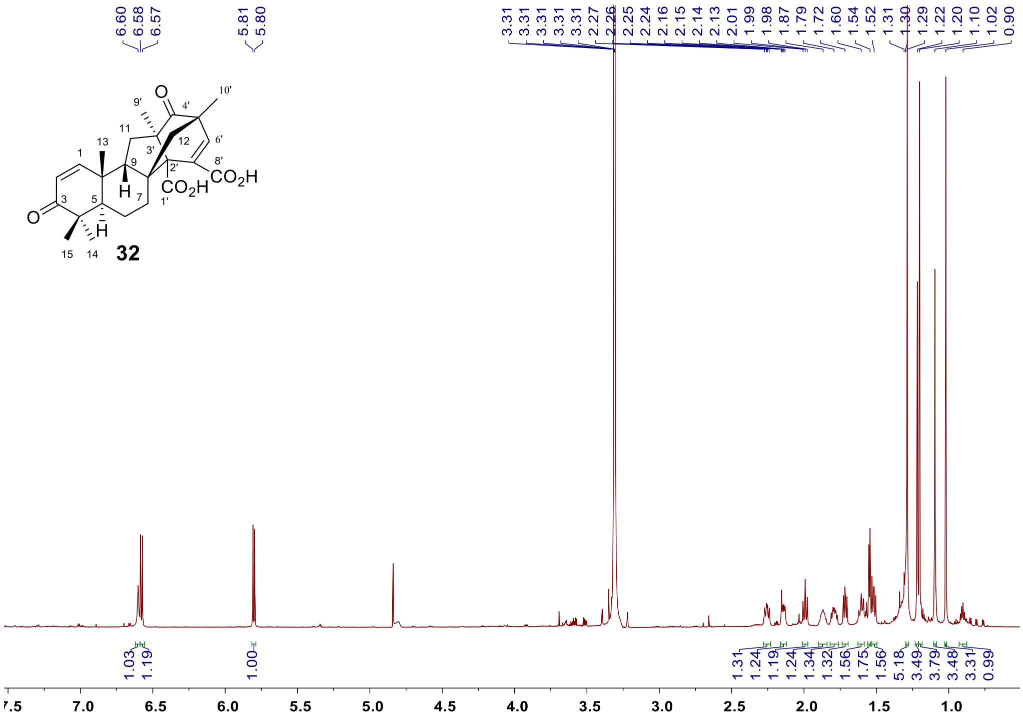

Supplementary Fig. 83 | **^{1}H NMR spectrum of **32 in CD_3OD.

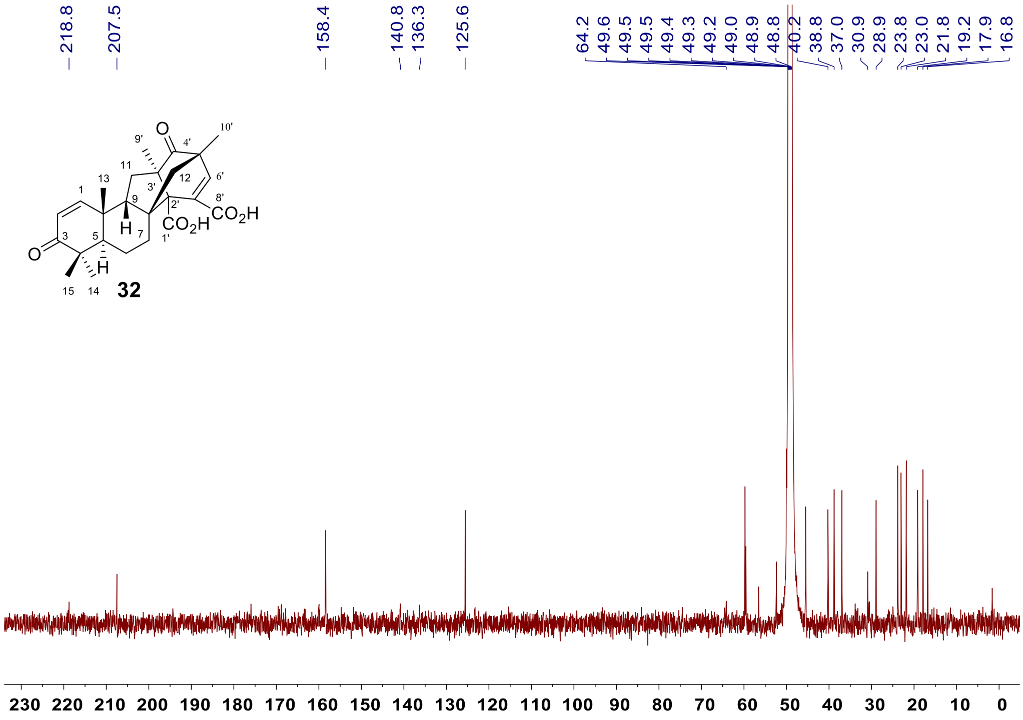

Supplementary Fig. 84 | **^{13}C NMR spectrum of **32 in CD_3OD.

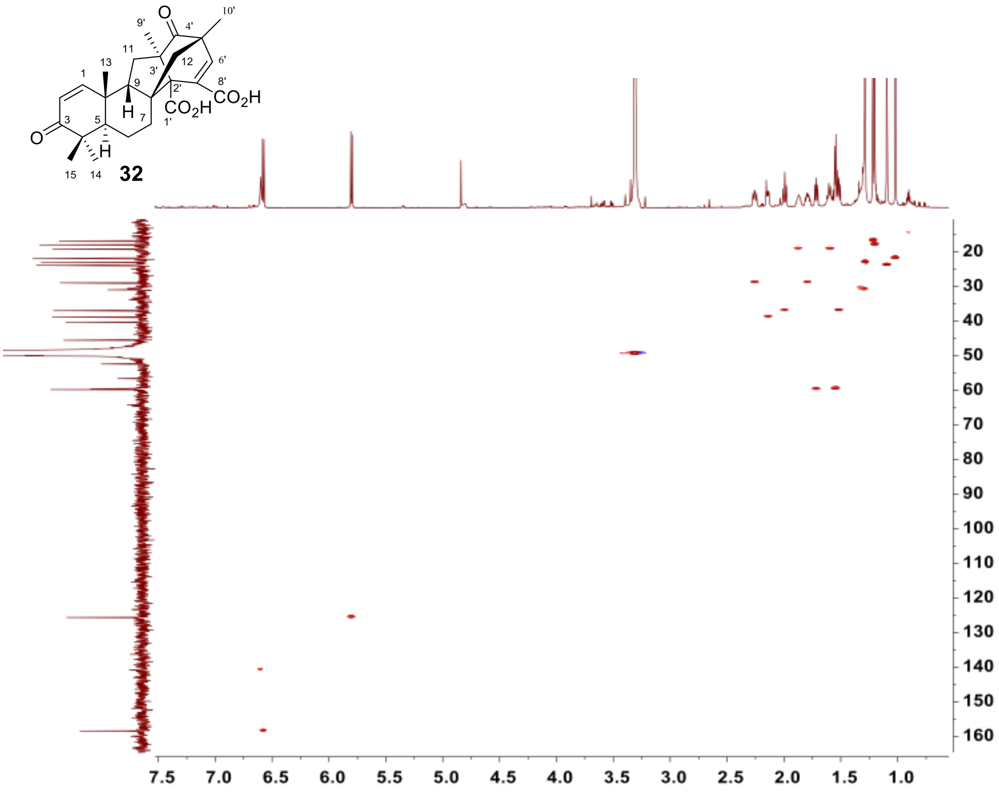

Supplementary Fig. 85 | ** HSQC NMR spectrum of **32 in CD3OD.

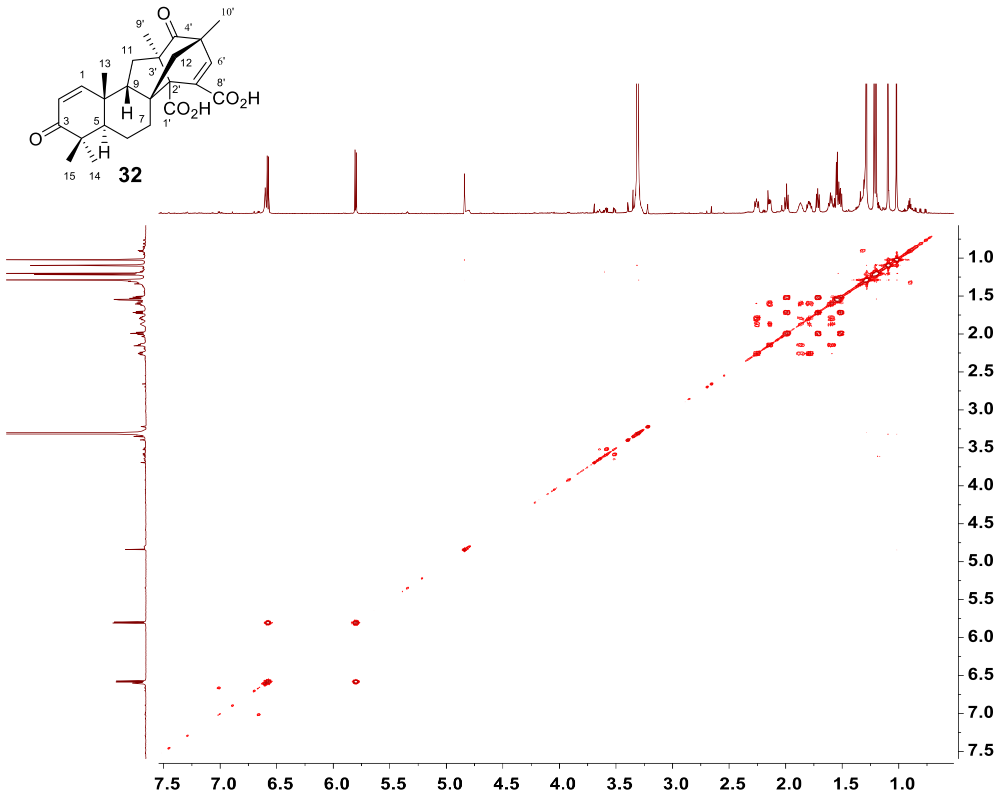

Supplementary Fig. 86 | ** COSY NMR spectrum of **32 in CD3OD.

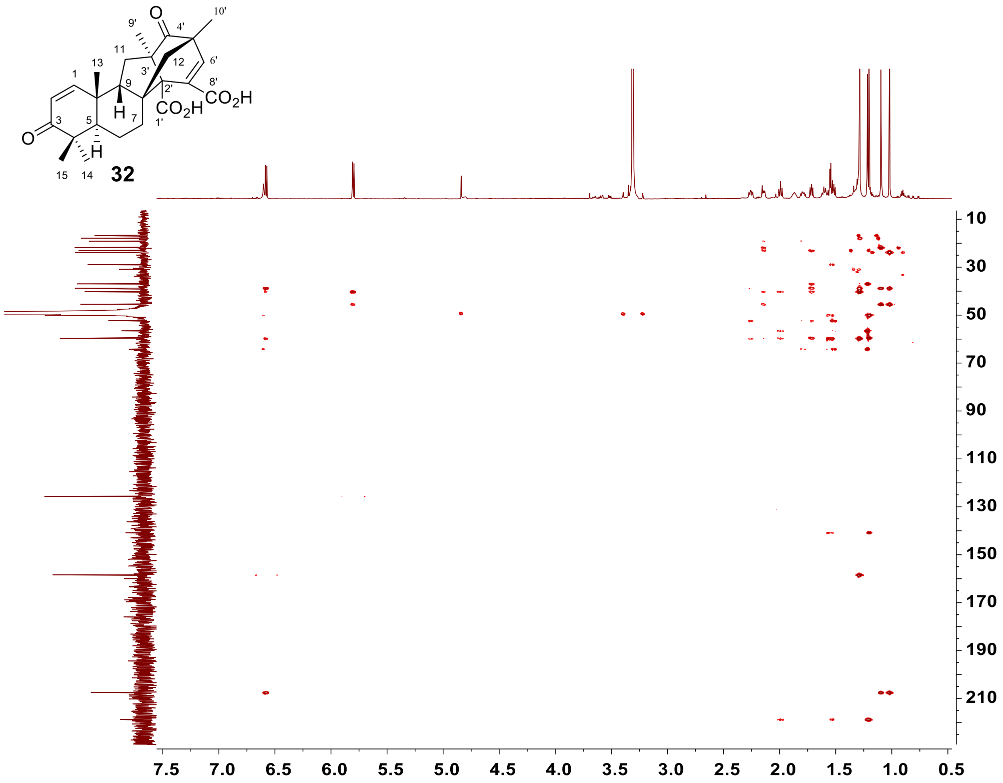

Supplementary Fig. 87 | ** HMBC NMR spectrum of **32 in CD3OD.

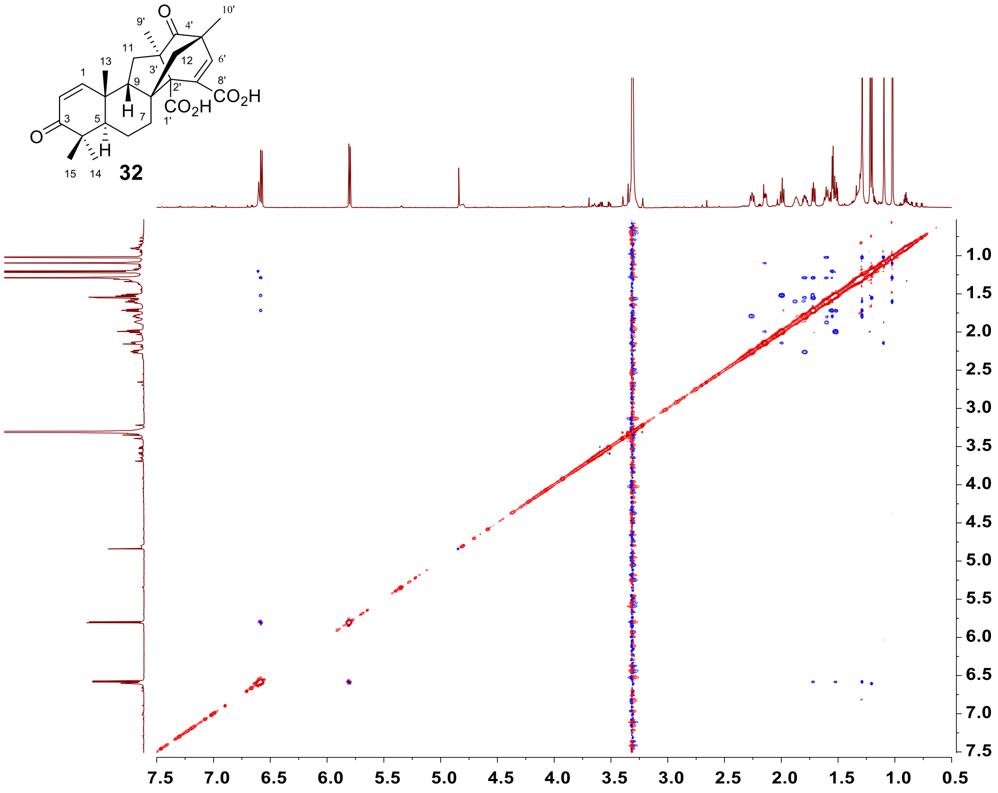

Supplementary Fig. 88 | ** NOESY NMR spectrum of **32 in CD3OD.

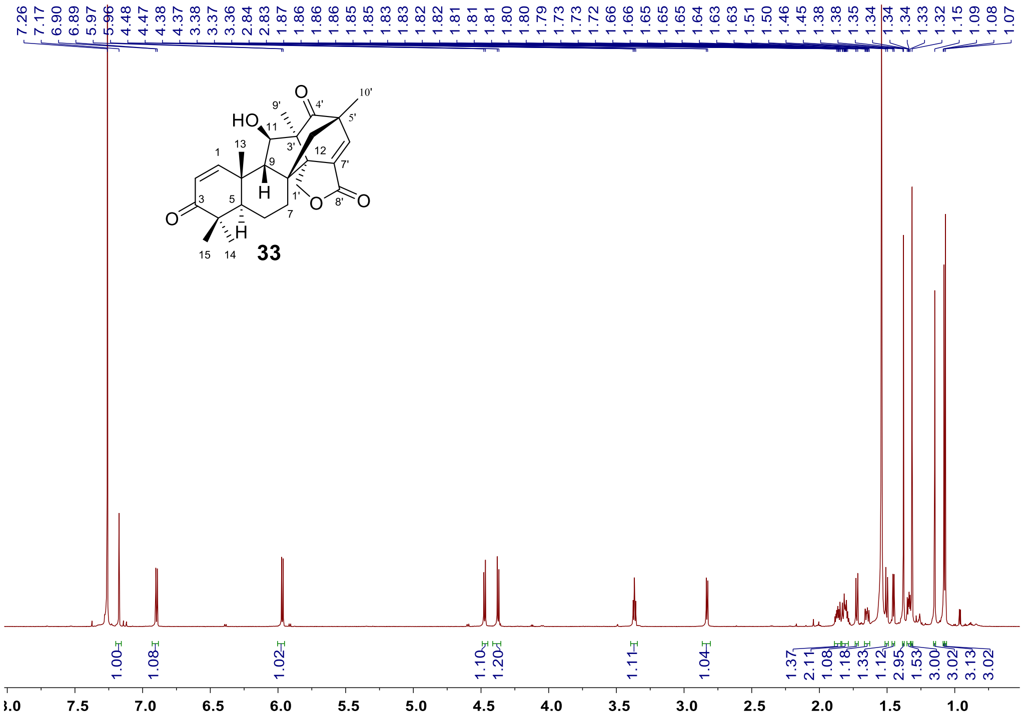

Supplementary Fig. 89 | ** ^{1}H NMR spectrum of **33 in CDCl3.

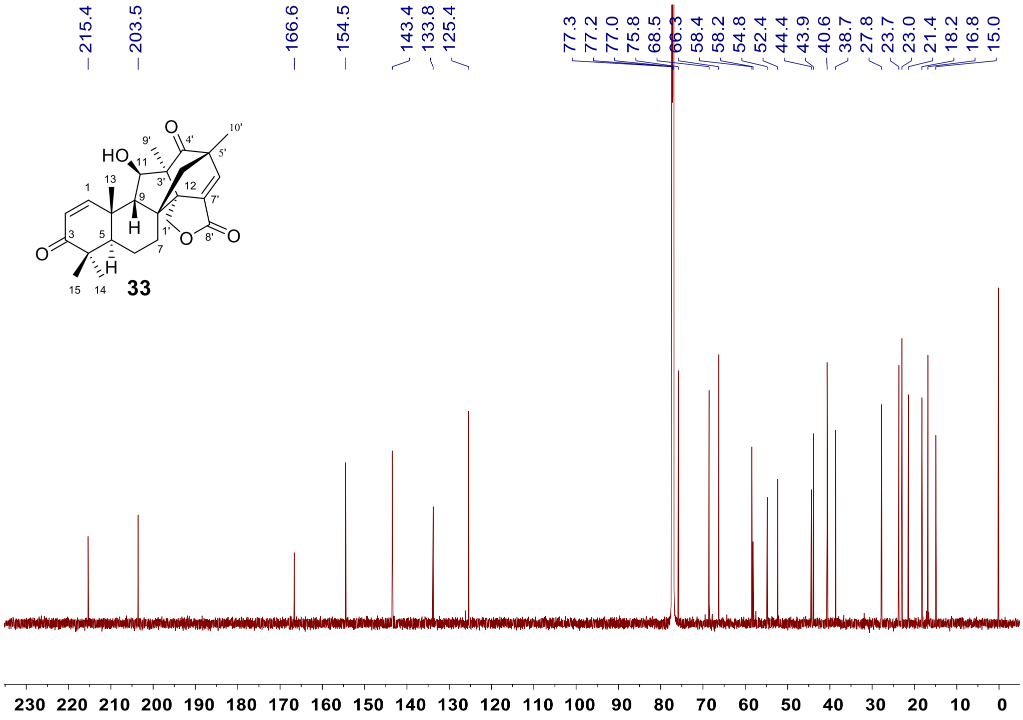

Supplementary Fig. 90 | ** ^{13}C NMR spectrum of **33 in CDCl3.

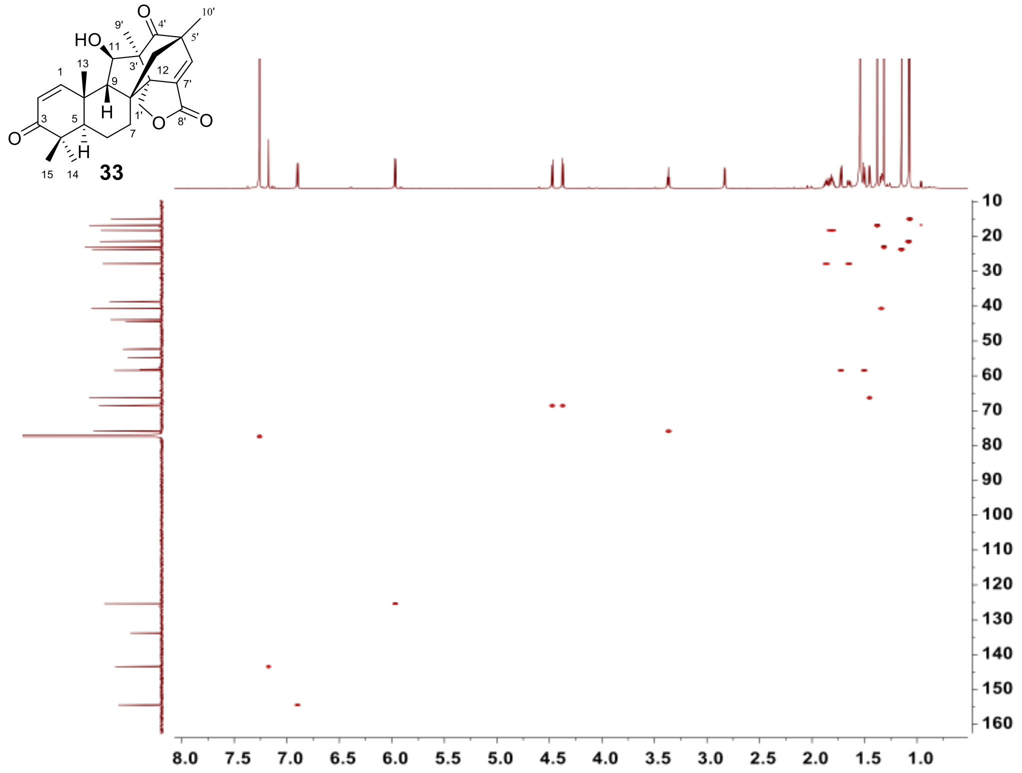

Supplementary Fig. 91 | ** HSQC NMR spectrum of **33 in $CDCl_3$.

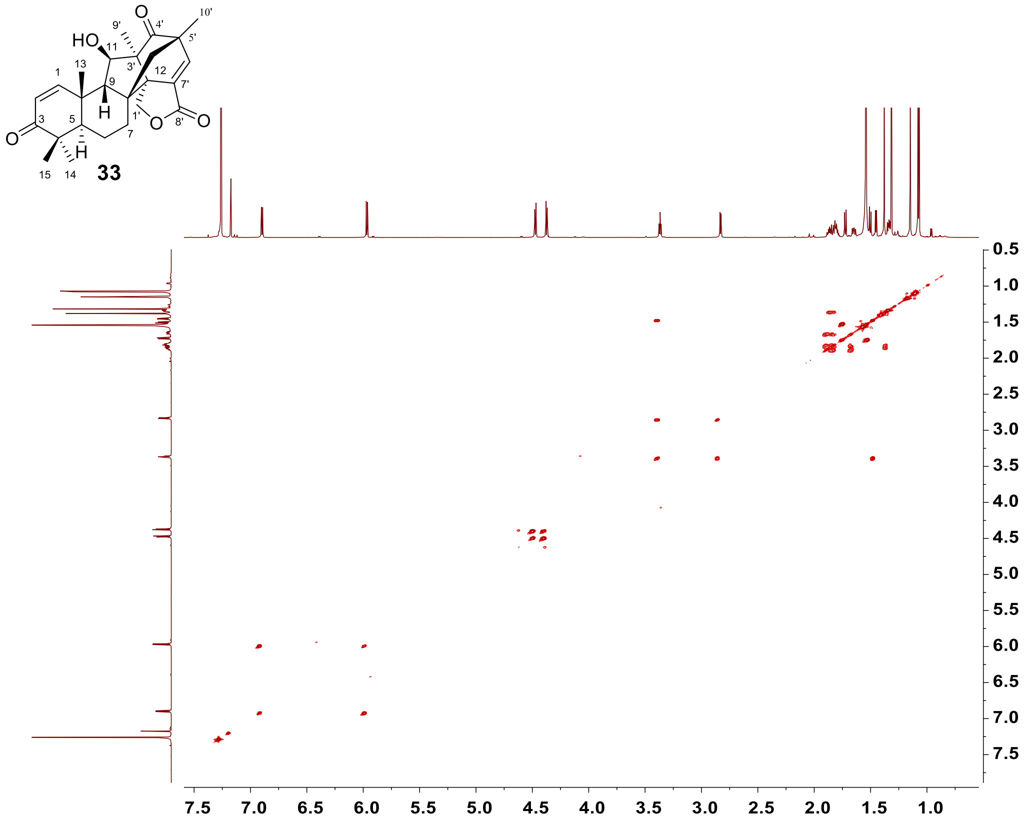

Supplementary Fig. 92 | ** COSY NMR spectrum of **33 in $CDCl_3$.

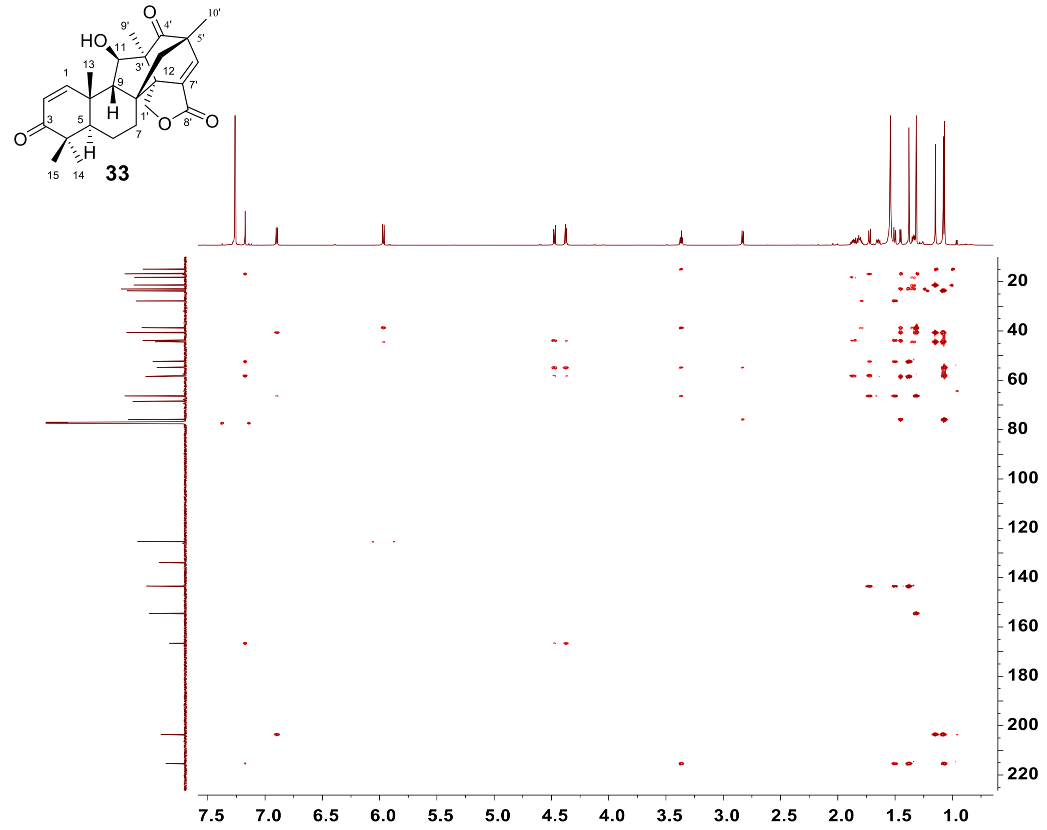

Supplementary Fig. 93 | HMBC NMR spectrum of **33** in $CDCl_3$.

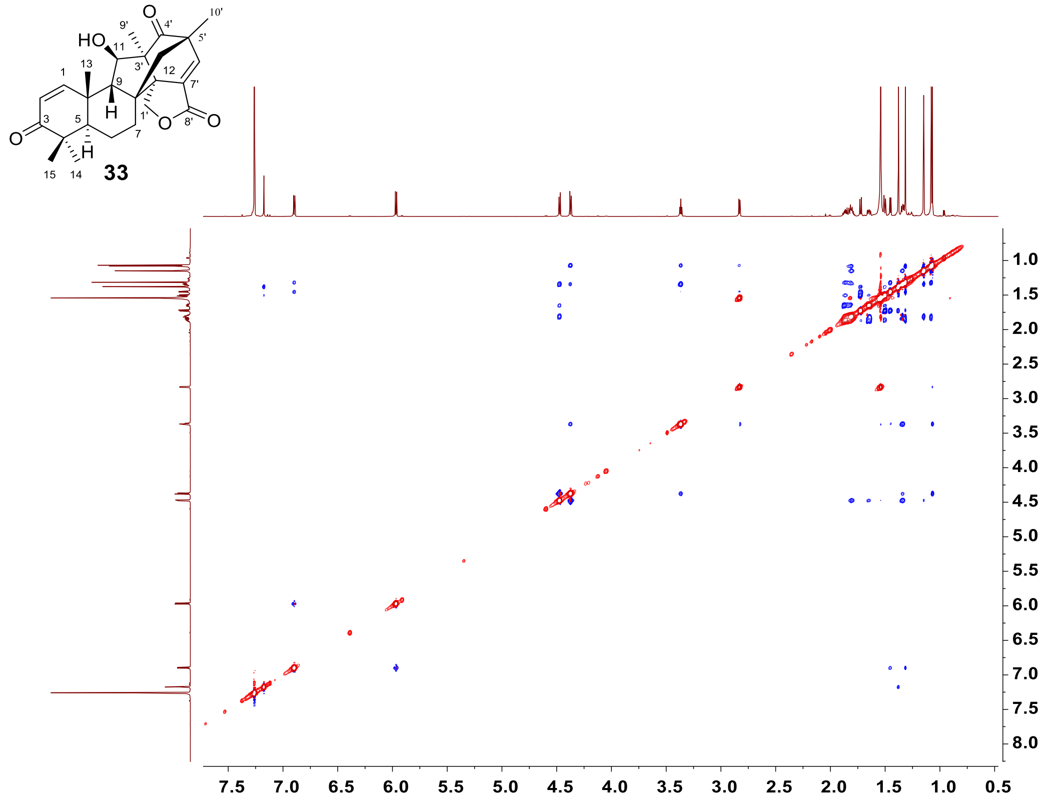

Supplementary Fig. 94 | NOESY NMR spectrum of **33** in $CDCl_3$.

Reference

1 Bai, T. *et al.* Structural Diversification of Andiconin-Derived Natural Products by α-Ketoglutarate-Dependent Dioxygenases. *Org. Lett.* **22**, 4311-4315 (2020).
