# Supplementary material for: Molecular insights into the unusually promiscuous and catalytically versatile Fe(II)/α-ketoglutarate-dependent oxygenase SptF
Source: Nat Commun. 2022 Jan 10;13:95. doi: 10.1038/s41467-021-27636-3 (PMC8748661; doi:10.1038/s41467-021-27636-3)
Supplement: Supplementary file 3 — Reporting Summary [file 41467_2021_27636_MOESM3_ESM.pdf]

nature research

Corresponding author(s): Ikuro Abe

Last updated by author(s): Nov 18, 2021

Reporting Summary

Nature Research wishes to improve the reproducibility of the work that we publish. This form provides structure for consistency and transparency in reporting. For further information on Nature Research policies, see Authors & Referees and the Editorial Policy Checklist.

Statistics

For all statistical analyses, confirm that the following items are present in the figure legend, table legend, main text, or Methods section.

n/a	Confirmed	
☐	☒	The exact sample size (*n*) for each experimental group/condition, given as a discrete number and unit of measurement
☐	☒	A statement on whether measurements were taken from distinct samples or whether the same sample was measured repeatedly
☒	☐	The statistical test(s) used AND whether they are one- or two-sided<br>*Only common tests should be described solely by name; describe more complex techniques in the Methods section.*
☒	☐	A description of all covariates tested
☐	☒	A description of any assumptions or corrections, such as tests of normality and adjustment for multiple comparisons
☐	☒	A full description of the statistical parameters including central tendency (e.g. means) or other basic estimates (e.g. regression coefficient) AND variation (e.g. standard deviation) or associated estimates of uncertainty (e.g. confidence intervals)
☒	☐	For null hypothesis testing, the test statistic (e.g. *F*, *t*, *r*) with confidence intervals, effect sizes, degrees of freedom and *P* value noted<br>*Give P values as exact values whenever suitable.*
☒	☐	For Bayesian analysis, information on the choice of priors and Markov chain Monte Carlo settings
☒	☐	For hierarchical and complex designs, identification of the appropriate level for tests and full reporting of outcomes
☒	☐	Estimates of effect sizes (e.g. Cohen's *d*, Pearson's *r*), indicating how they were calculated

Our web collection on statistics for biologists contains articles on many of the points above.

Software and code

Policy information about availability of computer code

Data collection	No software used
Data analysis	Prism 9, PHENIX-ver 1.19-4158, ccp4-7.1, XDS ver Feb 5 2021, Coot 0.9, PyMOL ver 2.0.6, SHELXT ver. 2014/5, SHELXL ver. 2018/1, CrysAlisPro, ver. 1.171.38.46

For manuscripts utilizing custom algorithms or software that are central to the research but not yet described in published literature, software must be made available to editors/reviewers. We strongly encourage code deposition in a community repository (e.g. GitHub). See the Nature Research guidelines for submitting code & software for further information.

Data

Policy information about availability of data

All manuscripts must include a data availability statement. This statement should provide the following information, where applicable:

- Accession codes, unique identifiers, or web links for publicly available datasets
- A list of figures that have associated raw data
- A description of any restrictions on data availability

The data supporting the findings of this study are available within the article and its Supplementary Information Files, source files or from the corresponding authors on request. The crystallographic data that support the findings of this study are available from the Protein Data Bank (http://www.rcsb.org). The coordinates and the structure factor amplitudes for the apo structures of SptF wild type apo, SptF wild type in complex with 1 and α-KG, SptF wild type in complex with 6 and NOG, and SptF N65T variant in complex with 1 and NOG, SptF S114A variant in complex with 15 and NOG, and SptF delta9 variant in complex with NOG were deposited under accession code 7EYR [http://doi.org/10.2210/pdb7EYR/pdb], 7EYS [http://doi.org/10.2210/pdb7EYS/pdb], 7EYT [http://doi.org/10.2210/pdb7EYT/pdb], 7EYU [http://doi.org/10.2210/pdb7EYU/pdb], 7EYW [http://doi.org/10.2210/pdb7EYW/pdb], and 7FCB [http://doi.org/10.2210/pdb7FCB/pdb] respectively. The crystallographic information file (CIF) for crystal structure of 25 has been deposited to The Cambridge Crystallographic Data Centre (CCDC), under reference number 2090716.

Field-specific reporting

Please select the one below that is the best fit for your research. If you are not sure, read the appropriate sections before making your selection.

☒ Life sciences ☐ Behavioural & social sciences ☐ Ecological, evolutionary & environmental sciences

For a reference copy of the document with all sections, see nature.com/documents/nr-reporting-summary-flat.pdf

Life sciences study design

All studies must disclose on these points even when the disclosure is negative.

Sample size	For all statistical experiments, including kinetics analysis and conversion rate values were determined with n=3 as it is common practice in the field, exemplified in Nature, 527, 539-547 (2015) and J. Am. Chem. Soc. 141, 9964–9979 (2019).
Data exclusions	No data are excluded.
Replication	All attempts are performed independently and successfully replicated more than three times. We stated the number of replicates for each experiment in the paper. Furthermore, the reproducibility of the assays was confirmed by including appropriate positive and negative controls.
Randomization	This is not relevant to our study because during the in vitro and structural analysis of target enzymes, the products, catalyzed reaction, and structure of enzymes were not known.
Blinding	Blinding is not relevant for this study because we use enzymatically synthesized substrates and mutant enzymes for in vitro reactions. During these analysis, the products, catalyzed reaction, and structure of enzymes were not known. Further, corresponding structures characterized by NMR which is an unbiased technique.

Reporting for specific materials, systems and methods

We require information from authors about some types of materials, experimental systems and methods used in many studies. Here, indicate whether each material, system or method listed is relevant to your study. If you are not sure if a list item applies to your research, read the appropriate section before selecting a response.

Materials & experimental systems

n/a	Involved in the study	
☒	☐	Antibodies
☒	☐	Eukaryotic cell lines
☒	☐	Palaeontology
☒	☐	Animals and other organisms
☒	☐	Human research participants
☒	☐	Clinical data

Methods

n/a	Involved in the study	
☒	☐	ChIP-seq
☒	☐	Flow cytometry
☒	☐	MRI-based neuroimaging
